# Factors associated with posttraumatic stress and anxiety among the parents of babies admitted to neonatal care: a systematic review

**DOI:** 10.1186/s12884-024-06383-5

**Published:** 2024-05-09

**Authors:** Reem Malouf, Sian Harrison, Victoria Pilkington, Charles Opondo, Chris Gale, Alan Stein, Linda S. Franck, Fiona Alderdice

**Affiliations:** 1 NIHR Policy Research Unit in Maternal and Neonatal Health and Care National Perinatal Epidemiology Unit Nuffield Department of Population Health, University of Oxford Old Road Campus Headington, Oxford, OX3 7LF UK; 2 Department of Medical Statistics, London School of Hygiene and Tropical Medicine, London, UK; 3 School of Public Health, Faculty of Medicine, Neonatal Medicine, Imperial College London, Chelsea and Westminster Campus, 369 Fulham Road, London, SW10 9NH UK; 4 Department of Psychiatry, Medical Sciences Division, University of Oxford, Oxford, UK; 5 Medical Research Council/Wits University Rural Public Health and Health Transitions Research Unit (Agincourt), School of Public Health, Faculty of Health Sciences, University of the Witwatersrand Honorary Professor, African Health Research Institute, Johannesburg, KwaZulu Natal South Africa; 6 African Health Research Institute, Durban, KwaZulu-Natal South Africa; 7 Department of Family Health Care Nursing, School of Nursing, University of California San Francisco, 2 Koret Way, San Francisco, CA 94143 USA; 8 School of Nursing and Midwifery, Queens University Belfast, Belfast, UK

**Keywords:** Posttraumatic stress symptoms, Posttraumatic stress disorder, Anxiety, Neonatal units, Preterm birth, Factors, Systematic review

## Abstract

**Background:**

Posttraumatic stress (PTS) and anxiety are common mental health problems among parents of babies admitted to a neonatal unit (NNU). This review aimed to identify sociodemographic, pregnancy and birth, and psychological factors associated with PTS and anxiety in this population.

**Method:**

Studies published up to December 2022 were retrieved by searching Medline, Embase, PsychoINFO, Cumulative Index to Nursing and Allied Health electronic databases. The modified Newcastle–Ottawa Scale for cohort and cross-sectional studies was used to assess the methodological quality of included studies. This review was pre-registered in PROSPERO (CRD42021270526).

**Results:**

Forty-nine studies involving 8,447 parents were included; 18 studies examined factors for PTS, 24 for anxiety and 7 for both. Only one study of anxiety factors was deemed to be of good quality. Studies generally included a small sample size and were methodologically heterogeneous. Pooling of data was not feasible. Previous history of mental health problems (four studies) and parental perception of more severe infant illness (five studies) were associated with increased risk of PTS, and had the strongest evidence. Shorter gestational age (≤ 33 weeks) was associated with an increased risk of anxiety (three studies) and very low birth weight (< 1000g) was associated with an increased risk of both PTS and anxiety (one study). Stress related to the NNU environment was associated with both PTS (one study) and anxiety (two studies), and limited data suggested that early engagement in infant’s care (one study), efficient parent-staff communication (one study), adequate social support (two studies) and positive coping mechanisms (one study) may be protective factors for both PTS and anxiety. Perinatal anxiety, depression and PTS were all highly comorbid conditions (as with the general population) and the existence of one mental health condition was a risk factor for others.

**Conclusion:**

Heterogeneity limits the interpretation of findings. Until clearer evidence is available on which parents are most at risk, good communication with parents and universal screening of PTS and anxiety for all parents whose babies are admitted to NNU is needed to identify those parents who may benefit most from mental health interventions.

**Supplementary Information:**

The online version contains supplementary material available at 10.1186/s12884-024-06383-5.

## Background

Having a baby admitted to a neonatal unit (NNU) can be highly distressing for parents [1, 2] and many experience mental health problems during and beyond their baby’s admission [3–5]. Evidence from a recent systematic review [5] estimated prevalence of anxiety among parents of babies admitted to NNU was as high as 42% during the first month after birth and remained high at 26% from one month to one year after birth. The prevalence of symptoms of posttraumatic stress (PTS) was equally high at 40% during the first month after birth, 25% from one month to one year and remained high at 27% more than one year after birth.

Unaddressed perinatal mental health problems can have long-term implications for parents, babies and families [6]. Identifying parents who are at risk of developing mental health problems during this vulnerable time is therefore vital so that timely support and interventions can be delivered [7]. However, it is unclear why some parents are more susceptible to develop mental health problems and others are more resilient. In the UK, women are asked about their emotional wellbeing routinely at each antenatal and postnatal contact with healthcare professionals [8]. For women in the general perinatal population, a number of factors are associated with perinatal anxiety. Obstetric factors include current or previous pregnancy complications, surgical obstetric interventions, and miscarriages; health and social factors include a history of mental health problems, domestic violence, being a single parent, having a poor couple relationship or inadequate social support [9–12]. PTS is associated with traumatic birth events including changes to birth plan, birth before arrival to hospital, emergency caesarean birth, instrumental vaginal birth, and manual removal of the placenta; third and fourth-degree perineal tears are additional risk factors for PTS after birth [13, 14]. The experience of childbirth in and of itself is an independent factor associated with PTS and therefore preterm birth and neonatal complications are considered as add-on stressors [15].

The factors associated with developing postnatal mental health problems in parents of babies admitted to NNU have received comparatively little attention and are poorly understood. It is unclear whether the factors associated with increased risk of mental health problems in the general perinatal population are applicable to parents of babies admitted to NNU, or whether there are different or additional factors for this population. Factors such as the unexpected nature of many NNU admissions, separation from the newborn, and concern about the infant’s health make the experience of parents with babies receiving neonatal care different from that of other parents. Therefore, it is important to understand the risk and protective factors for this specific population to ensure that approaches for assessment, detection and intervention for perinatal mental health problems are optimally delivered and, if necessary, appropriately tailored.

The aim of the review was to systematically collate, appraise and synthesise the current evidence on risk and protective factors for developing PTS and anxiety in parents of babies admitted to NNU.

## Methods

### Operational definitions

There is no formal or internationally agreed definition of NNUs. The UK Department of Health and Social Care’s definition includes special care units (SCUs), local neonatal units (LNUs) and neonatal intensive care units (NICUs) [16]. The American Academy of Paediatrics’ definition of NNUs include basic care (level I), specialty care (level II), and subspecialty intensive care (level III, level IV) [17]. Within the context of this review we included studies on parents of babies admitted to any level of NNU.

The Diagnostic and Statistical Manual of Mental Disorders Fifth Edition (DSM-5) [18] defines anxiety disorders as disorders that share features of excessive fear and anxiety and related behavioural disturbance. ﻿PTS is associated with exposure to trauma. Acute Stress Disorder (ASD) occurs within four weeks of a traumatic event, while Posttraumatic Stress Disorder (PTSD) occurs when symptoms persist beyond one month. Throughout this review, the term ‘PTS’ is used to cover clinically significant ASD, PTSD or PTS symptoms and the term ‘anxiety’ is used to cover both clinically significant anxiety symptoms or disorders.

The review protocol was prospectively registered with PROSPERO (CRD42021270526) and reporting followed Preferred Reporting Items for Systematic Reviews and Meta-Analyses (PRISMA) guideline [19].

### Eligibility criteria

Studies published in any language which examined the potential association of at least one risk factor with PTS or anxiety and were conducted with parents (mothers, fathers and carers) of babies admitted to any level of a NNU in all countries were included. Studies focusing on specific groups such as parents with existing mental health conditions or parents of deceased babies were also considered for inclusion. All observational study designs were eligible.

### Search strategy and selection criteria

A comprehensive search strategy was developed and tested using a combination of free-text (title/abstract) keywords and MeSH subject terms to describe the key concepts of PTS/anxiety, parents and NNUs. The search covered the period from the inception of each database until December 2022. No restriction was applied to the electronic searches. The following databases were searched: Medline, Embase, PsychoINFO, Cumulative Index to Nursing and Allied Health literature, Web of Science, ResearchGate and Google Scholar; Grey literature was also searched including Ethos, Proquest Dissertations & Theses and OpenGREY. The reference lists of all included studies were also searched for additional eligible studies. The search strategy applied in Medline is shown in Appendix 1.

### Study selection and data extraction

All screening of titles, abstracts and full texts was conducted in Covidence [20]. A data extraction form was piloted on selected studies and was then employed for the remaining studies. Data on country, study design, aims, inclusion/exclusion criteria, characteristics of included parents and babies, PTS/anxiety measuring tools, assessment time, potential risk and protective factors relevant to PTS and anxiety, data analysis method and estimated effects for each risk factor were extracted. All screening and data extraction were independently performed by at least two reviewers (RM, VP, SH, FA). Any discrepancies were discussed and resolved by a third author (FA, SH). Authors were contacted when required information was missing or when full texts were not available (*N* = 16).

### Risk of bias assessment

The quality and certainty of evidence were assessed using a modified version of the Newcastle–Ottawa Scale [21]. The modified tool contains seven domains of bias relating to the following sources: selection, sampling, measurement of factors/outcome, analysis, selective reporting and attrition. Low, high or unclear risk rating was used to assess the potential bias for each domain.

### Data synthesis

Summary statistics were extracted from all studies, including number of participants, number of risk factors and data relevant to each risk factor identified. When results from univariable and multivariable analyses were reported, only the latter were extracted. Meta-analyses by exposures/risk factors were not feasible due to the variability in the measurement of similar risk factors across studies (e.g. type of measurement tool, cut-off point, categorical or continuous data). Therefore, results were narratively synthesized and reported for PTS and anxiety separately.

## Results

**Fig. 1:**
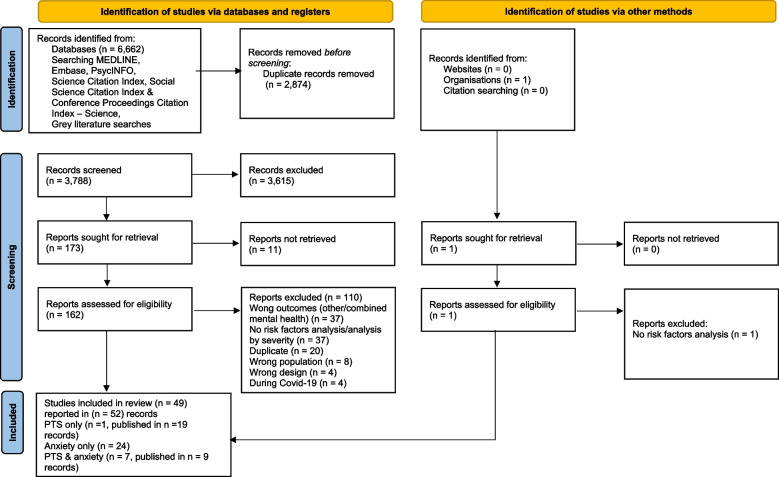
PRISMA flow chart of study selection

A total of 6,662 records were identified and, after removing duplicates, 3,788 records were screened, of which 3,615 records were excluded. 162 reports were assessed for full-text eligibility (11 reports could not be retrieved) and, of these, 110 reports were excluded with reasons and 49 studies, published in 52 records, were included. 18 studies, published in 19 records, reported on factors associated with PTS, 24 studies on anxiety and 7 studies, published in 9 records, reported on both, see Fig. 1.

## Post-traumatic stress (PTS)

### Description of the included studies

Table 1 presents the 25 studies published in 28 records [22–49] for PTS (including 7 studies reporting both PTS and anxiety). More than half of the studies were conducted in the USA [22, 25, 27–31, 34, 35, 41, 42, 44–47], five in Europe, published in six records [23, 26, 36, 37, 48, 49] two in Canada [32, 43], and one in each of the following countries: Australia [39], Argentina [40], Iran [38], South Korea [50] and Taiwan [24]. Six studies [24, 25, 27, 41, 44, 47] were of a cross-sectional design and the remaining studies were cohort studies.

**Table 1:** Characteristics of posttraumatic stress (PTS) included studies

Study ID, country	Study design & setting, study period, neonatal unit type of care, length of stay	Study objective	Study inclusion criteria	Study exclusion criteria	Parents’ characteristics	Babies’ characteristics
**Anchan 2021**, [22]** USA**	Prospective cohort,1 centre, June 2018-October 2019, NICU level 3, length of stay > 7 days	To determine the incidence of mental health symptoms in military families after NICU admission	Mothers and self-identified partners, > 18 yrs, English-speaking Department of Defense (DoD) Beneficiaries, infant remained admitted to NICU ≥ 7 days	Parents who were unable to maintain follow-up within military healthcare system beyond 7 days	*N* = 106 parents ( 66 mothers + 40 fathers) at 2 wks post birth (T1), *N* = 77 parents ( 53 mothers + 24 fathers) at 4–6 wks post NICU discharge (T2) Age – parents ≥ 35 yrs = 80 (75%) < 35 yrs = 26 (25%) SES—house income > $50.000 = 88 (83%) Ethnicity could select ≥ 1 White = 73 (69%), Other = 46 (31%) Education—college/trade school and higher = 74 (70%) LWP & parity = NR	N & BW = NR, GA = 35–38 and > 38 wks
**Brunson 2021,** [23] **France**	Prospective cohort, 3 centres, January 2008 and January 2011, NICU, length of stay = NR	To estimate the prevalence and predictive factors of mothers affected by posttraumatic symptoms after preterm birth	Mothers who delivered prematurely < 32 wks	Mothers with acute or chronic psychological illness, drug or alcohol abuse, underage, not speaking French For newborns life-threatening conditions, malformation and/or genetic abnormalities, and/or vulnerability of the baby evaluated by Perinatal Risk Inventory ≥ 10	*N* = 50 mothers Age = mean 30.9 ± SD 5.4 yrs Parity—nullips = 21 (42%) Education = graduated from high school = 40 (80%) SES, LWP & ethnicity = NR	*N* = 50, GA = mean 209.8 ± SD 10.9 days, BW = mean 1331 ± SD 350 g
**Chang 2016,** [24] **Taiwan**	Cross-sectional, 1 centre, January 2010-June 2011, length of stay < 60.00 ± 53.78 days, NICU level = NR	To estimate the prevalence of symptoms of distress in mothers of preterm NICU infants and factors complications of delivery for these symptoms	Mothers to babies < 37 wks gestation, admission to the NICU, and infant survival at the time of the interview	Mothers who did not understanding Chinese, refused to consent, babies with congenital chromosomal abnormalities/congenital defects, significant heart disease after birth, or died during the hospital stay or after leaving the NICU Mothers with major illnesses, cancer, or psychiatric disorders	*N* = 102 mothers Age = mean 34.28 ± SD, 4.45 Parity—nullips = 37 (36.27%) Education > 12 yrs = 95 (94.14%) SES—household income ≤ 600,000 NTD (about 19,679 USD) = 52 (50.98%) LWP & ethnicity = NR	*N* = 102, GA = 31.53 SD ± 2.97 wks, BW = 1661.86 ± SD 563.82 g
**Clark 2021,** [25] **USA**	Cross-sectional, 1 centre, July 2009-July 2014, NICU level IV, length of stay = NR	To study the associations between parents perceptions of infant symptoms and suffering and parent adjustment following the baby death	Parents of infants who died within the previous five yrs in level IV NICU	Age < 18 yrs, infants died within the past 3 months, not speaking English	*N* = 40 mothers, *N* = 27 fathers (27 mother-father dyads, 13 only mothers) Age – mothers = mean 33.33 ± 6 yrs Age – fathers = mean 36.74 ± 9.49 yrs LWP – mothers = 32 (80%) LWP – fathers = 24 (60%) Ethnicity – mothers = white 35 (88%) Ethnicity – fathers = white 16 (58%) Education – secondary—mothers = 34 (85%) Education—secondary – fathers = 18 (67%) SES—family income = range $50–75,000 for all Parity = NR	*N* = 40, BW & GA = NR
**Eutrope 2014,** [26] **France**	Prospective cohort, 3 centres, January 2008-January 2010, NICU, length of stay = NR	To clarify the relationship between the mother’s post-traumatic reaction and premature birth and the mother-infant interactions	Mothers to infants < 32 wks	For mothers: Psychiatric illness, drug or alcohol abuse, aged < 18 yrs, language barriers; For newborns: Unfavourable vital prognosis evaluated Perinatal Risk Inventory score ≥ 10 infants risk of significant developmental disabilities and malformation and/or genetic anomaly diagnosed	*N* = 100 mothers during 15 days after birth, N = 93 before NICU discharge Age = mean 329.8 ± 6 yrs Parity—nullips = 48 (48%) LWP = 92 (92%) Education – higher = 79 (79.29%) SES – employed = 69 (69%) Ethnicity = NR	*N* = 100, BW = mean 1320g, GA < 32 wks
**Garfield 2015** ^**a**^ [27], **USA**	Cross-sectional, 2 centres, length of stays = 31—211 days, mean = 93.1 ± SD 48.49 days, NICU level & period = NR	To identify risk factors among urban, low-income mothers, to enable NICU healthcare providers more effectively screening and referral	Mothers of VLBW < 1500 g and preterm < 37 wks, English peaking, no current mental health diagnosis, infants clinically stable and did not have a congenital neurological problems or symptoms of substance abuse	Mothers’ age < 18 yrs old, ongoing critical illness (HIV, seizure), major depression, psychosis, bipolar disease; mothers to infants receiving mechanical ventilation	*N* = 113 mothers Age = mean 24.7 ± SD 5.17 yrs LWP = 59 (52.3%) SES—received public aid = 44 (39%) SES – uninsured = 45 (40%) Ethnicity—African American = 92 (81%) Education—high school graduates = 49 (43%) Parity = NR	*N* = NR, GA < 37 wks, BW = mean 1,073 ± SD 342 g
**Greene 2015 & 2019 ** ^**a**^ [28], **USA**	Prospective cohort, 1 urban centre, 2011–2012, NICU level IV, length of stay = mean 91 ± SD 37 days	To analyse change of depression, anxiety and perinatal-specific PTS across VLBW infants’ first year of life and to identify predictors of these changes over time	English-speaking mothers, > 18 yrs, babies likely to survive and VLBW < 1500 g	NR	*N* = 69 at birth, *N* = 64 before NICU discharge Age = mean 27 ± SD 6 yrs Parity—nullips = 23 (34%) LWP = 32 (51%) Ethnicity—Black = 38 (54%), Non-Hispanic white = 18 (26%), Hispanic = 12 (17%), Asian = 1 (1%) Education – yrs of higher grade = 13.4 ± SD, 2.4 SES = NR	*N* = 69, GA = mean 27.5 ± SD 2 wks, range 23.2 to 32.3 wks, BW = mean 957 ± SD, 243 g
**Hawthorne 2016,** [30] **USA**	Prospective cohort, 8 centres, period = NR, 4 NICU level III, 4 PICU, length of stay = NR	To test the relationships between spiritual/religious coping strategies and grief, mental health and personal growth for parents to babies died in intensive care unit	Parents were eligible for the study if their deceased newborn was from a singleton pregnancy and lived for more than 2 h in the NICU or their deceased infant/child was 18 yrs or younger and a patient in the PICU for at least 2 h	Parents who did not speak English or Spanish, multiple gestation pregnancy if the deceased was a newborn, being in a foster home before hospitalization, injuries suspected to be due to child abuse, death of a parent due to illness/injury event	*N* = 165 both parents (114 mothers + 51 fathers) Age = mean mothers 31.1 ± SD 7.73 yrs, fathers = 36.8 ± SD 9.32 yrs LWP = mothers 84 (74%) LWP = fathers 43 (84%) SES – mothers employed = 63 (55%) SES- fathers employed = 32 (78%) Ethnicity – mothers white non -Hispanic = 22 (19%), black non-Hispanic = 50 (44%), Hispanic = 42 (37%) Ethnicity – fathers white non – Hispanic = 14 (28%) = black non-Hispanic = 16 (31%), Hispanic = 21 (41%), Education – mothers college degree = 35 (30%) Education – fathers college degree = 19 (37%) Parity = NR	*N* = 124 (69 NICU and 55 PICU), GA & BW = NR
**Holditch-Davis 2009** ^**a**^ [31], **USA**	Prospective cohort, 2 centres, NICU level & study period = NR	To examine inter-relationships among stress due to infant appearance and behaviour in the NICU exhibited by African American mothers of preterm infants	African American biological mothers of preterm infants < 1500 gm at birth or requiring mechanical ventilation. Mothers were recruited when their infants were no longer critically ill	Infants with congenital, symptomatic from substance exposure, hospitalized > 2 months post-term, or triplets or part of a higher order multiples set; mothers with no custody, follow-up for 2 yrs unlikely, HIV + , < 15 yrs, critically ill, not speak English, mental health problems	*N* = 177 mothers Age = mean 25.9 ± SD 6.5 yrs LWP = 46 (26.1%) SES—Public assistance = 92 (52.8%) Education = mean 12.6 ± SD 1.8 yrs Ethnicity = all African American Parity = NR	*N* = 190, mean GA = 28.3 SD ± 2.9 wks, mean BW = 1107 ± SD,394 g
**Jubinville 2012,** [32] **Canada**	Prospective cohort, 1 centre, February-May, 2008, NICU, level III, Length of stay = NR	To determine whether significant symptoms of (ASD) are present in mothers of premature NICU infants	Mothers of infants’ < 33 wks GA admitted to NICU	Infant with foetal anomaly, severe illness requires compassionate care and/ maternal illness precluded NICU visit and assessing women at 7–10 days after birth	*N* = 40 mothers Age = mean 29.2 ± SD 5.8 yrs LWP = 37 (93%) SES—income $60, 000 per year = 23 (58%) Ethnicity = majority white Education—above high school = 24 (60%) Parity = NR	*N* = 52, 10 twins, & one triplets, BW = mean 1374.5 ± SD 466.1 g range = 640–2220 g, GA = mean 29.0 ± SD 2.6 wks, range = 24.0–32.0 wks
**Kim 2015,** [33] **South Korea**	Prospective cohort, 1 centre, April to October 2009, NICU, level & length of stay = NR	To understand the progress and predictor factors of PTSD in mothers of high-risk infants	Mothers age of 18 to 45 yrs and who did not have a significant medical/surgical history that affected their performance on the self-report questionnaires	Mothers who did not speak Korean or problem executing the self-report questionnaires	*N* = 120 mothers (90 without PTSD + 30 0 with PTSD) Age—no PTSD = mean 31.87 ± SD 3.50 yrs, Age – PTSD = mean 31.83 ± 3.23 yrs LWP – no PTSD = 89 (98.9%) LWP – PTSD = 30 (100%) SES—employed—no PTSD = 34 (39.5%) SES – employed—PTSD = 14 (50.0%) Education—level ≤ 14 yrs no PTSD = 32 (36.0%) Education—level ≤ 14 yrs PTSD = 10 (33.3%) Ethnicity & parity = NR	*N* = NR, GA—no PTSD = mean 33.89 ± 3.76 wks GA – PTSD = mean 33.27 ± SD 3.91) wks, BW—no PTSD = mean 2.03 ± SD 0.78, BW—PTSD = mean 2.01 ± SD 0.72 kg
**Lefkowitz 2010,** [34] **USA**	Prospective cohort, 1 centre over a 9 months period, length of stay = median 14 days, NICU level = NR	To assess the prevalence and correlates of ASD and PTSD in mothers and fathers	Mothers and fathers of infants on NICU who were anticipated to stay on NICU > 5 days	Inability to read English, parent age < 18 yrs, or if the child’s death appeared imminent	*N* = 130 parents ( 89 mothers + 41 fathers) Age—mothers = mean 29 yrs Age—fathers = mean 33 yrs Ethnicity—mothers Caucasian = 61 (71%), Ethnicity—fathers Caucasian = 33 (81%) Education—mothers college degree = 21 (24.4%) Education—fathers college degree = 9 (21.4%) Parity, LWP & SES = NR	GA < 30 wks N & BW = NR
**Lotterman 2018** ^**a**^ [45], **USA**	Prospective cohort, 1 centre, NICU level III & IV, period & length of stay = NR	To investigate whether rates of psychopathology are elevated in mothers of moderate- to late preterm infants during/following infant hospitalization in the NICU, and associated protective and risk factors	Mothers of moderate- to late preterm infants GA 32 to < 37 wks	Mothers to babies born < 32 wks or later than 36 wks, or if they had been in the NICU for > 6 months	*N* = 91 mothers at NICU admission, *N* = 76 at 6 months Age = mean 32.45 SD ± 6.78 yrs Ethnicity = Caucasian 37 (40.7%), African American 15 (17.4%) Asian 9 (10.5%), American Indian/Alaskan Native 2 (2.3%), 27 (29.1%) other Education—mean yrs 14.29 ± SD 4.30 Parity, LWP & SES = NR	*N* = 91, GA = range 32–37 wks, GA = mean 33.53 ± SD, 1.33 wks, BW = NR
**Malin’s study, USA**						
Malin 2020 [46]	Cohort study, 1 centre, NICU – level IV, length of stay ≥ 14 days, period = NR	To determine if PTSD among parents after an NICU discharge can be predicted by objective measures or perceptions of infant illness severity	Parent of infants who were in NICU ≥ 14 days	Parents who did not speak English, infants discharged home with their non-biological parent, infant was previously discharged home or transferred to/from the cardiac ICU for surgery, infants who died in NICU	*N* = 164 parents LWP = 154 (94%) SES—government insurance = 82 (50%) Parity, ethnicity & education = NR	*N* = 164, GA = 23–28 wks (*n* = 36), 29–33 wks (*n* = 60), 34–36 wks (*n* = 29), > 37 wks (*n* = 39), BW < 1000 g (*n* = 28), BW > 1000 g (*n* = 136)
Malin 2022 [46]	Cohort study, 1 centre, September 2018-March 2020, NICU level IV, length of stay = mean 68.1 ± 65.6 days	To explore parents’ uncertainty during and after NICU discharge and the relationship between uncertainty and PTS	Parent of infants who were in NICU ≥ 14 days and had not previously been discharged from hospital	Parents not fluent in English, parents of infants whose death appeared imminent or who would be transferred to cardiac ICU before discharge; parents who would not be caring for infant post-discharge, parents of infants who died after enrolment, only one parent of each infant could participate	*N* = 319 parents during NICU, *N* = 245 parents at 3 months Age = mean 29.9 ± SD 5.59 yrs SES—unemployed = 52 (21%) Ethnicity = white 214 (67.3%), Black of African American 76 (24.0%), Asian 8 (2.5%), American Indian or Alaska Native 3 (0.1%), Other 17 (5.3%) Education—graduate 39 (12.2%) Parity & LWP = NR	*N* = 243, GA = 22–25 wks = 34 (10.7%), 26–28 wks = 37(11.6%), 29-31wks = 60 (18.8%), 32–36 wks = 119 (37.3%), ≥ 37wks = 69 (21.6%) range 22- ≥ 37 wks, BW = NR
**Misund 2013 & 2014** ^**a**^ **Norway** [36, 37],	Cohort study, 1 centre June 2005-July 2008, NICU level & length of stay = NR	To explore psychological distress, anxiety, and trauma related stress reactions in mothers experienced preterm birth and the predictors of maternal mental health problems	Mothers to preterm babies < 33 wks admitted to NICU	Mothers of severely ill babies that the medical staff estimated to have poor chance of survival, and non-Norwegian speakers	*N* = 29 mothers at 2 wks post birth, *N* = 27 at 2 wks after NICU admission, *N* = 26 at 6 & 18 months post term, Age = mean age 33.7yrs ± SD 4.3 yrs Parity—nullips = 18 (62.1%) LWP = all SES – unemployed = 4 (13.8%) Education > 12 yrs = 26 (89.7%) Ethnicity = NR	*N* = 35, GA = median 29, range 24–32) wks median BW = 1.2 kg (range 0.6–2.0), 40% twins
**Moreyra 2021** ^**a**^ **USA** [47],	Cross-sectional, October 2017-July 2019, length of stay at least 14 days, number of centres, period & NICU level = NR	To describe the impact of depression, anxiety, and trauma screening protocol and the referral pf positively screened NICU parents	Parents of NICU babies admitted at least for 2 wks	None excluded	*N* = 150 parents (120 mothers + 30 fathers) Age = 31.06 ± 6.26 yrs Parity—Para = mean 1.95 ± SD 1.2 LWP = 91 (61%) Ethnicity = white 39 26%, Other = 111 (74%) Education & SES = NR	*N* = NR, mean GA = 32.3 ± 4.8 wks, BW = mean 1935.2 ± SD 1052.1 g
**Naeem 2019, Iran** [38]	Cohort, 2 hospitals, 2016, NICU level & length of stay = NR	To compare the prevalence of PTS and its related risk factors in parents of hospitalized preterm and term neonates	Parents of NICU preterm (GA 24—36 wks) and parents to hospitalized terms (GA > 38 wks), inafnts’ age 2–5 days	History of psychological or psychotic problems with the experience of hospitalization, medication or psychiatric consultation, underlying diseases, and drug abuse	*N* = 160 parents (80 mothers + 80 fathers) Age – mothers = mean 33.78 ± SD 1.03 yrs Age – fathers = mean 37.14 ± SD 1.17 yrs LWP = all SES – employed = mothers 12 (15.25%) SES – employed = fathers 92.4% Education – mothers > high school = 72.2%, Education – fathers > high school = 52 (64.6%) Parity = NR	*N* = 80, GA = 24–36 wks, BW = NR
**Pace 2020, Australia** [39]	Prospective cohort, 1 centre, 2011–2013, NICU level & length of stay = NR	To report the proportion of parents of VPT infants with PTS symptoms at different time points	Families with very preterm infants, GA < 30 wks admitted to NICU	Parents who did not speak English, infants with congenital abnormalities, unlikely to survive	*N* = 105 parents (92 mothers and or 75 fathers) Age – mothers = mean 33 ± SD 5.3 yrs Age – fathers = 35 ± SD 6.2 yrs SES- high risk parents = 45 (43%) Education—mothers > 12 yrs = 62 (67%) Education – fathers > 12 yrs = 45 (60%) Parity, LWP & ethnicity = NR	*N* = 131, GA < 30 wks, GA = mean 27.8 ± SD 1.5 wks, BW = mean 1,038 ± 261 g
**Pisoni 2020** ^**a**^ ** Italy ** [49],	Prospective cohort, 1 centre, August 2013-April 2014, length of stay = mean 29, range 13–138 days, NICU level = NR	To examine maternal psychological, parental, perinatal infant variables and neurodevelopment	Preterm infants gestational age < 34 wks and their mothers age > 18 yrs old, speaking Italian	Congenital anomalies, infections, no psychiatric illness and/or drug abuse	*N* = 29 mothers Age = mean 32.79 ± 6.74 yrs Parity—nullips = 19 (65.52%) SES—employed = 25 (86.21%) Education = mean 14.31 ± 2.78 yrs LWP & ethnicity = NR	*N* = 29, GA = mean 30.23 ± SD 3.16, range 23–33 wks, BW = mean 528.9 5 ± SD 41.15 g, range 574–2327 g
**Rodriguez 2020, Argentina** [40]	Cohort, 1 centre, March 2014-November 2016, NICU level & length of stay = NR	To detect PTS frequency and symptoms among mothers of VLBW preterm < 32 wks	Mothers with singleton pregnancies to VLBW < 1,500g preterm babies < 32 wks	Mothers with psychiatric disorders before and/or during gestation, babies with chronic conditions & congenital malformations	*N* = 146 mothers Age = range ≤ 21 to ≥ 42 yrs Parity, LWP, SE, ethnicity & education = NR	*N* = 146, GA < 32 wks, BW < 1,500 g
**Salomè 2022, Italy** [48]	Prospective cohort, 1 centre, September 2018-September 2019, tertiary-level NICU, length of stay = NR	To determine the impact of parental psychological distress and psychophysical wellbeing on developing PTS at 1st yr post NICU discharge and any differences between mothers and fathers	Any couples to infants admitted to NICU during the study duration	NR	*N* = 40 parents (20 couples, 20 mothers + 20 fathers) Age – mothers = mean 34 ± SD 6.6 yrs range 27 to 49 yrs LWP = all Education—university degree = 5 (25%) Parity, SES & ethnicity = NR	*N* = 23, BW = mean 1,375 ± SD 458.57 g, range = 760–2500 g, GA = mean 31 ± SD 2.99 wks range = 25 to 36 wks
**Sharp 2021, USA** [41]	Cross-sectional – media survey, November 2015- July 2016, length of stay = 29.57 (26.79) days, number of centres and NICU level = NR	To report on maternal perceived stress to infants’ NICU admission and the relationship between traumatic childbirth and PTSD	Biological mothers ≥ 18 yrs old, USA residents, complete the survey in English, alive infants age 1–4 months	Completing < 75% of the survey, infants age > 1–4 months	*N* = 77 mothers Age = mean 39.6 ± 5.8 yrs Parity—nullips = 32 (41.6%) SES—unemployed = 26 (47%) Ethnicity = White 68 (88.3%), Hispanic 7 (9.1%) Education (Bachelor’s degree or above) = 35 (45%)	*N* = NR, BW < 2,500g = 47 (61.0%) , GA < 37 wks = 43 (55.8%)
**Shaw 2009, USA** [42]	Prospective cohort, 1 centre, NICU, length of stay = mean 12 ± 8 days, study period = NR	To describe the early-onset symptoms of ASD in parents and factors related to PTSD, identifying high-risk parents who may benefit from early intervention	English-speaking parents of NICU infants	NR	*N* = 40 parents (25 mothers + 15 fathers) Age – mothers = 34.55 ± SD, 4.41 yrs LWP = all SES – employed = mothers 18 (72%) SES – employed = fathers all Ethnicity – mothers = white 15 (60%) Ethnicity—fathers = white 12 (92.3%) Education—university and above = mothers 17 (52%) Education – university and above = fathers 12 (92%) Parity = NR	*N* = NR GA = 30.89 SD ± 4.11 wks, range = 27 to 41 wk, BW = mean 1,664.39 ± SD, 908.21 g range = 1052 to 4004 g
**Vinall 2018, Canada** [43]	Cohorts, 1 centre, July 2012 and March 2016, length of stay = mean 57.89 ± SD 35.87 days, NICU level = NR	To examine whether the number of invasive procedures together with mother’s memory for these procedures were associated with PTSS at discharge from the NICU	Mothers of infants < 37 wks GA	Infants were excluded if they had major congenital anomalies, were receiving opioids, or underwent surgery	*N* = 36 mothers Age = median 31, IQR 27–36 yrs Education = median 5 IQR, 4–5 yrs, Parity, LWP, SES & parity = NR	*N* = 36, GA median (IQR) 32 (30–34) wks, BW = NR
**Williams 2021, USA** [44]	Cross sectional, 1 centre, over 6 months, date = NR, level IV NICU, length of stay = 44.82 ± 51.37 days	To evaluate acute stress disorder (ASD) symptoms and their predictors in NICU mothers	English speaking biological mothers	Mothers with infants with brief lengths of stay	*N* = 119 mothers SES—Medicaid insurance = 85 (71.8%) Ethnicity = African American 58 (48.7%), Caucasian 47 (39.5%), Hispanic/Latinos 10 (7.6%), Asians 3 (2.5%) Education – college degree or higher = 41 (33.6%) Age, parity & LWP = NR	*N* = 115, GA = 33.2 ± 4.66 < 28 wks to > 37 wks, BW = 2278.43 ± 1037 g

*Abbreviations*: *ASD* Acute stress disorder, *BW* Birth weight, *FT* Full term, *GA* Gestational age, *HIV* Human immunodeficiency virus, *IQR* Interquartile range, *T1* Time one, *T2* Time two, *LWP* Living with partner (married or cohabit), *NICU* Neonatal intensive care unit, *NR* Not reported, *Nullips* Nulliparous, *N* Number of parents, *PTSD* Post-traumatic stress disorder, *PT* Preterm, *SD* Standard deviation, *SES* Socio-economic status, *wks* Weeks, *yrs* Years, *VLBW* Very low birth weight, *VPT* Very preterm

^a^Studies included in both post-traumatic stress and anxiety: Garfield 2015 [27], Greene 2015 & 2019 [28, 29] Holditch-Davis 2009 [31], Lotterman 2018 [45], Misund 2013 & 2014 [36, 37] Moreyra 2021 [47], Pisoni 2020 [49]

Two studies included bereaved parents of babies who had been admitted to NNU [25, 30] and one study [22] focused entirely on military families. Both parents were included in ten studies, published in 11 records [22, 25, 30, 34, 35, 38, 39, 42, 46–48] and only mothers were enrolled in the remaining studies. Gestational age (GA) of the infant was an inclusion criterion in nine studies published in ten records [23, 24, 26, 32, 36–39, 43, 45], and birth weight (BW) was a criterion in two studies published in three records [28, 29, 31]. Two studies included both GA and BW in their inclusion criteria [27, 40]. All studies used standardised self-report scales.

### Risk of bias assessment

**Fig. 2:**
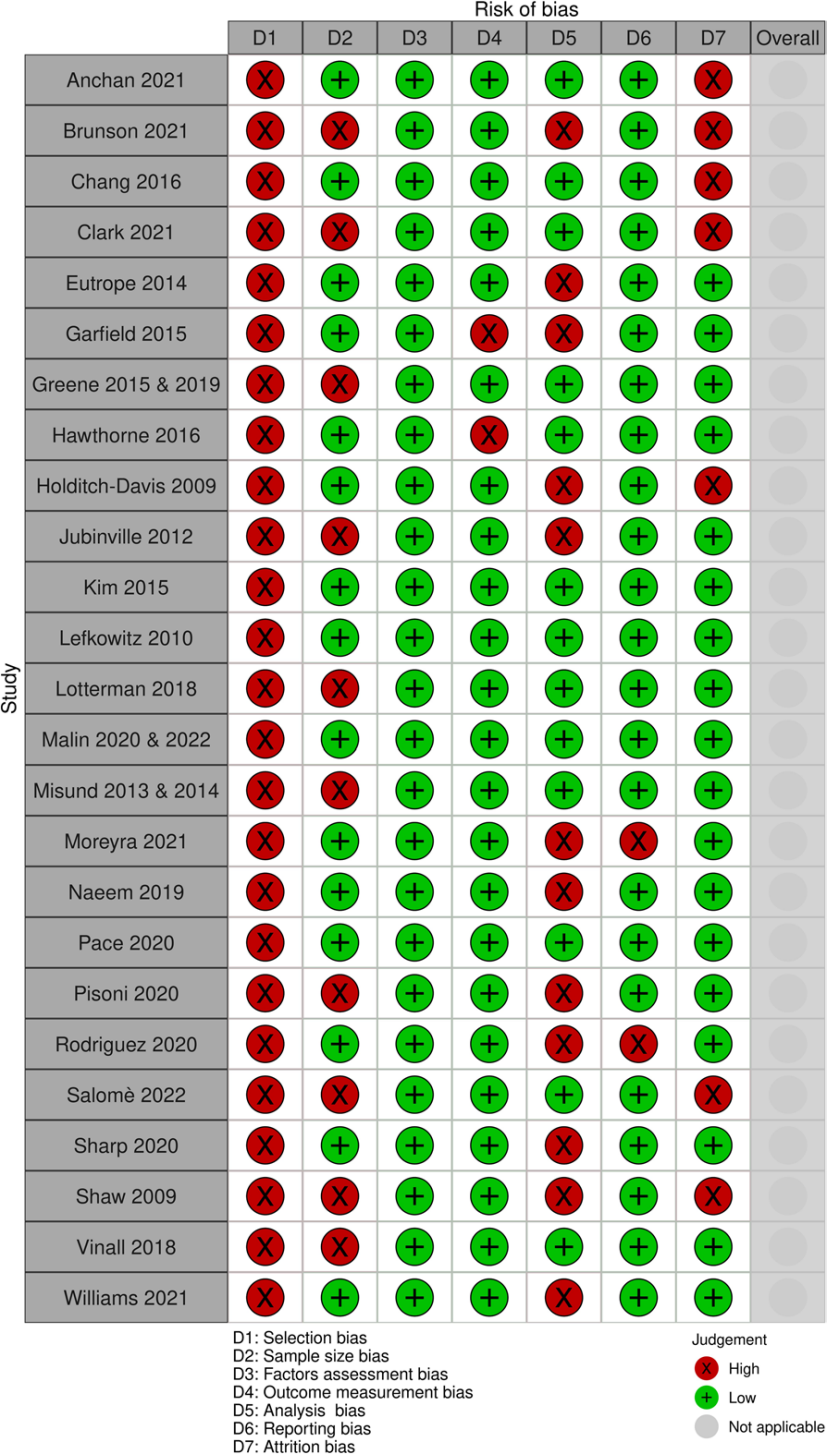
Risk of bias summary of post-traumatic (PTS) included studies

None of the included studies were at low risk of bias across all domains (see Fig. 2- A summary of risk of bias of PTS studies and Appendix 2). All studies had high risk of selection bias because all applied some exclusion criteria and most used convenience sampling. Ten studies, published in 12 records [23, 25, 28, 29, 32, 36, 37, 42, 43, 45, 48, 49], did not employ adequately powered sample sizes. Twelve studies [23, 26, 27, 31, 32, 38, 40–42, 44, 47, 49] had high risk of analysis bias due to unmeasured confounding factors or correlational analysis only, and seven studies [22–25, 31, 42, 47] had high risk of attrition bias due to low participation rates or high loss to follow-up. All except two studies [40, 47] had low risk of reporting bias. All studies were at low risk of bias for factor and outcome measurement.

### Factors associated with post-traumatic stress (PTS)

Overall, 2,506 parents were involved across the 25 included studies with sample sizes ranging from 29 to 245 participants. A total of 62 potential risk or protective factors were identified. The factors are detailed in Table 2, presented in a mapping diagram in Table 3 and summarised here under the following eight categories: parent demographic factors; pregnancy and birth factors; infant demographic factors; infant health factors; parent history of mental health symptoms; parent postnatal psychological factors; parent stress and coping, and other factors.

- 1) Parent demographic factors (Ten factors: age, education, sex, ethnicity, parents’ area deprivation, income, employment status, housing and access to transport, single parent, family social risk)

**Table 2:** Summary of factors reported in posttraumatic stress (PTS) included studies

**Study ID**	**Study population**	**Time of PTS assessment **	**PTS measuring tool and cut-off points**	**Parents with PTS, N, n (%) **	**Statistical analysis**	**Factors **(assessment time)								
**Anchan 2021**	Military families	2 wks after birth	Stanford Acute Stress Reaction Questionnaire (SASRQ) ≥3 score one at least 1 relevant item	Parents *N* = 106, *n* = 26 (24.5%)	Multivariable logistic regression	**Factors** (2 wks after birth)	**OR**						**95%CI**	
						Parent role alteration	1.19						0.47, 3.03	
						Parent sex	*1.20*						*0.47, 3.01*	
						Active military service	0.73						0.3, 1.79	
						**Pre-existing** **mental** **health** **disorders (PMHD) **	**3.32**						**1.07, 10.3**	
						History of significant family geographic separation (SFGS)	2.0						0.72, 5.54	
						GA ≤ 35 vs >35 wks	1.64						0.73, 3.72	
						Parent-reported infant illness severity	1.29						0.53, 3.12	
		4-8 weeks post discharge	PLC-5 screening in each PTSD symptom cluster (re-experiencing, avoidance, negative alterations in cognition and mood, and hyperarousal)	Parents *N* = 77, *n* = 6 (7.8%)	Multivariable logistic regression	**Factors **(4-6 wks post NICU discharge)	**OR**						**95%CI**	
						Parent role alteration	0.9						0.15, 5.26	
						Active military service	0.5						0.09, 3.16	
						PMHD	1.8						0.19, 17.96	
						History of significant family geographic separation (SFGS)	1.1						0.2, 6.75	
						GA	0.4						0.1, 2.2	
						Parent-reported infant illness severity after admission (1 vs >1)	2.1						0.37, 12.65	
						Parent-reported infant illness severity after discharge (1 vs >1)	3.7						0.68, 20.4	
						Positive screening for ASD (SASRQ)	2.0						0.34, 12.26	
						Positive screening for depression after admission (PHQ-2)	2.0						0.34, 12.26	
		4-8 wks post NNU discharge	PCL-5	Parents *N* = 77, *n* = 28 (36.4%)	Multivariable logistic regression	**Factors **(4-8 wks after NICU discharge)	**OR**						**95%CI**	
						Parental role – alteration	2.94						0.95, 9.09	
						Active military service	0.62						0.24, 1.59	
						Pre-existing mental health disorders** (**PMHD)	1.88						0.43, 8.17	
						History of significant family geographic separation (SFGS)	1.72						0.64, 4.68	
						Gestational age (GA)	0.87						0.3, 2.12	
						Parent-reported infant illness severity after admission 1 vs >1	2.06						0.8, 5.31	
						**Parent-reported infant illness severity after discharge 1 vs >1**	**3.88**						**1.29, 11.69**	
						**Positive screening for ASD (SASRQ)**	**8.44**						**2.38, 29.96**	
						**Positive screen after admission - Public Health Questionnaire (PHQ-2)**	**5.69**						**1.72, 18.83**	
**Brunson 2021**	Mothers to babies < 32 wks	18 months after birth	mPPQ ≥ 19	Mothers *N* = 50, *n* = 18 (36%)	Univariate parametric tests	Reported: **HADS** **depression** at 18 months ***P*** **=** **0.02,** **HADS** **anxiety** at 18 months ***P*** **=** **0.02** Primiparous, In vitro fertilisation, Multiple pregnancy, Threatened preterm labour, C-section, Psychological support, graduated from high school p >0.05.								
						Mothers mPPQ ≥19 VS <19								
						**Factors **(18 months after birth)	**Mean difference**					**95%CI**		
						**Gestational age in days**	***-6.40***					***-12.36, -0.44***		
						BW kg	*-0.19*					*-0.39, 0.00*		
						PRI score at birth	*0.80*					*-0.67, 2.27*		
						Maternal age	*2.50*					*-0.33, 5.33*		
						**Factors**	***OR***					***95%CI***		
						Male sex	*1.19*					*0.37, 3.79*		
						Apgar score < 7 at 5min	*0.83*					*0.25, 2.72*		
						Graduated from high school	*1.28*					*0.30, 5.54*		
						Primparous	*1.39*					*0.43, 4.56*		
						IVF	*0.40*					* 0.06, 2.62*		
						Multiple pregnancy	*0.58*					*0.14, 2.37*		
						Threatened preterm labour	*2.22*					*0.57, 8.68*		
						**CS**	***3.49***					***1.01, 12.05***		
						**HADS anxiety > cut off**	***4.33***					***1.03, 18.18***		
						HADS depression > cut off	*3.24*					*0.61, 17.31*		
						Psychological support	*1.92 *					*0.31, 12.05*		
**Clark 2021**	Parents to deceased preterm	3 months to 5 years after infant death Mean 8.65 months (SD, 16.9)	IES-R ≥ 33	Mothers *N* = 40, *n* = 7 (18%)	Multivariable hierarchical linear regression	**Factors **(3 months-5 years after death)	**B**					***P*** ** value**		
						Step 1								
						**Education**	**-0.31**					**< 0.05**		
						Income	-0.15					NS		
						Step 2								
						Medical Interventions (Chart)	0.12					NS		
						Step 3								
						**Infant Symptoms - Mother**	**0.46**					**< 0.01**		
				Fathers 27, 3 (11%)	Multivariable hierarchical linear regression	**Factors**	**B**					***P*** ** value**		
						Step 1								
						**Income**	**-0.35**					**< 0.05 **		
						Step 2								
						**Infant Suffering - Father**	**0.60**					**< 0.01**		
						*Parent sex OR 1.70, 95%CI 0.40, 7.24*								
**Eutrope 2014**	Mothers to preterms born < 32 wks	Visit 1 (v1) within 15 days after birth and visit 2 (v2) before NICU discharge	mPPQ ≥ 19	Mothers *N* = 100, mean 16.4 ± SD 9.9	Correlation	**Factors **( at v1 and v2)				**r**		***P*** ** value**		
						**State of health of the child (Perinatal Risk Inventory) **				**0.25**		**0.04**		
						**State of health of the child (PRI v2 - PRI v1)**				**0.25**		**0.04**		
						**Increase BW**				**-0.23**		**0.03**		
						**HADS depression score assessment within 15 days after birth (v1)**				**0.54**		**< 0001**		
						**HADS depression score assessment before discharge (v2)**				** 0.48**		**< 0001**		
						**HADS anxiety score v1**				**0.54 **		**< 0001**		
						**HADS anxiety score v2**				**0.52**		**< 0001**		
						**HADS global score v1**				**0.60**		**< 0001**		
						**HADS global score v2**				**0.56**		**< 0.001**		
						**Satisfaction of perceived social support (SSQ ) v1**				**-0.23**		**0.03**		
						**SSQ v2**				**-0.22**		**0.04**		
						**Postnatal depression v2**				**0.50 **		**< 0.001**		
**Garfield 2015** ^**a**^	Low income mothers to very low BW < 1500 g	First 3 months after birth	PPQ ≥ 6	Mothers *N* = 113, *n* = 34 (30%)	Correlation	**Factors **(First 3 months after birth)				**r**		**P**		
						**State Anxiety**				**0.51 **		**< 0.01**		
						Maternal age > 35 yrs				-0.02		NS		
						NBRS				0.17		NS		
						Education				0.02		NS		
						**Parental stress**				**0.50**		**< 0.01**		
**Greene 2015 & 2019**														
Greene 2015^a^	Mothers to very low BW < 1500 g	T1 (mean 28.1 days after birth), T2 (mean 14.8 days prior to NICU discharge)	mPPQ ≥ 19	Mothers *N* = 69, T1=17 (25%), T2=16 (25%), T1 and/or T 2 =22 (34%)	Multivariable logistic regression	**Factors **(Mean 14.8 days before NICU discharge)	**OR**			**95%CI**			***P*** ** value**	
						**Previous Exposure to Traumatic Events**	**1.07**			**1.01, 1.13**			**< 0.05**	
						**Primiparaous**	**4.80**			**1.26, 18.31**			**< 0.05**	
Greene 2019^a^		T1 (mean 28.1 days after infants’ birth);T2 (mean 14.8 days prior to infants’ NICU discharge); T3 (infants’ 4 month CA follow-up visit); T4 (infants’ 8 month CA follow-up visit)		PPTS score at T1= mean 13.43	Multivariable hierarchical linear regression	**Factors **(At birth)	**Mean**			**95%CI**			***P***	
						**Increase BW g at birth**	**−0.02**			**−0.03, −0.01**			**0.005**	
						Older maternal age (years) at birth	-0.37			-0.84, 0.10			0.12	
						**Previous traumatic events (per event)**	**1.23**			**0.14, 2.3 **			**0.03**	
						**Neighbourhood poverty (z-score)**	**-30.92**			**-54.3, −7.5 **			**0.01**	
						**Factors **(Over 1^st^ year of life)	**Mean**			**95%CI **			**P **	
						Increase BW (g) at birth	0.001			-0.001, 0.002			0.16	
						Older maternal age (years) at birth	0.05			0.00, 0.11			0.05	
						Previous traumatic events (per event)	-0.06			-0.18, 0.07			0.36	
						Neighbourhood poverty (z-score)	1.73			−0.96, 4.40			0.20	
**Hawthorne 2016**	Bereaved parents after neonatal (NICU) or paediatric intensive care unit (PICU) death	1& 3 months after death	IES-R, cut-off = NR	Parents *N*=165 ( 114 mothers and 51 fathers), *n* = NR	Multivariable linear regression	**Factors **(1 & 3 months after death)	**β**			**95%CI**			***P***	
						**Spiritual activities at 1 & 3 months mothers**	−0.33 & −0.35			NR			< 0.01	
						Spiritual activities at 1 & 3 months fathers	−0.29 & −.07			NR			NS	
						Religious activities at 1 & 2 months mothers	−0.10 & −0.08			NR			NS	
						Religious activities at 1 & 2 months fathers	−0.12 & −0.03			NR			NS	
						Adjusted for race/ethnicity and religion								
**Holditch-Davis 2009**	African-American mothers < BW 1500 g infants	During NCU admission	PPQ ≥ 6	Mothers = 117, *n* = 50 (42.9%)	Correlation	**Factors **(During NICU)				***r***			***P*** ** value**	
						**PSS subscale: Infant appearance**				**0.49**			**< 0.001**	
						**PSS subscale: Parental role alteration**				**0.54**			**< 0.001**	
						**Depressive symptoms at enrolment**				**0.73**			**< 0.001**	
						**State anxiety at enrolment**				**0.55**			**< 0.001**	
**Jubinville 2012**	Mothers of infants born < 33 wks	One wk (T1) and one month after birth (T2)	Acute Stress Disorder Interview (ASDI) & Stanford Acute Stress Reaction Questionnaire (SASRQ) >37	Mothers *N* = 40 T1 ASDI *n*= 11 (28%), SASRQ *n* = 20 (50%) T2 *n* = 34 ASDI (18%) SASRQ *n*= 14 (41%)	Correlation	**Factors **(1 week after birth)				**r**			**P value **	
						**Depression**				**NR**			**0.001**	
**Kim 2015**	Mothers to infants born at GA mean 33.89 (SD 3.76) wks	when infants 1 month corrected age (CA), 3 months CA, 12 months CA	mPPQ ≥ 19	Mothers = 120, *n* = 33 (28%)	Multivariable logistic regression	**Factors **(1 year CA)	**aOR**			**95%CI**			***P*** ** value**	
						Age of mothers	1.13			0.93, 1.37			0.234	
						Low education of mothers (<14 years)	2.33			0.59, 9.13			0.226	
						**First-born baby**	**7.62**			**1.07, 54.52**			**0.043**	
						Twin	4.60			0.60, 35.43			0.143	
						GA at birth	0.98			0.81, 1.19			0.854	
						Apgar score, 5 min	1.02			0.59, 1.77			0.938	
						Emergency visit or rehospitalisation	2.82			0.79, 10.09			0.111	
						Adjusted odd ratio (OR) by all the other variables								
**Lefkowitz 2010**	Parents of NNU infants	3–5 days after the infant’s NICU admission	Acute Stress Disorder Scale (ASDS) ≥ 1 symptom Of dissociation, re- experiencing, avoidance and arousal	Parents *N* = 130, *n* = 42 (32%)	Correlation	**Factors** (During NICU)				***r***			***P*** ** value**	
						**Length of NICU stay**				**-0.21**			**< 0.05**	
						Parent age				-0.07			NS	
						Minority				0.07			NS	
						**Family history of depression**				**0.02**			**< 0.05**	
						Concurrent stressors				0.15			NS	
						Parent-rated medical severity				0.01			NS	
						Physician rated medical severity				-0.05			NS	
						*Parent sex*				OR 1.50			95%CI 0.58, 3.87	
		30 days post NICU admission	PTSD presence of ≥ symptom ((dissociation, re-experiencing, avoidance, and arousal) plus moderate level of impairment	*n* = 9 (11%)	Multivariable linear regression	**Factors **(At 30 days post admission)	**unstandardized B**			**SE**			**Β**	***P*** ** value**
						**Total ASDS score**	**0.31**			**0.053**			**0.47 **	** 0.00**
						**Family history of depression**	**6.07**			**2.21**			** 0.23 **	**0.008**
						**Family history of mental Illness**	**12.28**			**4.27**			**0.24 **	**0.005**
						**Number of concurrent stressors**	**2.56**			**0.86**			**0.23 **	**0.004**
						*Parent sex at 30 days OR 2.03, 95%C I 0.41, 10.15*								
**Lotterman 2018**	Mothers of moderate- to late-preterm infants	During NICU stay and 6 months later	PCL > 38	Mothers *N* = 91, *n* = 14 (15.40%), 6 months *n* = 15 (15.8%)	Multivariable linear regression	**Factors **(During NICU)	**B**			**SE**				***P*** ** value**
						**Previous Mental Illness (PMI)**	**124.23**			**55.73**				**≤ 0.05**
						Mother-Infant Contact (MI) (verbal and physical)	-1.39			0.99				NS
						**PMI x MI Contact**	**-12.45**			**5.93**				**≤0.05**
						Infant health problem (IHP)	9.48			11.88				NS
						Mother-Infant Contact (MI)	-1.34			1.34				NS
						IHP x M-I Contact	-0.79			1.33				NS
						Forward-Focused (FF) Coping	-0.66			0.54				NS
						NICU mother-Infant (MI) Contact ( physical and or verbal)	-5.18			4.07				NS
						FF x MI	0.05			0.06				NS
					Linear regression at 6 months	**Factors **(At 6 months)	**B**			**SE**				***P*** ** value**
						Baseline PTSD	0.18			0.18				NS
						Mother visits per week	-0.49			1.49				NS
						Positive mother-nurse interaction	2.13			4.10				NS
						Mother-understands explanations	-4.47			3.31				NS
						Mother-technical questions	-0.92			2.34				NS
						Mother-asks how to baby care	1.66			3.70				NS
						**Optimism**	**-0.43**			**0.22**				**≤ 0.05**
						**Length of Stay (days)**	**0.6**			**0.3**				**≤ 0.05**
						**Mother-Infant Contact/NICU visit**	**-2.67**			**0.76**				**< 0.001**
						**Infant Health Problems by the mothers**	**2.0**			**0.87**				**≤ 0.05**
						Pessimism about baby’s recovery (Baseline)	0.1			0.14				NS
						**Length of Stay (days) X Pessimism**	**0.01**			**0.0**				**< 0.001**
**Malin 2020 & 2022**														
Malin 2020	Parents to babies born 23 to < 37 wks	3 months after discharge	PPQ ≥ 19	Parents *N* = 164, *n* = 41 (25%)	Multivariable logistic regression	**Factors **(3 months post discharge)	**OR**			**95%CI **				***P*** ** value**
						**Clinical illness indicator**	**3.10**			** 1.1, 9.0**				**0.040**
						**Parent perceives “sick”**	**3.80**			**1.2, 12.6**				**0.027**
						Interaction between clinical illness/parent perceives “sick”	0.70			0.1, 3.9				0.696
						Nurses perceives “sick”	1.00			0.3, 3.4				0.999
						Physicians perceives “sick”	1.70			0.6, 5.1				0.355
						History of mental health (yes=1)	0.90			0.4, 2.1				0.767
						Single parent	1.70			0.4, 8.0				0.510
Malin 2022		At 3 months after discharge	PPQ ≥ 19	Parents *N* = 245, *n* = 91 (36%)	T test comparing PTS positive vs PTS negative	**Factors **(3 months post discharge)	**PTS positive N (%)**			**PTS negative N (%)**				**χ2T‐test**
						GA								
						22−25 wks	13 (48)			14 (52)				0.16
						26−28 wks	14 (50)			14 (50)				
						29−31 wks	12 (28)			31 (72)				
						32−36 wks	29 (32)			66 (68)				
						≥37 wks	18 (36)			32 (64)				
						≤28 wks vs >28 wks	*OR 1.42, 95%CI 0.90, 2.24*							*P = 0.13*
						Palliative care consultation								
						Yes	5 (63)			3 (37)				0.10
						No	81 (34)			154 (66)				
						**Ventilator days**								
						0 days	26 (25)			77 (75)				<0.001
						1−7 days	28 (39)			43 (61)				
						8−30 days	9 (32)			19 (68)				
						**>30 days**	**23 (56)**			**18 (44)**				
						**Vasopressors**								
						**Yes**	** 30 (51) **			**29 (49)**				<0.001
						No	56 (30)			128 (70)				
						**Bronchopulmonary dysplasia**								
						None		54 (33)		109 (67)				0.10
						Mild		1 (17)		5 (83)				
						Moderate		8 (30)		19 (70)				
						**Severe**		**22 (51)**		**21 (49)**				
						Seizures		N (%)		N (%)				0.91
						Yes		2 (33)		4 (67)				
						No		84 (35)		153 (65)				
						Hypoxic ischemic encephalopathy		N (%)		N (%)				
						Yes		3 (60)		2 (40)				0.24
						No		83 (35)		155 (65)				
						**Length of NICU hospitalization in days**		**Mean (SD) 81.5 (69.9)**		**Mean (SD) 56.4 (57.4) **				**0.01**
						Parents’ age		Mean (SD) 29.9 (5.88)		Mean (SD) 29.7 (5.05)				0.84
						Ethnicity		N (%)		N (%)				
						Black or African American		13 (28)		33 (72)				
						White		70 (40)		107 (60)				0.13
						Asian		0		6 (100)				
						American Indian or Alaska Native		0		2 (100)				
						Other		3 (27)		8 (73)				
						Housing								
						Has housing		83 (35)		153 (65)				0.48
						Does not have housing		1 (25)		3 (75)				
						Prefer not to answer		2 (67)		1 (33)				
						Education								
						Not finished high school		7 (54)		6 (46)				0.61
						High school graduate		13 (30)		31 (70)				
						Some college of technical school		21 (37)		36 (63)				
						College or technical school graduate		34 (35)		64 (65)				
						Graduate school		11 (35)		20 (65)				
						Employment								
						Unemployed		18 (35		34 (65)				0.73
						Part‐time or temporary work		12 (34)		23( 66)				
						Full‐time		50 (37)		84 (63)				
						Unemployed		6 (32)		13 (68)				
						Prefer not to say		0		3 (100)				
						Lack of transportation								
						Yes		7 (64)		4 (36)				0.05
						No		79 (34)		152 (66)				
						**History of mental illness**								
						Yes		52 (50)		51 (50)				< 0.001
						No		30 (23)		101 (77)				
						Do not know		3 (37)		5 (63)				
						**Family history of mental illness**								
						Yes		47 (48)		51 (52)				0.001
						No		34 (26)		98 (74)				
						Do not know		5 (38) 9 62		9 (62)				
						**Uncertainty about infant’s health at NICU**		**Mean 71.6 (SD 17.2)**		**Mean 65.4 (SD 16.2)**				**< 0.001**
						**Uncertainty about infant’s health at 3 months**		**Mean 67.6 (SD 16.8)**		**60.4 (SD 16.2)**				**< 0.001**
**Misund 2013 & 2014** ^**a**^	Mothers to infants < 33 wks	2 weeks after hospitalization	IES ≥ 19	Mothers *N* = 29, *n* = 13 (44.8%)	Multivariable linear regression	**Factors **(2 wks after hospitalisation)		**B**		**95%CI**				***P*** ** value**
						**Preeclampsia**		**5.75**		**1.22, 10.27**				**0.015**
						**IVH**		**11.34**		**3.17, 19.50**				**0.008**
						**Mother's age**		**0.91**		**0.18, 1.64**				**0.016**
						**Planned caesarean section vs normal birth**		**-14.46**		**-27.67,−1.25**				**0.033**
**Moreyra 2021** ^**a**^	Mothers and fathers to NICU infants	14 days post NICU admission	PPQ ≥ 19	Mothers and fathers *N* = 150, *n* = 25 (17%)	Difference between the groups using t-test and correlation analysis	**Factors **(During NICU stay at 14 days post admission)				**T test **				**P **
						**Parents sex mothers vs fathers **				**6.53**				**< 0.0001**
						Ethnicity				NR				NS
						Correlation				**r**				**P**
						**Anxiety **				**0.79 **				** < 0.001**
**Naeem 2019**	Mothers and fathers to infants born 24-36 weeks	3-5 days after birth	Questionnaires for acute stress disorder (ASD) part of a clinical interview ≥ 56	Mothers *N* = 80, *n* = 26 (32.5%) fathers *N* = 80, *n* = 3 (4%)	Univariable logistic regression	**Factors **(3-5 days after birth)				**P value mothers**				***P*** ** value fathers**
						Father unemployment				0.22				0.22
						History of an accident during recent years for father				0.10				0.73
						Mother employment				0.82				0.46
						**History of an accident during recent years for mother**				**0.002**				0.50
						***Parent sex***				***OR 12.36, 95%CI 3.56, 42.91***				***<0.0001***
		1 month after the first assessment	PCL ≥ 30 and PPQ > 30	Parents *N *= 160 mothers *N*= 80, *n* = 63 (40%), fathers *N* = 80, *n* = 34 (21.5%)	Non paramedic test	**Factors **(1 month post first assessment)				***P*** ** value mothers**				***P*** ** value fathers**
						**Father’s unemployment**				**0.02**				0.23
						**History of an accident during recent years for father**				**0.02**				**0.01**
						**Mother employment**				**0.038**				0.46
						**History of an accident during recent years increase mothers**				**0.002**				0.09
						***Parent sex OR 5.01, 95%CI 2.50, 10.05***								
**Pace 2020**	Parents of infants born < 30 wks	At TEA, 12, 24 months,	PCL ≥ 30	Parents *N* = 105 (92 mothers and or 75 fathers) At TEA, 12, 24 months, PTSS *n* = 32 (36%), *n* = 12 (22%), *n* = 17 (18%) mothers & *n* = 26 (35%),* n* = 13 (25%), *n* = 14 (19%) fathers	Multivariable hierarchical logistic regression	**Factors **(12 and 24 months CCA)		**OR**		**95%CI **				***P***
						Medical risk		2.09		0.81, 5.38				0.13
						Multiple birth		2.18		0.80, 5.93				0.13
						Social risk		1.66		0.64, 4.30				0.30
						*Parent sex at 12 months*		*1.06 *		*0.56, 2.0.4*				*NS*
						*Parent sex at 24 months*		*0.97*		*0.44, 2.13*				
**Pisoni 2020** ^**a**^	Mothers to infants born < 24 wks	During NICU stay & 12 months infant corrected age	mPPQ, cut-off = NR	Mothers *N* = 29, during NICU *n* = 5 (17.25%), at 12 months 9 (31.05%)	Correlation	**Factors **(during NICU)				***r***				***P*** ** value**
						**Perinatal risk inventory (PERI)**				**0.611**				**< 0.05**
						Dyadic Synchrony Care Index				0.144				NS
						**Factors **(At 12 months)				***r***				***P*** ** value**
						Perinatal risk inventory (PERI)				0.098				NS
						Generalised Developmental Quotient (GQ)				-0.22				NS
						Dyadic Synchrony Care Index				0.009				NS
**Rodriguez 2020 **	Mothers of infants born < 32 wks	6 months after birth to > 36 months	Davidson Trauma Scale (DTS) - DSM-IV	Mothers *N* = 146, *n* = 64 (44%)	Mantel-Haenzel method	**Factors **(6 to > 36 months**)**			**OR**	**95%CI**				**P value**
						**GA ≤ 28 vs 29-31.6 wks**			***3.97***	***1.89, 8.33***				***0.0003***
						**BW < 1000 g vs 1000-1490 g**			***2.19***	***1.12, 4.27***				*** 0.02***
						Neonatal morbidity			*NR*	*NR*				*0.072*
						**Baby severe vs mild/moderate morbidity**			***2.26 ***	***1.12, 4.55***				***0.02***
						Maternal age ≤21-31 vs ≥32 years			*1.51 *	*0.76, 3.00*				*0.24*
						Children’s age 7-24 months vs 25 to > 36 months			*0.75 *	*00.37, 1.53*				*0.43*
						Length of stay NICU			NR					0.316
					Logistic regression	**Factors**			**OR**		**95%CI**			***P*** ** value**
						**lower level of education**			0.871		0.771, 0.984			0.026
**Salomè 2022**	Couples to NICU babies	During NICU, 1 year post NICU discharge	IES-R score ≥ 33	Parents *N* = 40, mothers *n *= 20 (55%), and 4 (20%) fathers	Multivariable regression	**Factors **(At 1 yr post discharge)		***R***	**B**		**t**			***P***
						Maternal Factors								
						**Maternal PSS:NICU total score**		**0 .37**	**0.612**		**3.28**			**< 0.01**
						**Maternal social functioning** **Subscale of Short form health survey (SF-36)**		**0.40**	**-0.62**		**-3.43**			**< 0 .01**
						Paternal Factors								
						**Maternal PSS:NICU total score**		**0.52**	**0.72**		**4.40**			**< 0.001**
						**Paternal self-rating depression**		**0.49**	**0.70**		**4.13**			**< 0.01**
**Sharp 2020**	Mothers to Term & preterm infants	1-4 months after birth	PCL-5 > 33	Mothers *N* =77, *n* = 18 (23.4%)	Linear regression	**Factors **(1-4 months)		**β**	**SE**		**95%CI**			***P***
						Time since birth		-0.01	0.08		-0.17, 0.16			0.937
						Duration of NICU stay		0.13	0.08		− 0.02, 0.29			0.093
						Prior trauma		2.37	1.41		− 0.45, 5.19			0.098
						**Parental Stressor Scale (PSS) total**		** 0.65**	**0.09**		**0.11, 1.18**			**0.020**
						Traumatic childbirth		2.44	4.58		-6.72, 11.60			0.596
**Shaw 2009**	Parents to term and preterm infants GA 27-41 wks	2-4 wks after NICU admission and at 4 months after birth	SARQ > 38	Baseline mothers *N* = 25, *n* = 14 (54.5%) vs fathers *n* = 13, (0%) At 4 months mothers *N* = 11, 6 (55%) at risk, PTSD 1 (9%) PTSD, fathers at risk N= 6, 4 (67%), PTSD 2 (33%)	Correlation	**Factors **(4 months after birth)			***r***					***P*** ** value**
						GA			0.20					NS
						BW			0.03					NS
						Apgar grade at 1 minute			0.29					NS
						Apgar grade at 10 minutes			0.22					NS
						Length of stay in hospital			-0.40					NS
						PSS: NICU: Infant behaviour and appearance Parental role-alteration **Sights and sounds** Staff relationships Total			0.09					NS
									0.35					NS
									**0.52**					**< 0.05**
									0.13					NS
									0.42					NS
						**Acute stress disorder (ASD)**			**0.54 **					**< 0.05**
						**Beck Depression Inventory (BDI) baseline**			**0.54**					**< 0.05**
						**BDI at follow-up**			**0.79**					**< 0.01**
						SCL90–R: Symptom Checklist, Revised: **Somatization** **Anxiety** **Depression** Interpersonal sensitivity **Obsessive-Compulsive Disorder** **Paranoid Ideation** Phobia Hostility Pychoticism **Global Severity Index**			0.41					NS
									**0.56**					**< 0.05**
									**0.60**					**< 0.05**
									**0.53**					**< 0.05**
									0.50					NS
									**0.62 **					**< 0.05**
									**0.70**					**< 0.01**
									0.46					NS
									0.40					NS
									**0.68**					**< 0.01**
						*Parent sex OR 0.60, 95%CI 0.08, 4.76*								
**Vinall 2018**	Mothers to infants born < 37 wks GA	At NICU discharge	PTSS checklist for DSM-5	Mothers = 36, n = 2 (6%)	Multivariable linear regression	**Factors **(NICU discharge)		**β**			**95%CI**			***P*** ** value**
						GA		0.30			−0.05, 0.64			NR
						Sex of baby		0.10			−0.16, 0.36			NR
						Illness severity – medical chart		0.13			−0.12, 0.37			NR
						**No. invasive procedures **		**0.40**			**0.003, 0.801**			NR
						Length of stay		0.05			−0.41, 0.51			NR
						Mother’s age		−0.159			−0.42, 0.09			NR
						**Mother’s years of education**		**−0.27**			** −0.52, −0.02**			NR
						Mothers memory of pain		0.21			−0.02, 0.44			NR
**Williams 2021**	Mothers to NICU infants born < 28 to > 37 wks	During first month of NICU stay	IES-R ≥ 33	Mothers = 119, n = 66 (55%)	Correlations	**Factors **(During NICU)					***r***			***P*** ** value**
						**Subjective infant health**					**0.60**			**< 0.01**
						**Chart infant health - SNAPPE II**					**0.35**			**< 0.01**
						**Apgar at 1 minute**					**−0.20**			**< 0.05**
						**Apgar at 5 min**					**−0.25**			**< 0.01**
						Worry about infant death					0.17			NS

*Abbreviations*: *95%CI* 95% Confidence Interval, *aOR* adjusted odd ratio, *BW* Birth weight, *CA* corrected age, *CES-D* Centre for Epidemiologic Studies Depression, *GA* Gestational age, *HADS* Hospital Anxiety and Depression Scale, *IES-R* Impact of Event Scale-Revised, *Italics* Data calculated, *mPPQ* Modified Perinatal Post-traumatic stress disorder Questionnaire, *NBRS* Neurobiologic Risk Score, *NICU* Neonatal Intensive Care Unit, *NR* Not reported, *NS* Not significant, *OR* Odd ratio, *MPI* Maudsley Personality Inventory, *PLC-5* Checklist for Diagnostic and Statistical Manual of Mental Disorders 5th edition, *PPQ* Perinatal Post-traumatic stress disorder Questionnaire, *PSS* Perceived Stress Scale, *r* Correlation coefficient, *Factors* Risk factors, *HMD* Severe neonatal morbidity referred to hyaline membrane disease, *IVH* grade 3 and 4, periventricular leukomalacia, *BPD* severe bronchopulmonary dysplasia, sepsis, meningitis, *NEC* necrotizing, enterocolitis, hyperbilirubinemia over the 90th percentile, *ROP* retinopathy of prematurity, symptomatic patent ductus arteriosus requiring surgery, *SNAPPE-II* Score for Neonatal Acute Physiology-Perinatal Extension-II, *TEA* Term Equivalent Age, *Wks* Weeks

^a^Studies included in both posttraumatic stress and anxiety: Garfield 2015 [27], Greene 2015 & 2019 [28, 29], Holditch-Davis 2009 [31], Lotterman 2018 [45], Misund 2013 & 2014 [36, 37], Moreyra 2021 [47], Pisoni 2020 [49]

**Table 3:** Mapping of posttraumatic (PTS) factors

Not statistically significant								FACTORS				Statistically significant			
Parent demographic factors															
Brunson 2021 [23] *n* = 50	Garfield 2015 [27] *n* = 113	Greene 2019 [29] *n* = 69	Kim 2015 [50] *n* = 120	Lefkowitz 2010 [34] *n* = 130	Malin 2022 [46] *n* = 245	Rodriguez 2020 [40] *n* = 146	Vinall 2018 [43] *n* = 36	age	Misund 2013 & 2014 [36, 37] *n* = 29						
				Brunson 2021 [23] *n* = 50	Garfield 2015 [27] *n* = 113	Kim 2015 [50] *n* = 120	Malin 2022 [46] *n* = 245	education	Clark 2021 [25] *n* = 67	Rodriguez 2020 [40] *n* = 146	Vinall 2018 [43] *n* = 36				
			Anchan 2021 [22] *n* = 106	Clark 2021 [25] *n* = 67	Lefkowitz 2010 [34] *n* = 130	Shaw 2009 [42] *n* = 38	Pace 2020 [39] *n* = 105	sex	Moreyra 2021 [47] *n* = 150	Naeem 2019 [38] *n* = 160					
					Lefkowitz 2010 [34] *n* = 130	Malin 2022 [46] *n* = 245	Moreyra 2021 [47] *n* = 150	ethnicity							
								parents ‘area deprivation	Greene 2019 [29] *n* = 69						
							Malin 2022 [46] *n* = 245	housing and access to transport							
								family income	Clark 2021 [25] *n* = 67						
							Malin 2022 [46] *n* = 245	employment status	Naeem 2019 [38] *n* = 160						
							Malin 2020 [46] *n* = 164	single parent							
							Pace2020 [39] *n* = 105	family social risk							
Pregnancy and birth factors															
							Brunson 2021 [23] *n* = 50	parity	Greene 2015 [28] *n* = 69	Kim 2015 [33] *n* = 120					
					Brunson 2021 [23] *n* = 50	Kim 2015 [50] *n* = 120	Pace 2020 [39] *n* = 105	multiple pregnancy							
							Brunson 2021 [23] *n* = 50	mode of birth	Misund 2013 & 2014 [36, 37] *n* = 29						
								preeclampsia	Misund 2013 & 2014 [36, 37] *n* = 29						
							Brunson 2021 [23] *n* = 50	threatened preterm labour							
							Brunson 2021 [23] *n* = 50	In vitro fertilization							
							Sharp 2021 [42] *n* = 77	traumatic childbirth							
Infant demographic factors															
			Anchan 2021 [22] *n* = 106	Kim 2015 [50] *n* = 120	Malin 2022 [46] *n* = 245	Shaw 2009 [42] *n* = 38	Vinall 2018 [43] *n* = 36	gestational age	Brunson 2021 [23] *n* = 50	Rodriguez 2020 [40] *n* = 146					
						Eutrope 2014 [26] *n* = 100	Rodriguez 2020 [40] *n* = 146	birth weight	Brunson 2021 [23] *n* = 50	Greene 2019 [29] *n* = 69	Shaw 2009 [42] *n* = 38				
							Williams 2021 [44] *n* = 119	Apgar score	Brunson 2021 [23] *n* = 50	Kim 2015 [50] *n* = 120	Shaw 2009 [42] *n* = 38				
						Brunson 2021 [23] *n* = 50	Vinall 2018 [43] *n* = 36	sex							
							Rodriguez 2020 [40] *n* = 146	age							
Infant health and care factors															
			Brunson 2021 [23] *n* = 50	Clark 2021 [25] *n* = 67	Garfield 2015 [27] *n* = 113	Lefkowitz 2010 [34] *n* = 130	Malin 2020 [46] *n* = 164	clinicians’ perception of infant health	Eutrope 2014 [26] *n* = 100	Pisoni 2020 [49] *n* = 29	Rodriguez 2020 [40] *n* = 146	Williams 2021 [44] *n* = 119			
								parents’ perception of infant health	Anchan 2021 [22] *n* = 106	Clark 2021 [25] *n* = 67	Lotterman 2018 [45] *n* = 91	Malin 2022 [46] *n* = 245	Williams 2021 [44] *n* = 119		
							Lotterman 2018 [45] *n* = 91	mother-infant contact							
							Lotterman 2018 [45] *n* = 91	number of NNU visits							
							Pisoni 2020 [49] *n* = 29	mother-infant relationship							
							Lotterman 2018 [45] *n* = 91	mother-nurse relationships							
				Rodriguez 2020 [40] *n* = 146	Sharp 2021 [41] *n* = 77	Shaw 2009 [42] *n* = 38	Vinall 2018 [43] *n* = 36	length of stay in NNU	Lefkowitz 2010 [34] *n* = 130	Lotterman 2018 [45] *n* = 91					
								intraventricular haemorrhage	Misund 2013 & 2014 [36, 37] *n* = 29						
								ventilation support	Malin 2022 [46] *n* = 245						
								severe bronchopulmonary dysplasia	Malin 2022 [46] *n* = 245						
								vasopressors support	Malin 2022 [46] *n* = 245						
							Clark 2021 [25] *n* = 67	number of invasive procedures	Vinall 2018 [45] *n* = 36						
							Malin 2022 [46] *n* = 245	hypoxic ischemic encephalopathy							
							Malin 2022 [46] *n* = 245	palliative care consultation							
							Malin 2022 [46] *n* = 245	seizures							
							Pisoni 2020 [49] *n* = 29	infant’s general development							
							Kim 2015 [50] *n* = 120	rehospitalisation or emergency visits							
Parental history of mental health and trauma factors															
								history of mental health problems	Anchan 2021 [22] *n* = 106	Lotterman 2018 [45] *n* = 91	Malin 2022 [46] *n* = 245				
								family history of depression / mental health	Lefkowitz 2010 [34] *n* = 130	Malin 2022 [46] *n* = 245					
							Sharp 2021 [41] *n* = 77	previous traumatic events	Greene 2015 2019 [28, 29] *n* = 69	Naeem 2019 [38] *n* = 160					
							Sharp 2021 [41] *n* = 77	traumatic childbirth							
Parental postnatal mental health factors															
								postnatal depression	Anchan 2021 [22] *n* = 106	Brunson 2021 [23] *n* = 50	Eutrope 2014 [26] *n* = 100	Holditch-Davis 2009 [31] *n* = 117	Jubinville 2012 [32] *n* = 40	Shaw 2009 [42] *n* = 38	Salomè 2022 [48] *N* = 40
								postnatal anxiety	Brunson 2021 [23] *n* = 50	Eutrope 2014 [26] *n* = 100	Garfield 2015 [27] *n* = 113	Holditch-Davis 2009 [31] *n* = 117	Moreyra 2021 [47] *n* = 150		
							Lotterman 2018 [45] *n* = 91	early PTS symptoms	Anchan 2021 [22] *n* = 106	Lefkowitz 2010 [34] *n* = 130	Shaw 2009 [42] *n* = 38				
								other mental health symptoms	Chang 2016 [24] *n* = 102	Eutrope 2014 [26] *n* = 100	Shaw 2009 [42] *n* = 38				
						Parent stress, coping and support factors									
							Shaw 2009 [42] *n* = 38	parental stressor scale total score	Sharp 2021 [41] *n* = 77	Salomè 2022 [48] *N* = 40					
							Shaw 2009 [42] *n* = 38	stress related to infant’s appearance	Holditch-Davis [31] 2009 *n* = 117						
								stress related to NNU sights and sounds	Shaw 2009 [42] *n* = 38						
						Anchan 2021 [22] *n* = 106	Shaw 2009 [42] *n* = 38	stress related to role alteration	Holditch-Davis [31] 2009 *n* = 117						
							Shaw 2009 [42] *n* = 38	stress relating to staff relationships							
								concurrent stressors	Lefkowitz 2010 [34] *n* = 130						
							Lotterman 2018 [45] *n* = 91	forward-focused coping style							
								maternal optimism	Lotterman 2018 [45] *n* = 91						
							Williams 2021 [44] *n* = 119	worry about infant’s death							
								social support	Eutrope 2014 [26] *n* = 100	Salomè 2022 [48] *n* = 40					
							Brunson 2021 [23] *n* = 50	psychological support							
						Other factors									
							Anchan 2021 [22] *n* = 106	military geographic separation							
							Anchan 2021 [22] *n* = 106	active military service							
								spiritual activity	Hawthorne 2016 [30] *n* = 165						
							Hawthorne 2016 [30] *n* = 165	religious activities							

The association between parental age and PTS symptoms was explored in nine studies, published in ten records [23, 27, 29, 33, 34, 36, 37, 40, 43, 46]. Older mothers had significantly higher PTS scores at two weeks post NNU admission in one study of only 29 mothers, reported in two records [36, 37]. In the remaining studies there was no significant association between parental age and PTS symptoms. Seven studies [23, 25, 27, 33, 40, 43, 46] explored the association between parental education and PTS symptoms. Lower education was associated with more PTS symptoms in three studies [25, 40, 43] and consistent with this finding, one study [43] found mothers with more years of education had fewer PTS symptoms at discharge. Similarly, in another study [40], mothers who had a lower education level accounted for significantly more cases of PTS at 6–36 months after birth. Additionally, among bereaved mothers [25], higher education level was associated with fewer PTS symptoms even three to five years after the baby’s death. The remaining four studies found no association between parental education and PTS symptoms. The association between sex of parent and PTS symptoms was explored in seven studies [22, 25, 34, 38, 39, 42, 47]. Three studies [34, 38, 39] provided data at multiple time points. Two studies [38, 47] found PTS symptoms were significantly more prevalent in mothers than fathers while their babies were still in NNU and a month later [38]. Evidence from the remaining five studies showed no association between sex of parent and PTS symptoms. Three studies [34, 46, 47] explored the association between parental ethnicity and PTS symptoms, and none found any association during NNU stay [34, 47] or at three months post NNU discharge [46]. However, in one of the studies [34], only 28% of participants were from minority backgrounds. The association between parents’ area deprivation and PTS was explored in one study [29], and mothers residing in poorer neighbourhoods had lower PTS scores at birth than those residing in more privileged neighbourhoods, but this association disappeared at one year. Housing and access to transport were not associated with PTS symptoms at three months post NNU discharge in one study [46]. In bereaved parents [25], a lower family income for fathers, but not for mothers, was significantly associated with more PTS symptoms at three months to five years after the baby’s death. Two studies [38, 46] explored the association between employment status and PTS symptoms. One study [46] found employment status was not associated with PTS symptoms after birth, yet the other study [38] found PTS symptoms were significantly greater among employed mothers and mothers with unemployed partners one month after the birth [38]. One study [35] found no significant association between being a single parent and PTS symptoms three months after NNU discharge. One study [39] explored family social risk, a composite of family structure, education, occupation, employment, language spoken and maternal age, and found no association with PTS symptoms in parents of very preterm infants at 12 and 24 months corrected age.

- 2) Pregnancy and birth factors (Seven factors: parity, multiple pregnancy, mode of birth, pre-eclampsia, threatened preterm labour, in-vitro fertilisation, traumatic childbirth)

Three studies [23, 28, 33] explored the association between parity and PTS symptoms. Two of the studies [28] found primiparity was a significant risk factor for elevated PTS symptoms during NNU [25] and at one year corrected age [33] and the third study [23] found no significant association between parity and PTS symptoms 18 months after birth. Multiple pregnancy was explored in three studies [23, 33, 39] and giving birth to twins was not associated with PTS symptoms in any study assessed at one year or later. The association between mode of birth and PTS symptoms was explored in two studies, reported in three records [23, 36, 37]. One study, reported in two records (2013, 2014), found planned caesarean section compared to normal birth was associated with lower PTS symptom scores at two weeks post NNU admission. However the other study [23] found caesarean section (planned and unplanned) was not significantly associated with PTS symptoms at 18 months, yet there were more caesarean sections among the group of mothers who experienced PTS symptoms during the study. Seventy-five percent required a caesarean section compared to 47.4% in the group with no significant PTS symptoms. Preeclampsia was significantly associated with higher PTS scores at two weeks post NNU admission in one study, reported in two records [36, 37]. A history of threatened preterm labour was explored in one study [23] and was not associated with PTS symptoms at 18 months after birth. In vitro fertilization [23] and traumatic childbirth [41] were each explored in one study and were not found to be associated with PTS symptoms at one to four months and 12 months after birth, respectively.

- 3) Infant demographic factors (Five factors: gestational age, birth weight, Apgar score, sex of infant, age at infant)

Seven studies [22, 23, 33, 40, 42, 43, 46] explored the association between gestational age (GA) and PTS symptoms. Only two studies [23, 40] found a significant association between GA and PTS symptoms. One study [23], where GA ≤ 32 weeks was an inclusion criterion, found that infants born to mothers with elevated PTS scores 18 months after birth had a lower GA age by almost one week, and one study [40] found a significantly higher frequency of infants born ≤ 28 weeks gestation among mothers with more PTS symptoms 6 to > 36 months after birth. The association between birth weight (BW) and PTS symptoms was explored in five studies, [23, 26, 29, 40, 42]. Increased infant’s BW was significantly correlated with lower PTS symptoms during NNU stay [26] and lower PTS score at birth, but not at 12 months later [29]. PTS symptoms were more prevalent at six to > 36 months among mothers to a very low BW (< 1000 g) infant [40]. Two other studies [23, 42] reported no significant association between BW and PTS symptoms at four and 18 months after birth, respectively. The association between Apgar score and PTS symptoms was explored in four studies [23, 33, 42, 44] and only one study [44] found Apgar scores (1 min, 5 min) and PTS symptoms were negatively correlated during NNU admission. Two studies explored sex of infant and PTS symptoms and found no association at NNU discharge [43] or 18 months after birth [23]. One study explored age of infant and PTS symptoms at 7–24 months vs 25 to > 36 months and found no association [40].

- 4) Infant health and care factors (17 factors: clinicians’ perception of infant health, parents’ perception of infant health, mother-infant contact, length of NNU stay, mother-infant relationship, mother-nurse relationships, hypoxic ischemic encephalopathy (HIE), ventilation, severe bronchopulmonary dysplasia, vasopressor, hypoxic ischemic encephalopathy (HIE), palliative care consultation, seizures, invasive procedures, number of medical interventions, infant general development, re-hospitalisation and emergency visit.

Nine studies reported on clinicians’ perception of infant health [23, 25–27, 34, 35, 40, 44, 49]. Three studies [23, 26, 49] used the Perinatal Risk Inventory (PERI) scale [51] to assess clinicians’ perceived risk of adverse infant outcomes. One study [26] found a significant correlation between PRI score and parental PTS symptoms during NNU, one study found a significant correlation during NNU admission but not 12 months later [49], and one study found no association at 18 months corrected age [23]. One study used the Neonatal Acute Physiology-Perinatal Extension- II (SNAPPE-II) [44], a tool for predicting outcomes in critically ill newborns, and found a significant correlation between SNAPPE-II scores and PTS symptoms during NNU admission. Four studies [25, 34, 35, 40] used non-standardised clinical indicators to assess clinicians’ perception of the baby’s health but only one study reported a significant association. In one study [40], severe neonatal morbidity was significantly more common among mothers with elevated PTS score 6—> 36 months after birth. However, the Neurobiologic Risk Score (NBRS) [52] which assesses baby’s neurological insults was not significantly correlated with PTS scores three months after birth [27].

The association between parents’ perception of infant health and PTS symptoms was assessed in five studies, published in six records [22, 25, 35, 44–46], and all studies reported a significant association. Parents who appraised their infant’s health as “sick/severe” were almost four times more likely to report PTS symptoms in two studies, one at 1–2 months [22] and one at three months post NNU discharge [35]. Also in [46], parents’ uncertainty about infant’s health was significantly associated with higher PTS scores during NNU and at three months post discharge. Among parents of deceased babies [25], mothers’ perception of infants’ symptoms and fathers’ perception of infants’ suffering were associated with increased PTS scores even three to five years following infant death. One study found a significant correlation between subjective infant health and more PTS symptoms during NICU admission [44] and one study found that a higher number of health problems reported by the mother was associated with higher PTS scores six months after birth [45]. Neither mother-infant contact (verbal and physical contact rated on a five-point Likert scale) while in NNU nor the number of NNU visits per week were associated with PTS symptoms at six months [45]. Additionally, mother-infant relationship assessed by CARE-index [53], which measures the interaction patterns between infants and carers, was not associated with more PTS symptoms during NNU stay or at 12 months corrected age [49]. One study explored mother-nurse relationships at six months [ref],based on nurses rating mothers’ understanding of explanations relating to infants’ care and health (,and found no association with PTS scores.

Length of stay in NNU was explored in seven studies [34, 40–43, 45, 46] and only one study [43] adjusted for GA. Three studies [34, 45, 46] found significant, albeit contradictory, associations between length of NNU stay and PTS symptoms. One study [34] found longer length of stay was correlated with lower PTS scores during NNU admission and two studies [45, 46] found longer length of stay was associated with higher PTS scores at three months [46] and six months [45]. Low grade intraventricular haemorrhage (IVH) was significantly associated with higher PTS scores 2 weeks after NNU admission in one small study, reported in two records [36, 37]. Requiring ventilation for > 30 days, severe bronchopulmonary dysplasia (BPD) and vasopressors support were all more prevalent among parents who reported PTS at three months post NNU discharge [46] in one study. Parents of infants exposed to a greater number of invasive procedures had significantly more PTS symptoms during NNU in one study [43] which adjusted for GA. Conversely, another study [25] found number of medical interventions was not significantly associated with PTS symptoms 3 months to 5 years after infant death. Hypoxic ischemic encephalopathy (HIE), palliative care consultation and seizures were not associated with PTS scores in one study [46]. One study explored infant’s general development [49] and one study explored rehospitalisation or emergency visits [33]; neither were found to be significantly associated with PTS symptoms.

- 5) Parental history of mental health/trauma factors (Four factors: parental history of mental health problems, family history of mental health problems, previous traumatic events, traumatic childbirth).

Three studies reported on parental history of mental health problems [22, 45, 46] and all found significant associations. One study [22] found a significant association with a positive screening of PTS two weeks after birth, one study found an association during NNU [45] and at three months post NNU discharge [46]. One study found [45] previous mental health problems in addition to low mother-infant contact (physical or verbal) was significantly associated with higher PTS scores during NICU admission. Two studies [34, 46] reported a significant association between family history of depression/mental health problems and PTS symptoms during NNU admission [34] and at three months post discharge [46]. Previous traumatic events (physical or psychological e.g. car accident, unexpected death of loved ones and sexual assaults) were assessed in three studies, published in four records [28, 29, 38, 41] with mixed results. One study, published in two records [28, 29], found exposure to previous traumatic events was associated with increased PTS scores at birth and before NNU discharge, but not at one year. One study [38] found that a history of traumatic events, during recent years was not associated with PTS symptoms three to five days after birth, but was associated with PTS among mothers at a later assessment point around one month after birth. Finally, one study [41] found that prior trauma exposure was not associated with a significant increase in PTS scores one to four months after birth. PTS symptoms were higher among women who had a traumatic childbirth compared with those who did not, but no significant association was found in the regression analysis [41].

- 6) Parental postnatal mental health factors (Four factors: postnatal depression, postnatal anxiety, early PTS symptoms, other mental health problems)

The association between postnatal depression and PTS was explored in seven studies [22, 23, 26, 31, 32, 42, 48]. The timing of the assessment varied across the studies: during NNU admission [26, 31, 32, 42], at discharge [22, 23, 26, 32], four months after birth [42] or at one year post discharge among fathers [48]. All studies reported a significant association between postnatal depression and PTS symptoms irrespective of when the measurement was taken.

The association between postnatal anxiety and PTS was explored in five studies [23, 26, 27, 31, 47]. All reported a significant correlation between anxiety scores and PTS scores during NNU admission [26, 31, 47], at three months after birth [27] and at 18 months after birth [23]. In four studies [22, 34, 42, 45], the association between early PTS symptoms and PTS symptoms later in the postnatal period was explored. PTS symptoms around the time of NNU admission was a significant risk factor for an increase in PTS symptoms at one month post discharge [34], at 1–2 months post discharge [22] and 4 months after birth [42]. However, PTS scores during NNU stay were not significantly associated with PTS scores at 6 months [45].

Other mental health symptoms were explored in three studies [24, 26, 42]. The combination of anxiety and depression assessed by the Hospital Anxiety and Depression Scale (HADS) was correlated with PTS around birth and before NNU discharge in one study [26]. The combination of high depression and neuroticism scores was a significant risk factor for PTS at six to 48 months after birth in one study [24]. Finally, general psychiatric symptomatology assessed by the Symptom Checklist-90–Revised (SCL-90–R) was significantly correlated with PTS scores in another study [42].

- 7) Parent stress, coping and support factors (11 factors: Parental Stressor Scale total score, stress related to infant’s appearance, stress related to sights and sounds, stress related to parental role alteration, stress related to parent-staff relationships, concurrent stressors, forward-focused coping style, maternal optimism, worry about infant’s death, social support, psychological support).

Parental stress was measured using the Parental Stressor Scale: Neonatal Intensive Care Unit (PSS: NICU) [54] in four studies [31, 41, 42, 48]. The PSS: NICU assesses different domains of stress including sights and sounds, infant appearance and parental role in addition to providing a total parental stress score. Examples of stress related to alteration in the parental role are feeling helpless, being separated from the infant and unable to provide care. Three studies [41, 42, 48] reported PSS total scores; two studies found a significant association with an increase in PTS scores at one to four months after birth [41] or at one year after NNU discharge [48]. However, another study found no association was reported at four months after birth [42]. Two studies [31, 42] reported on parental stress related to infant’s appearance and this was associated with higher PTS scores in one study [31] when PTS was assessed on admission to NNU; another study found no significant association when PTS was measured at 4 months [42]. One study [42] found PTS scores were significantly correlated with the stress related to sights and sounds in the NNU at four months post birth. Stress related to role alteration during NNU admission was evaluated in three studies [22, 31, 42], only one of which found higher stress relating to role alteration correlated with higher PTS scores during NNU admission [31]. One study [42] found that stress relating to relationships with staff during NNU was not significantly correlated with PTS scores.

The number of concurrent stressors was found to be a significant risk factor during NNU admission in one study [34]. Concurrent stressors included social stressors, such as change in relationship status, living arrangements, or job status, and stressors such as loss, personal or family health concerns, experience of a traumatic event or legal problems.

Coping styles and flexibility after a traumatic event were assessed in one study [45] using the perceived ability to cope with trauma scale [55], which has two subscales: forward focus and trauma focus. A forward-focused coping style was not associated with PTS symptoms [45], whereas maternal optimism about the infant’s recovery while in NNU significantly reduced the likelihood of reporting PTS at 6 months [45]. One study explored worry about infant’s death and found it was not associated with PTS during NNU admission [44]. Two studies looked at the association between social support and PTS symptoms [26, 48]. Satisfaction with social support was associated with lower PTS symptoms in one study [26] and maternal social functioning was associated with a reduction in PTS at one year after NNU discharge in another study [48]. Mothers scoring above and below the cut-off point on the modified perinatal PTSD questionnaire were not found to differ in the psychological support they received in a further study [23].

- 8) Other factors (Four factors: geographic separation, active duty, spiritual activities, religious activities)

In a study including military personnel [22], geographic separation (defined as a combat zone deployment of any duration and a separation from family for more than four months at any time, or for more than one month during the most recent pregnancy) and active military service of either parent was not significantly associated with PTS at any time point. In a study of bereaved parents [30], spiritual activity without adopting a specific religion was associated with lower PTS scores among mothers but not fathers, whereas using religious activities as a coping mechanism was not associated with a significant reduction in PTS scores in either parent.

## Anxiety

### Description of included studies

Table 4 presents the 31 included studies, published in 33 records [27–29, 31, 36, 37, 45, 49, 56–74], for anxiety (including 7 studies for both anxiety and PTS).

**Table 4:** Characteristics of anxiety included studies

Study ID, country	Study design, setting, study period, type of neonatal care, length of stay	Study objective	Study inclusion criteria	Study exclusion criteria	Parents’ characteristics	Babies’ characteristics
Blanc 2021 [56], France	Prospective national population-based cohort, 268 neonatology departments, March-December 2011, length of stay = median 29 (Q1, 16 – Q3, 60) days, NICU level = NR	To evaluate if caesarean delivery < 26 wks GA is associated with depression and anxiety in mothers compared with 26 and 34 wks deliveries	Mothers to live preterm infants 22–34 weeks who enrolled in the EPIPAGE-2 study	Mothers who had multiple births (twins or more) among whom at least one infant died	*N* = 2270 mothers Age = 29.9 ± 5.4 yrs, Parity—nullips = 1247 (56%) LWP = 2001 (92%) SES—employed = 1479 (70.5%) Ethnicity, education = NR	*N* = 2270 ( 1830 singleton and 440 multiple), GA = 22–34 wks, BW = 1,761.4g ± 527g
Bonacquisti 2020 [57], USA	Prospective cohort, 3 centres, October 2014-May 2016, NICU level & length of stay = NR	To identify maternal psychological responses to infants’ (NICU) admission, the relationship between psychological symptoms and maternal-infant attachment, evaluate change in psychological symptoms over time	Mothers > 18 years old, of NICU infants within one week to one year postpartum	Fathers excluded	*N* = 127 mothers Age = mean 29.68 yrs Parity—nullips = 102 (80%) LWP = 69 (54.3%) SES—employed = 87 (69%) Education—university and above = 47 (37%) Ethnicity = White 66 (52.0%), other 61 (48%)	N, GA, BW = NR
Buchi 2007 [58], Switzerland	Cross sectional, 1 centre, January 1998-December 2002, NICU level & length of stay = NR	To assess grief and post-traumatic growth in parents 2–6 years after the death of extremely preterm, to evaluate bereavement	Parents to deceased babies born at 24–26 weeks’ gestation during the study period	Insufficient command of German to complete questionnaire	*N* = 54 parents (27 mothers + 27 fathers) Age—mothers = 34.7 ± 5.1 yrs Age—fathers = 38.9 ± 8.6 yers Parity—nullips = 11 (20%) LWP = all Education—university degree = parents 7 (13%) SES & ethnicity = NR	*N* = 40 (12 twins, 3 triplets), BW = NR, GA = 25.2 ± 0.9 wks
Cajiao-Nieto 2021 [59], Spain	Prospective cohort, 1 centre, January 2016-April 2017, length of stay = mean 31.2 days, NICU level = NR	To compare anxiety and depression symptoms between fathers of babies admitted to NICU and fathers of healthy full-term infants (not admitted to NICU)	Fathers of babies in NICU ≥ weeks, able to speak and write in Spanish	Death of the newborn or one of the infants in multiple births, being transferred to another hospital	*N* = 51 fathers Age—30–40 yrs = 36 (60.8%) Age > 40 yrs = 15 (29.4%) Parity—first-time fathers = 36 (70.2%) LWP = 32 (62.7%) > 10 yrs SES—employed = 49 (96.1%) Education—professional and above = 39 (76.5%) Ethnicity = NR	*N* = 69, GA—32 to 36 wks = 44 (63.8%), BW - 1000 -2000 g = 44 (63.9%)
Cakmak 2018 [60], Turkey	Cross sectional, 2 centres, July 2013-June 2015, level 1 and 2 NICU, length of stay = mean 8.43 ± SD 11.27 days, range = 1–85 days	To examine the correlation between mothers’ participation in NICU infant care, their anxiety and problem-solving skill levels in caregiving	Mother of infants 0–2 months in the NICU, > elementary school graduates, babies > 24 h in NICU, Speak Turkish, able to participate in baby care	Babies having contagious infection prevents mother from entering the NICU, mothers with physical disability/ psychiatric condition	*N* = 340 mothers Age = 27.7 ± 5.6 yrs Parity—nullips = 128 (37.6%) LWP—living in a nuclear family 286 (84.1%) SES—income < expenditure = 112 (32.9%) Education—university 55 (16.2%) Ethnicity = NR	*N* = 340, GA - 25–36 weeks = 140 (41.2%), 37 to 42 weeks = 200 (58.8%), BW = mean 2,700.29 ± SD 861.06 g
Carvalho 2008 [61], Brazil	Prospective cohort, 1 centre, 2001–2003, NICU level III, length of stay = mean 31 ± SD 24.67 days	To assess/compare anxiety and depression symptoms in mothers of preterm during NICU and afterwards and child’s development at 12 months of CCA	Mothers of NICU babies < 37 wks GA and BW ≤ 1,500 g	Psychiatric history, human immunodeficiency virus (HIV), maternal hospitalisation in intensive care	*N* = 36 mothers Age = mean 24.56 ± SD 6.81 yrs Parity – nullips = 21 (58%) LWP = 27 (75%) Education—high school = 10 (28%) SES & ethnicity = NR	*N* = 36, GA = mean 30.44 ± SD 2.26 wks, BW = mean 1,058 ± SD 241.98 g
Dantas 2012 [62], Brazil	Cross-sectional, 2 centres, April–May 2011, length of stay = mean 5 ± SD 6 days, NICU level = NR	To identify the prevalence of symptoms of anxiety and depression in mothers of hospitalized premature infants	Mothers of preterm infants < 37 wks, admitted to NICU > 24 h, age ≥ 18 yrs	Mothers to newborns who died, or with congenital anomaly, drug user, HIV + and mental health illness	*N* = 70 mothers Age = mean 26.50, range 18 – 42 yrs LWP = 55 (78.6%) SES—1 salary = 17 (24.3%) SES – occupation = 33 (47.1%) Education, parity, ethnicity = NR	*N* = NR, GA = mean 31.55 wks, range 26 to 37 wks, BW = mean 1,494 g
Damanabad 2019 [63], Iran	Cross sectional, 1 centre, January 2016—May 2016, NICU, length of stay = NR	To assess anxiety of mothers to NICU babies and the characteristics associated with anxiety	Mothers to NICU preterm babies 30–36 wks	For mothers severe obstetric complications and transferred to another hospital For babies congenital abnormality, baby died in the first 24 h	*N* = 100 mothers Age = mean 29.98 yrs Parity—nullips = 49 (49%) Education—university = 17 (17%) LWP, SES & ethnicity = NR	*N* = 100, GA ≤ 30 wks = 33 (33%) GA 31–34 wks = 30 (30%) GA > 34 wks = 37 (37%) BW = mean 1,851.67 ± SD 573.42 g
Das 2021 [64], USA	Prospective cohort, 1 centre, length of stay = range 14–69 days, study period & NICU level = NR	To determine if history of depression would increase risk of both postpartum depression and other stress-related disorders among NICU mothers	Mothers of newborns in NICU 7–29 days	Not giving informed consent	*N* = 96 mothers, 36 with mental health history, 60 no mental health Age = range 22–33 yrs SES—government insurance = 86 90% Ethnicity = white 36 (38%) Parity, education & LWP = NR	*N* = 99, GA = range 29–39 wks, BW = range 1,285–3112 g
Dickinson 2022 [75], Australia	Prospective cohort, 1 centre, January 2016—November 2016, NICU level 6 tertiary care, length of stay = NR	To estimate prevalence of psychological symptoms among mothers and fathers of babies admitted to NICU within 2 weeks after birth	Parents of preterm infants ≤ 37 weeks' gestational age	Not speaking English, aged < 18 years, infant unlikely to survive/ died within the 1st wk/admitted to NICU within 72 h after birth	*N* = 114 mothers & fathers, N mothers = 69, *N* = 45 fathers Age—combined = mean 30.58 ± 8.9 yrs Parity—combined = first child = 47 (41.2%) LWP – combined = 97 (87.4%) Education- combined > 12 yrs = 61 (53.5%) SES—combined income < $50 000 = 33 (28.9%), rural residents (combined) = 41 (36.0%) Ethnicity—combined) = indigenous = 21 (18.4%), white = 81 (71.1%), others = 12 (10.5%)	*N* = 79, GA mean = 30.12 ± SD 3.687 wks, BW = mean 1,521.82 ± SD 712.9 g
Feeley 2007 [65], Canada	Prospective cohort, 2 centres, length of stay = range 26–129 days, period & NICU level = NR	To compare mothers and fathers psychosocial adjustment and interaction with their infants at 3 and 9 months	Biological parents of babies BW < 1500 g and GA < 37 weeks, married or cohabiting, able to read English/French	Parents to babies with congenital anomaly or neurological disability	*N* = 61 couples Age—mothers = mean 33.0 ± SD 5.3 yrs Age—fathers = mean 34.5 ± SD 5 yrs Parity—first time parents = 38 (62%) LWP = all Educations—mothers = mean 14.1 ± SD 3,1 yrs Education—fathers = mean 14.3 ± SD 2.8 yrs Ethnicity & SES = NR	*N* = 61, GA < 37 wks, BW < 1500 g
Fontoura 2018 [66], Brazil	Cross-sectional, 3 centres, May 2014-April 2015, neonatal units, length of stay = NR	To compare the anxiety level of mothers of newborns with congenital anomalies who were diagnosed anenatally and postnatally	Mothers who were physically and mentally able to participate	Mothers with psychiatric conditions, HIV + impaired hearing, antenatal complications, using psychotropic medications, discharged before the diagnosis of congenital malformation at birth	*N* = 115 mothers Age 19–29 yrs = 58 (50%) Parity—nullips = 47 (41%) LWP = 68 (59%) Education - 6 to 10 years = 60 (52%) Ethnicity—declared to be browns = 107 (93%) SES = NR	*N* = 117, BW & GA = NR
Garfield 2015^a^, [27] USA	Cross-sectional, 2 centres, length of stays = mean 93.1 ± SD 48.49, range 31–211 days, period & NICU level = NR	To identify risk factors among urban, low-income mothers, for screening and referral	Mothers of VLBW < 1500 g and preterm < 37 wks, English speaking, no current mental health diagnosis, infants clinically stable, no congenital neurological problems or symptoms of substance abuse	Mothers < 18 yrs old, ongoing critical illness (HIV, seizure), major depression, psychosis, bipolar disease, mothers to infants receiving mechanical ventilation	*N* = 113 mothers Age = mean 24.7 ± SD 5.17 yrs LWP = 59 (52.3%) SES—received public aid = 44 (39%), uninsured = 45 (40%) Education—high school graduates = 49 (43%) Ethnicity = African American 92 (81%) Parity = NR	*N* = NR, GA < 37 wks, BW = mean 1,073 ± SD 342 g
Gennaro 1988 [67], USA	Prospective cohort, 1 centre, over 7 months, NICU, level & length of stay = NR	To compare anxiety and depression between preterm and term mothers at 1 and 6 weeks	Mothers to preterm < 37 wks and BW 1000–2500 g	No anomaly babies	*N* = 41 mothers Age = mean 23 ± SD 5.2 yrs SES—low-middle class = all Parity, LWP, ethnicity, & education = NR	*N* = NR, GA < 37 wks, BW = mean 1,618 g
Greene 2015 & 2019^a^, [28, 29] USA	Prospective cohort, 1 urban centre, 2011–2012, NICU level IV, length of stay = mean 91 ± SD 37 days	To analyse change of depression, anxiety and perinatal-specific PTS across very low birth infants’ first year of life, to identify predictors of changes over time	English-speaking mothers > 18 years, babies likely to survive and VLBW < 1500g	NR	*N* = 69 mothers at about 1 month post birth, *N* = 64 at about 2 wks before discharge Age = mean 27 ± SD 6 yrs Parity – nullips = 23 (34%) LWP = 32 (51%) Education—highest grade = mean 13.4 ± SD 2.4 yrs Ethnicity = Black 38 (54%), Non-Hispanic white 18 (26%), Hispanic 12 (17%), Asian 1 SES = NR	*N* = 69, GA = mean 27.5 ± SD 2 wks, range = 23.2 to 32.3 wks, BW = mean 957 ± SD 243 g
Holditch-Davis 2009^a^, [31] USA	Prospective cohort, 2 centres, NICU level, study period = NR	To examine inter-relationships among stress due to infant appearance and behaviour in the NICU exhibited by African American mothers of preterm infants	African American biological mothers of preterm infants < 1500 g at birth or requiring mechanical ventilation. Mothers were recruited when their infants were no longer critically ill	Infants with congenital, symptomatic from substance exposure, hospitalized > 2 months post-term, or triplets or part of a higher order multiples set; mothers with no custody, follow-up for 2 years unlikely, HIV + , < 15 yrs, critically ill, not speak English, mental health problems	*N* = 177 mothers Age = mean 25.9 ± SD 6.5 LWP = 70 (6.1%) Education = mean 12.6 SD ± 1.8 yrs SES—public assistance = 61 (52.8%) Ethnicity = all African American Parity = NR	*N* = NR, GA = mean 28.3 ± SD 2.9 wks, BW = mean 1,107 ± SD 394 g
Khemakhem 2020 [73], Tunisia	Cross-sectional, 1 centre, March–May 2017, length of stay = median 6 days, interquartile range (IQR) = 3–16 days	To examine the interactions between mothers and premature babies in NCU and assess mothers’ psychological state	Mothers to babies born prematurely and admitted to NICU	NR	*N* = 10 Age = median 31, (IQR) 25 and 37 yrs, Parity—nullips = 2 LWP = All Education—university = 4 Ethnicity = all Arabs SES = NR	*N* = 11, BW = median 1,750, IQR 1,480–2,100 g, GA 28–37 wks
Kong 2013 [74], China	Cross sectional, 1 centre, January–September 2011, neonatal care paediatric department, length of stay > 24 h	To investigate parents’ mental health of hospitalised neonates and their characteristics, to measure the stress levels and social support	Parental age ≥ 18 years, ability to read and write, neonates stayed in hospital > 24 h	Serious physical or mental health condition	*N* = 600 mothers & fathers, mothers *N* = 200, fathers *N* = 400 Age – mothers = mean 28.53 ± SD 4.06 yrs Age – fathers = mean 30.76 ± SD 4.60 yrs LWP – average yrs- mothers = mean 3.17 ± SD 2.78 LWP – average yrs- fathers = mean 3.30 ± SD 3.13 yrs Education – college or higher—mothers = 128 (64%) Education – college or higher—fathers = 292 (73.25%) SES < 5000 Yuan/month – mothers = (168) 84% SES < 5000 Yuan/month – mothers—fathers = 268 (67.25%) Ethnicity & parity = NR	*N* = 600, GA—mothers = mean 36.63 ± SD 3.34, GA – fathers = mean 37.09 ± SD 3.16 wks, BW—mothers = mean 2,926.70 ± SD 937.8 g BW- fathers = mean 3,051.90 ± SD 1028.9 g
Lotterman 2018^a^, [45] USA	Prospective cohort, 1 centre, NICU level III & IV, period & length of stay = NR	To investigate whether rates of psychopathology are elevated in mothers of moderate- to late preterm infants during/following infant hospitalization in the NICU, and associated protective and risk factors	Mothers of moderate- to late-preterm infants 32 to < 37 weeks	Mothers to babies born < 32 weeks or later than 36 weeks, or if they had been in the NICU > 6 months	*N* = 91 mothers at NICU admission, *N* = 76 at 6 months Age = mean 32.45 SD ± 6.78 yrs Ethnicity = Caucasian 37 (40.7%), African American 15 (17.4%) Asian 9 (10.5%), American Indian/Alaskan Native 2 (2.3%), 27 (29.1%) other Education—mean years = 14.29 ± SD 4.30 Parity, LWP & SES = NR	*N* = 91, GA = range 32–37 wks, GA = mean 33.53 ± SD, 1.33 wks, BW = NR
Misund 2013, 2014^a^, [36, 37] Norway	Prospective cohort, 1 centre at Oslo University Hospital, Norway, two periods: June 2005–January 2006 and October 2007–July 2008, NICU level & length of stay = NR	To explore the associations between maternal mental health problems & preterm birth & identify predictors of early mother–infant interaction	Mothers of preterm babies GA < 33 wks admitted to NICU	Mothers of severely ill babies that the medical staff estimated to have poor chance of survival, and non-Norwegian speakers were not included	*N* = 29 mothers at 2 wks post birth, *N* = 27 at 2 wks after NICU admission, *N* = 26 at 6 & 18 months post term, Age = mean age 33.7yrs ± SD 4.3 yrs Parity—nullips = 18 (62.1%) LWP = all SES – unemployed = 4 (13.8%) Education > 12 years = 26 (89.7%) Ethnicity = NR	*N* = 35, GA = median 29 range 24–32 wks mean 28.5 ± SD 2.6 wks, BW = range 623 to 2,030g, mean 1,222 ± SD 423 g
Moreyra 2021^a^, [47] USA	Cross-sectional, October 2017—July 2019, length of stay at least 14 days, number of centres, period & NICU level = NR	To describe the impact of depression, anxiety, and trauma screening protocol and the referral pf positively screened NICU parents	Parents of NICU babies admitted at least for 2 weeks	None excluded	*N* = 120 mothers and *N* = 30 fathers Age = 31.06 ± 6.26 yrs Parity – para = mean 1.95 ± SD 1.2 LWP = 91 (61%) Ethnicity = white 39 26%, Other = 111 (74%) Education & SES = NR	*N* = NR, GA = 32.3 ± 4.8 weeks, GA = 1935.2 ± 1052.1 g
Mulder 2014 [68], New Zeeland	Cohort, 1 centre, February 2001-January 2002, NICU level & length of stay = NR	To evaluate the psychological functioning in parents whose infants were admitted to a NICU over the first 2 years of the infant’s life	NICU admissions born to parents resident in a defined geographic area in a 12-month period were eligible for the study. Criteria for NICU admission were birth weight < 1800 g and < 34 weeks or any illness in the infant	No written informed consent	*N* = 242 mothers, *N* = 205 fathers Age—mothers = mean 30.1 ± SD 5.4 yrs Age- fathers = mean 33.1 ± SD 5.9 yrs LWP – mothers = 213 (88%) Education—professional qualification- mothers = 65 (52%) Education – professional—fathers = 50 (37%) SES—income < $70,000—mothers = 217 (89%) Parity & ethnicity = NR	*N* = 242, GA = range 23–42 wks, GA = mean 35.1 ± SD 3.8 wks, BW = mean 2477 ± SD 889.1 g
Okito 2022 [76], USA	Cohort, 1 centre, December 2017-October 2019, NICU level IV, length of stay = NR	To evaluate associations between parental resilience and psychological distress during NICU admission	Parents of infants ≤ 34 weeks GA and < 14 days of chronological age babies in level IV NICU	Not speaking English, not having custody, < 18 years	*N* = 45 mothers & fathers, mothers *N* = 39 (87%) Age—mothers & fathers = median 31, IQR 23–34 yrs LWP—mothers & fathers = 22 (49%) Education—Some college or more-mothers & fathers = 29 (64%) Ethnicity—mothers & fathers = Non-Hispanic White = 20 (44%) SES – employed—mothers & fathers = 25 (56%), Low concentrated disadvantage CDI < 0 22 (49%) Parity = NR	*N* = 45, GA = median 30, IQR 27.3–33 wks, BW = NR
Park 2022 [68], Korea	Cross-sectional, 1 centre, March 2018—August 2019, NICU level & length of stay = NR	To identify physical and emotional health status of preterm mothers, correlation between physical and emotional health, and differences according to characteristics of mothers and babies	Mothers ≥ 19 years who gave birth < 37 gestational weeks to infants admitted to the NICU, and who could understand and complete the study questionnaire	Congenital anomaly, required treatment (e.g. for continuous renal replacement therapy or extracorporeal membrane oxygenation)	*N* = 91 mothers Age = mean 35.3 ± SD 3.5 yrs Parity – nullips = 68 (74.7%) SES – employed = 54 (59.3%) Education ≥ university degree = 83 (91.2%) Ethnicity = South Korean, LWP = NR	*N* = 91, GA = range 23–36) wks, GA = mean 31.8 ± SD 2.8 wks, BW = mean 1,489.5 ± SD 543.3 g
Pisoni 2020^a^, [49] Italy	Prospective cohort, 1 centre, August 2013- April 2014, length of stay = mean 29, range 13–138 days, NICU level = NR	To examine the relationships between maternal psychological distress, parental protective factors, perinatal risk factors and neurodevelopmental outcomes	Preterm infants gestational age < 34 weeks and their mothers aged > 18 years old with an adequate grasp of Italian and ability to understand purpose of study	Congenital anomalies and infections, maternal psychiatric illness and/or drug abuse	*N* = 29 mothers Age = mean 32.8 ± SD 6.7 yrs Parity – nullips = 19 (65.5%) SES—employed = 25 (86.2%) Education = mean 14.3 ± SD 2.8 yrs Ethnicity & LWP = NR	*N* = 29, GA = mean 30.2 ± SD 3.2 wks, range 23–33 wks, BW = mean 1528.9 ± SD 541.2, range 574–2327 g
Rogers 2013 [69], USA	Prospective cohort, 1 centre, 3 year-period, level III NICU, length of stay = mean 90.5 ± SD 28.6 days	To identify factors for mothers at-risk for postpartum depression or anxiety at the time of NICU discharge among Caucasian and African-Americans	Mothers to preterm infants born < 30 weeks	Mothers to babies with congenital anomaly or being moribund with severe sepsis or respiratory failure in the first days of life	*N* = 73 mothers Age = mean 27.2 ± SD 7.4 yrs Parity—nullips = 25 (34.3%) LWP = 31 (42.5%) SES—public insurance = 50 (69.0%) Education—college or higher = 39 (53.5%) Ethnicity = Caucasian 36 (49%), African 37 (51%)	*N* = 73, GA = mean 25.5 ± SD 1.8 wks, BW = NR
Serge 2014 [70], USA	Cross-sectional, 1 centre, December 2010-May 2012, NICU level IV, length of stay = NR	To identify risk factors of aversive emotional states in NICU mothers and the significant risk factors based on conceptual model	Mothers of NICU infants, ≥ 18 years of age, English speaking	Cases with missing data excluded from analysis	*N* = 200 mothers Age = mean 28 yrs, range 18—45 LWP = 123 (61.8%) SES—employed = 132 (66.3%) Education = 14.6 (2.5) yrs Ethnicity = White 178 (90%) Parity = NR	*N* = 199, GA, = 23—41 wks, BW = range 397–4,706 g
Shivhare 2022 [77], India	Case–control, 1 centre, January-December 2018, length of stay = 7 to > 30 days, NICU level = NR	To compare psychological outcomes of mothers to term and preterm babies admitted to NICU	Mothers of term and preterm babies admitted to NICU	Mothers with serious physical conditions that might have an impact on psychological well-being	*N* = 100 mothers Age = mean 26.14 ± SD 4.49 yrs LWP = NR SES—rural = 50 (50%) semi urban = 34 (34%) urban 16 (16%) Education—college = 18 (18%), parity = NR	*N* = 100, GA = 76% < 37 wks, 24% ≥ 37 GA = mean 35.39 ± SD 1.53 wks BW = 14% 2.5 kg, 69% > 1.5–2.4kg, 17% < 1.5 kg
Treyvaud 2016 [71], Australia	Prospective cohort, 1 centre, 2001 and 2003, NICU level & length of stay = NR	To assess multiple births and bereavement impact on mental health, parenting stress and family functioning	Families from the Victorian Infant Brain Studies (VIBeS) cohort to infants born at < 30 wks' gestation or with a birthweight < 1250 g	Not completing the questionnaire fully	*N* = 162 mothers (singleton 129 and multiples 33) Age—singleton and multiples = mean 30 ± SD 6 yrs Parity—singleton nullips—= 85 (66%) Parity—multiples nullips = 24 (75%) LWP—singleton = 110 (85%) LWP—multiples = 32 (97%) SES—professional jobs singleton = 44 (34%) SES – professional jobs multiples = 4 (13%) Education—tertiary singleton = 18 (14%) Education – tertiary multiples = 1 (1%) Ethnicity = NR	*N* = 194 (129 singletons, 65 multiples), GA < 30 wks, BW < 1250 g
Vizcarrondo-Oppenheimer 2021 [78], USA	Cross-sectional, 1 centre, 2015–2018, NICU level & length of stay = NR	To examine the link between multiple risk factors of anxiety and depression in mothers of NICU babies	Mothers to infants admitted to NICU since birth	NR	*N* = 92 mothers Age = mean 27.64 ± SD 7.11 yrs SES—major professional = 8 (8.7%), minor professional 16 (17.4%), skilled worker 22 (23.9%), semiskilled worker 24 (26.1%), unskilled worker = 20 (21.7%) Parity, LWP, education & ethnicity = NR	*N* = NR, GA = mean 30.75 ± SD 5.16 wks, BW = mean 1,540.3 ± SD 864.92 g
Zanardo 1998 [72], Italy	Case–control, 1 centre, NICU level III, length of stay = mean 40 ± SD 32 days, period = NR	To identify predictors of pre-discharge parental anxiety in parents of preterm twins	Parents of high-risk premature twins (mean birth weight 1.493 ± 227 kg; mean gestational age 33 ± 3.5 weeks), admitted to level III NICU	NR	*N* = 55 parents, *N* = 30 mothers, *N* = 25 fathers Age -twins mothers = mean 33 ± SD 5.8 yrs Age—twins fathers = mean 33 ± SD 4.2 yrs Age—singleton mothers = mean 32 ± SD 4.9 Age—singleton fathers = mean 36 ± SD 5.6 years Education—twins = doctorate = none Education—twins = doctorate fathers = 1 Education—singleton doctorate mothers = 1 Education—singleton doctorate fathers = 2 Parity, LWP, SES, ethnicity = NR	*N* = 45 (15 twins, 15 singleton), GA twins = mean 33 ± SD 3.5 wks GA singletons = mean 33 ± SD 3.5 wks BW twins = mean 1.493 ± SD 227g BW singleton = 1.878 ± 1.151 g

*Abbreviations*: *BW* birth weight, *CCA* Chronological Corrected Age, *CDI* Concentrated Disadvantage Index, *GA* Gestational age, *LWP* Living with partner, *N* number of participants, *Nullips* Nulliparous, *NR* Not reported, *PRI* Perinatal Risk Inventory, *SES* Socioeconomic status, *Tertiary* secondary school, < secondary school), *Wks* weeks, *yrs* years

^a^Studies included in both anxiety and post-traumatic stress (PTS): Garfield 2015 [27], Greene 2015 & 2019 [28, 29], Holditch-Davis 2009 [31], Lotterman 2018 [45], Misund 2013 & 2014 [36, 37], Moreyra 2021 [47], Pisoni 2022

Twelve studies, published in 13 records, came from USA [27–29, 31, 45, 47, 57, 64, 67, 69, 70, 76, 78]. Six studies, published in seven records, were from Europe [36, 37, 49, 56, 58, 59, 72]. Two studies were from Brazil [62, 66] and two from Australia [71, 75], and one was from each of the following countries: New Zealand [68], Canada [65], China [74], Korea [79], Iran [63], Turkey [60], Tunisia [73] and India [77].

Eight studies involved both parents [47, 58, 65, 68, 72, 74, 75], one either parents [76],one [59] included only fathers, and the remaining 21 studies included only mothers. One study only included mothers of babies with congenital anomalies [66], two studies [71, 72] compared multiples to singletons and one study [71] compared bereaved to non-bereaved parents.

GA of the infant was an inclusion criterion in 17 studies, published in 18 records [27, 36, 37, 45, 49, 56, 58, 61–63, 65, 67–69, 71, 75, 76, 79], and BW was a criterion in eight studies, published in nine records [27–29, 31, 61, 65, 67, 68, 71]. One study [72] used both GA and BW to define preterm infants. The studies used various measures of general anxiety symptoms.

### Risk of bias assessment

**Fig. 3:**
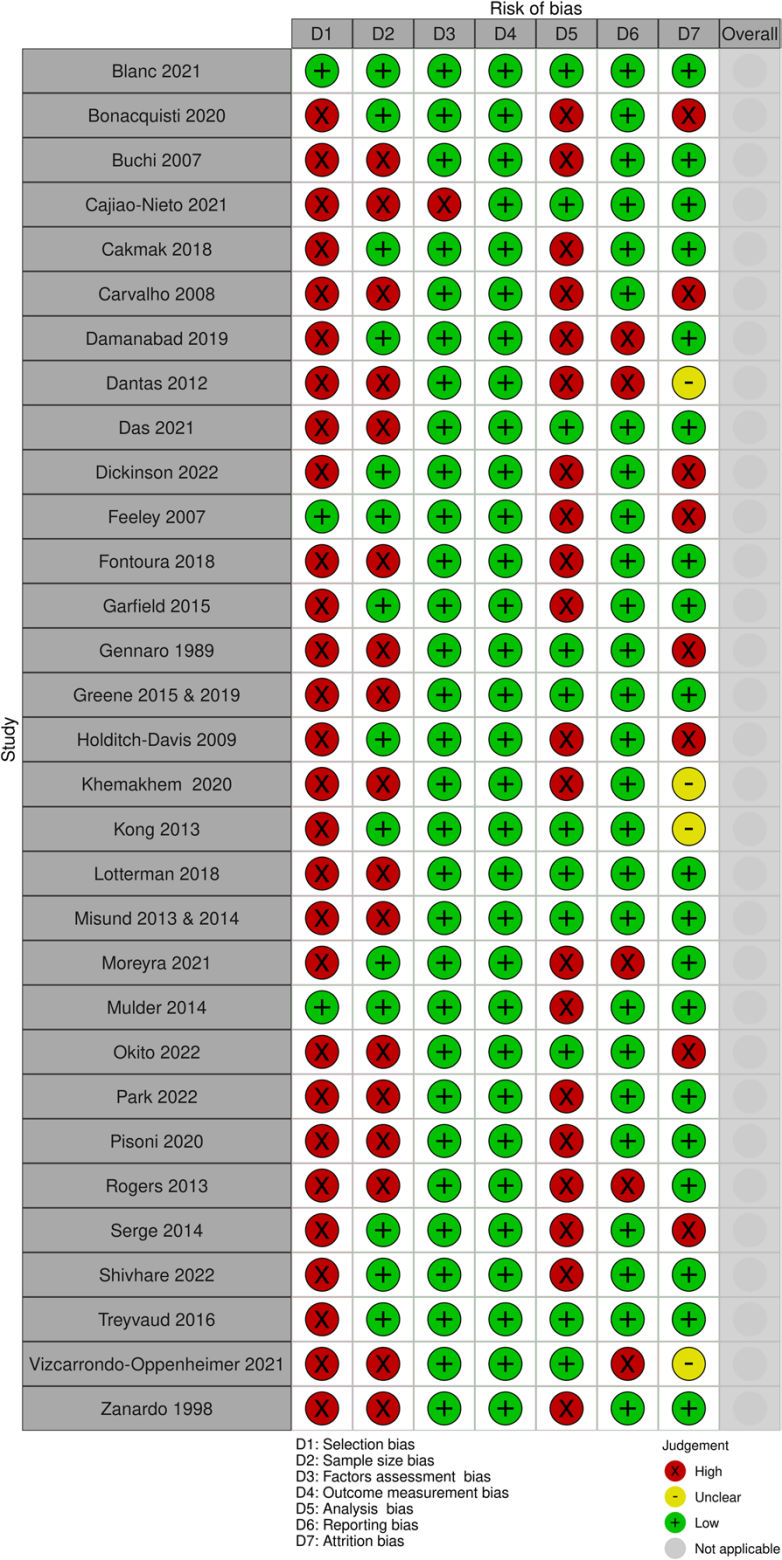
Risk of bias summary of anxiety included studies

One study [56] was rated at low risk of bias across all domains (See Fig. 3-A summary of risk of bias of anxiety studies and Appendix 3). In the remaining studies, sample selection bias was low in two studies only [65, 68]. Bias due to sample size was low in 14 studies [27, 31, 47, 56, 57, 60, 63, 65, 68, 70, 71, 74, 75, 77]. All except one study [59] used valid measures to assess the factors. Anxiety was assessed via standardised measures in all studies. The bias in the analysis domain was low in eleven studies, published in thirteen records [28, 29, 36, 37, 45, 56, 59, 64, 67, 71, 74, 76, 78]. Reporting bias was low in all except five studies [47, 62, 63, 69, 78] and attrition bias was low in all except in eight studies where it was high [31, 57, 61, 65, 67, 70, 75, 76] and unclear in four [62, 73, 74, 78].

### Factors associated with anxiety

Overall, 5,941 parents were involved across the 31 included studies with sample sizes ranging from 29 to 2270 participants. A total of 73 potential risk factors were identified. The risk factors are detailed in Table 5, mapped in Table 6, and summarised here using the same eight categories as for PTS.

- 1) Parent demographic factors (11 factors: age, education, sex, couple’s relationship, family income, employment, ethnicity, residential area, medical insurance, smoking, cumulative psychosocial risk factors).

**Table 5:** Summary of factors reported in anxiety included studies

**Study ID**	**Study population**	**Time of Anxiety assessment**	**Anxiety measuring tool and cut-off points**	**Parents with anxiety, N, n (%)**	**Statistical analysis**	**Factors data** (Assessment time)					
**Blanc 2021 **[56]	Mothers to infants born 22–34 wks	At neonatal discharge	STAI > 45	Mothers *N* = 2270 state *n* = 1856 (83.0%), *n* = trait 1393 (63.4%)	Multivariate Analysis	**Factors** (neonatal discharge)		**aRR (95% CI) state**			**aRR (95% CI) trait**
						Age, years		*			0.99 (0.98, 1.00)
						Married/living with partner		*			0.87 (0.74, 1.01)
						Unemployment vs employment (1)		0.96 (0.90, 1.03)			1.01 (0.90, 1.13)
						**History of psychiatric** **Disorders yes vs no**		**1.11 (1.01, 1.21)**			1.19 (1.00, 1.42)
						Nulliparity		1.04 (0.98, 1.10)			*
						Type of pregnancy multiple vs singleton (1)		1.02 (0.97, 1.08)			0.94 (0.83, 1.07)
						**Vaginal delivery ≥ ** **26 weeks** **vs CS ≤ 26 weeks (1)**		**1.12 (1.04, 1.20)**			1.00 (0.90, 1.10)
						Vaginal delivery < 26 weeks vs CS ≥ 26 weeks (1)		1.12 (0.97, 1.30)			1.13 (0.92, 1.39)
						CS < 26 weeks vs CS ≥ 26 weeks (1)		0.95 (0.77, 1.17)			1.13 (0.88, 1.44)
						Premature delivery induced vs spontaneous delivery (1)		1.05 (0.98, 1.13)			*
						Preeclampsia Complications yes vs no		1.04 (0.88, 1.23)			*
						Severe maternal morbidity^b^ yes vs no		0.97 (0.88, 1.06)			*
						Meeting newborn later after birth vs right after birth (1)		1.01 (0.96, 1.06)			*
						Skin to skin after birth yes vs no		1.09 (0.98, 1.21)			1.10 (0.95, 1.28)
						BW		*			1.00 (0.99, 1.00)
						Type of neonatal room single room vs room with baby		*			0.89 (0.75, 1.05)
						Multiple room vs room with baby		*			0.89 (0.77, 1.04)
						Severe neonatal Morbidity^c^		1.01 (0.94,1.10)			0.92 (0.77, 1.10)
						Hospital transfer or social nursery or other vs home discharge (1)		0.97 (0.91, 1.03)			*
						Adjusted for birthweight and severe neonatal morbidity, among other factors, * Factors in univariate analysis not significant including breastfeeding, maternal admission to ICUF					
**Bonacquisti 2020 **[57]	Mothers to NICU infants	During NICU and 2–4 months later	DAS > 21	Mothers *N* = 127, *n* = 23 (17.8%)	Correlation	**Factors** (during NICU)		***r***			***P***
						**Maternal-infant attachment**		**-0.20**			**0.03**
**Buchi 2007 **[58]	Parents to deceased infants born at 24–26 wks	2–6 years since death	HADS > 7	Parents *N* = 54, *n* = 15 (28%)	Correlation	**Factors** (2–6 years)		**Parents** **r, P**		**Mothers** **r, P**	**Fathers** **r, P**
						**Bereavement- MTS-total**		**0.33, < 0.01**		**0.51, < 0.01**	0.05, NS
						Grief- MTS-sad		0.14, NS		0.31, NS	-0.12, NS
						PTGI		-0.08, NS		-0.25, NS	-0.02, NS
						Suffering-PRISM-SBS		-0.13, NS		-0.31, NS	0.12, NS
						Parent -sex fathers vs mothers		*P* = NS			
**Cajiao-Nieto 2021 **[59]	Fathers to NICU infants	Day 3 after birth (T1), 15–20 days after birth (T2)	STAI > 28 (from author)	Fathers *N* = 51, T1 *n* = 17 (33%), T2 *n* = 5 (9.8%)	Bivariate analysis using Student’s T-test	**Factors** (15–20 days after birth)		**STAI-state at T1** **Mean scores, P**			**STAI-state at T2** **Mean scores, P**
						Personal mental health history yes vs no		25.0 vs 24.12, NS			20.5 vs 18.42, NS
						History of mental health problem of any member yes vs no		29.13 vs 23.45, NS			24.9 vs 17.6, NS
						**Couple relationship good vs bad**		23.85 vs 30.67, NS			**18.17 vs 28.0, < 0.01**
						GA ≤ 28 week vs > 28		34.0 vs 22.96, NS			22.33 vs 18.27, NS
						BW ≤ 1500g vs > 1500g		23.92 vs 24.58, NS			19.24 vs 18.27, NS
						Singleton yes vs no		22.77 vs 25.38, NS			17.64 vs 19.59, NS
						ART yes vs no		20.0 vs 26.78, NS			16.26 vs 20.22, NS
						CS vs vaginal		21.14 vs 28.36, NS			17.72 vs 20.09, NS
						**Perceived social support yes vs no**		**24.37 vs 23.20, < 0.05**			18.8 vs 18.2, NS
						**First time fathers vs not**		**22.42 vs 29.62, < 0.01**			18.05 vs 20.77, NS
						**Health risk for the infant detected prenatally yes vs no**		24.13 vs 24.62, NS			**18.39 vs 19.77, < 0.05**
						Duration of infant hospitalisation: < 15 days vs 15 days -1 month vs > 1month		22.91, 24.00, 25.04, NS			19.45, 16.88, 19.67, NS
						Duration of mother hospitalization < 48 h vs 3–5 days 6–10 days vs > 10 days		20.13, 22.08, 23.31, 28.59, NS			14.63, 22.15, 17.08, 19.35, NS
						**Unemployed,** **Father income,** **Mother income,** **Both incomes**		**6.00, 22.09, 32.50, 25.47, P < 0.05**			11.00, 18.36, 18.50, 19.31, NS
						**PSS**		**STAI-S at T1** **r, P**			**STAI-S at T2** **r, P**
						**Stress: sights and sounds**		**Metric 1: 0.27, < 0.05** Metric 2: 0.25, NS			Metric 1: 0.37, NS Metric 2: 0.33, NS
						**Stress: infant behaviour appearance**		**Metric 1: 0.36,** ** < 0.01** **Metric 2: 0.04,** ** < 0.01**			**Metric 1: 0.45, < 0.01** **Metric 2: 0.47,** ** < 0.01**
						**Stress: parental role alterations**		**Metric 1: 0.36, < 0.01** **Metric 2: 0.36, < 0.01**			**Metric 1: 0.29, < 0.05** **Metric 2: 0.33,** ** < 0.05**
						Stress: staff behaviour and communication		Metric 1: 0.13, NS Metric 2: 0.17, NS			Metric 1: 0.15, NS Metric 2: 0.21, NS
						**Stress: overall score**		**0.45, < 0.01**			0.19, NS
**Cakmak 2018 **[60]	Mothers to NICU infants	During NICU admission	STAI ≥ 37	Mothers *N* = 340, *n* = NR	Correlation	**Factors** (During NICU)		**STAI State** **r, P**			**STAI Trait** **r, p**
						**STAI Trait**		**0.33, < 0.01**			**NA**
						**Problem-solving process subscale**		**-0.58, < 0.01**			**-0.36, < 0.01**
						**Baby care skills-subscale**		**-0.35, < 0.01**			**-0.27, < 0.01**
						**Participation in caregiving observation**		**-0.48, < 0.01**			**-0.12, < 0.05**
**Carvalho 2008 **[61]	Mothers of infants < 37 wks and BW ≤ 1500 g	During NICU, post discharge, at 12 months corrected age	STAI, ≥ 75th percentile	Mothers *N* = 36, State anxiety *n* = 12 (86%), state *n* = 8 (57%)	Correlation	**Factors** **(**Time not specified)		**STAI State,** **r, P**			**STAI Trait** **r, P**
						**Academic level (grade)**		**-0.33, 0.05**			**-0.44, 0.01**
						**Parity—Greater number of children**		**0.35, 0.03**			NR
						**BW**		**-0.53, 0.001**			NR
						**Duration of stay in NICU**		**0.46, 0.005**			NR
						**Total duration of hospitalization**		**0.46, 0.004**			**0.37, 0.03**
						**CRIB—babies**		**0.37, 0.03**			NR
**Dantas 2012 **[62]	Mothers to preterm infants	During NICU stay	STAI > 40	Mothers *N* = 70, *n* = (81.7%) had intense symptoms of anxiety state, 70% of anxiety trait,	Correlation	**Factors** (During NICU)		**STAI State** **r, P**			**STAI Trait** **r, P**
						**Postnatal depression**		**0,59, 0.001**			**0.54, 0.001**
						**Pregnancy complications**		**NR, 0.02,**			**NR, 0.04**
						Maternal age, education, number of children, number of antenatal consultations**,** marital status, income, mode of delivery		Not significant, data NR			
						BW, GA, length of stay, Apgar		Not significant, data NR			
**Damanabad 2019 **[63]	Mothers to preterm babies 30–36 weeks	One day post delivery	STAI, mild 20–40 moderate 41–60, severe 61–80	Mothers *N* = 100, state mean ± SD = 48.62 ± 6 trait state mean ± SD = 32.45 ± 3.63	Analysis of variance (ANCOVA)	**Factors** (One day after birth)		**STAI State** **Mean ± SD, P value**			**STAI Trait** **Mean ± SD, P value**
						**Greater number of children**		**48.66 (2.56), 0.01**			30.49 (1.61), 0.690
						**Education level**		**48.20 (1.51), 0.045**			31.54 (1.17), 0.246
						**GA**		**49.26 (1.91), 0.027**			32.10 (1.50), 0.264
						**Child order**		**49.22 (2.53), 0.002**			**33.35(1.58), 0.477**
						Mothers’ ages, delivery method, employment, income, infant gender, BW		NR < *P* > 0.05			NR
						**Factors STAI state**		**Mean ± SDs**		**95%CI**	***P*** ** value**
						**Multips ≥ 3 delivery vs prims**		**55.94 ± 2.37 vs NR**		**51.22 to 60.66**	**0.00**
						**University** **Degree vs diploma vs < diploma**		**50.47 ± 1.76 vs 46.93 ± 1.33 vs 51.68 ± 1.80**		**46.96 to 53.99**	**0.04**
						**GA ≤ 33 wks vs > 33 weeks**		**55.20 ± 2.26 vs NR**		**50.7 to 59.7**	**0.003**
						**Three and over child order vs < 3 children**		**41.28 ± 2.41 vs NR**		**36.47 to 46.10**	**0.00**
**Das 2021 **[64]	Mothers of NICU infants, low income area	During NICU	DASS-21, > 21	Mothers *N* = 96, 46 (48%)	T-tests or Mann–Whitney parametron and non-parametric test	**Factors** (During NICU)				***P*** ** value**	
						**Previous history of depression**				** < 0.02**	
						Adjusting for maternal drug abuse, birth weight and maternal gravidity					
**Dickinson 2022 **[77]	Mothers and fathers to babies ≤ 37 wks GA	< 2 wk post birth and at 3 months post birth	DASS > 21	At 2 weeks: Mothers *N* = 69, *n* = 18 (26.8%) & fathers *N* = 45, *n* = 5 (11.1%) At 3 months: Mothers *N* = 48, *n* = 5 (10.6%) & fathers *N* = 31, *n* = 3 (9.7%)	Difference between mothers and fathers	**Factors** (< 2 wks and 3 months after birth)		**OR (95%C)**			***P***
						Parents gender (Mothers vs fathers) < 2weeks		*2.82 (0.96, 8.26)*			0.06
						Parents gender (Mothers vs fathers) at 3 months		*1.09 (0.24, 4.90*]			0.9
**Feeley 2007 **[65]	Mothers and fathers of very low BW infants < 1500 g	3 and 9 months of corrected age	STAI, 20 items scored on a 4-point scale	Couples *N* = 61, at 3 and 9 months mothers (46.2 & 46.7), fathers (46.8 and 45.9)	Repeated-measures ANOVA	**Factors** ( 3, 9 months)	**3 months**	**9 months**		**Effect**	**F**
						Mothers	46.25	46.82		parent	F(1, 60) = 0.01
						Fathers	46.74	45.92		time	F(1, 60) = 0.48
										interaction	F(1, 60) = 0.69
**Fontoura 2018 **[66]	Mothers to infants with congenital anomalies	Prenatal vs postnatal	STAI, low (percentile < 25), moderate (25–75) and high (> 75)	Mothers *N* = 115, trait low *n* = 31 (27%), moderate *n* = 62 (53.9%), high *n* = 22 (19.1%) State: low *n* = 33 (28.7%), moderate *n* = 54 (47.0%), high *n* = 28 (24.3%)	Kolmogorov–Smirnov test	**Factors** (Prenatal vs postnatal)	**STAI ± State** **Mean ± SD (trait), P**				**STAI Trait** **Mean ± SD, P**
						**Prenatal anomaly diagnosis vs postnatal anomaly diagnosis**	**47.76, 8.298 vs 45.49, 9.310**	**0, 172**		**37.4,** **7.034 vs 41,27, 11,135**	**0.026**
**Garfield 2015**^**a**^[27]	Low income mothers to very Low BW < 1500 g infants	First 3 months after birth	STAI, 20 items rated on a 4-point Likert scale (1 = not at all and 4 = very much so)	Mothers *N* = 113, mean ± SD 39.1 12.6	Correlation	**Factors** (3 months after birth)		**STAI State** **r**			***P***
						Postpartum depressive symptoms		**0.66**			** < 0.01**
						Postpartum PTSS		**0.51**			** < 0.01**
						Maternal age > 35		0.05			NS
						Infant illness—Neurobiologic Risk Score (NBRS)		**0.31**			** < 0.01**
						Education		-0.11			NS
						Parental stress		**0.43**			** < 0.01**
**Gennaro 1988** [67]	Mothers of preterms	Wk 1, 2, 3,4,5,6,7 after birth	STAI, NR	Mothers *N* = 41, mean ± SD 37.6 ± (7.1)	Multivariable analysis	**Factors** (Across all time points)		**F**		**df**	***P***
						Child’s health		0.81		2.38	0.45
**Greene 2015**^**a**^[28]	Mothers to very low BW infants < 1500g	T1 (mean 28.1 days after birth) and T2 (mean 14.8 days prior to NICU discharge)	STAI > 40	Mothers *N* = 69, *n* = 38 (55%) at T1 *N* = 23 (36%) at T2	Multivariable logistic regression	**Factors (**at T2 prior NICU discharge)		**OR**		**95%CI**	***P***
						**Postnatal depression**		**1.21**		**1.09–1.30**	** < 0.001**
						**Primipara**		**7.21**		**1.54, 33.79**	** < 0.05**
**Greene 2019**^**a**^[29]	Mothers to very low BW < 1500g	T1 (mean 28.1 days after infants’ birth),T2 (mean 14.8 days prior to infants’ NICU discharge); T3 (infants’ 4 month CA follow-up visit); T4 (infants’ 8 month CA follow-up visit)	STAI > 40	Mothers *N* = 69, mean 41.86	Multilevel linear growth modelling	**Factors** (T1 after birth)		**Mean**		**95%CI**	***P***
						**Increase BW by 100 g**		** − 0.02**		** − 0.03 to − 0.00**	**0.02**
						**Lower Income status yes vs no**		** − 11.2**		** − 21.20, − .84**	**0.03**
						Maternal Education (per year)		− 2.14		− 4.51, 0.23	0.07
**Holditch-Davis 2009**^**a**^[31]	African-American Mothers to < 1500 g infants	During NCU admission 2, 6, 12, 18, and 24 months after term	STAI > 47	Mothers *N* = 117, state anxiety mean ± SD = 39.8 ± 13.6	Correlation	**Factors** (During NICU)		**STAI State ** ***r***			***P***
						**Infant appearance stress**		**0.39**			** < 0.001**
						**Parental role alteration stress**		**0.45**			** < 0.001**
						**Depressive symptoms at enrolment**		**0.73**			** < 0.001**
**Khemakhem 2020 **[73]	Mothers to NICU infants	During NICU	HADS > 11	Mothers *N* = 10, *n* = 2 (20%)	Correlation	**Factors** (During NICU)		***r***			***P***
						BW		-0.245			NS
						Apgar at 1 min		-0,338			NS
						**Postnatal depression**		**0.759**			** < 0.00**
**Kong 2013 **[74]	Mothers and fathers to NICU infants	During first wk after birth	Zung Self-Rating Anxiety Scale (SAS) > 50	Mothers *N* = 280, *n* = 48 (24%), fathers *n* = 80 (20%)	Univariate analysis	**Factors** (During first wk after birth)		**Mean ± SD**	**t/F**		***P***
						Anxiety fathers vs mothers		40.42 ± 10.76 vs 41.35 ± 10.26	-0.295		0.768
						Age < 20		42.8 ± 8.93	*F* = 0.953		0.414
						Age 20–29		40.95 ± 10.37			
						Age 30–39		39.56 ± 10.74			
						Age ≥ 40		41.81 ± 12.47			
						Primary school		46.60 ± 10.43	**10.31**		**0.000**
						Senior High School		45.08 ± 10.49			
						Junior High School		43.35 ± 10.84			
						College or higher		39.01 ± 10.13			
						**Medical insurance**		**39.38 ± 9.92**	**3.531**		**0.000**
						**No medical insurance**		**42.53 ± 11.35**			
						**Area of residence urban**		**30.99 ± 10.48**	**-3.581**		**0.000**
						**Rural resident**		**43.59 ± 10.53**			
						Household income (Yuan/month)					
						< 5000		40.93 ± 10.43	1.531		0.217
						5000–10,000		39.68 ± 10.40			
						> 10.000		38.52 ± 11.85			
**Lotterman 2018**^**a**^[45]	Mothers of moderate- to late-preterm infants	During NICU stay	Generalized Anxiety Disorder-7 Item (GAD-7) ≥ 10	Mothers *N* = 91, *n* = 23 (24.7%)	Multivariable linear regression	**Factors** (During NICU)		**B**	**SE**		***P***
						Previous mental illness (MH)^d^		52.19	27.36		NS
						Mother infant contact (MI)		-0.55	0.49		NS
						MH X MI		-4.92	2.91		NS
						**Infant Health Problems (HP)**		**15.80**	**5.62**		** < 0.01**
						Mother-Infant Contact (MI)		0.09	0.64		NS
						**HP X MI**		**-1.52**	**.58**		** < 0.01**
						**Forward-Focused (FF) Coping**		**-0.69**	**0.25**		** < 0.01**
						**Mother-Infant Contact (MI)**		**-4.91**	**1.92**		** < 0.01**
						**FF x MI ( physical/verbal)**		**0.06**	**.03**		** ≤ 0.05**
		6 months after the first assessment		Mothers *N* = 76, *n* = 18 (27.6%)	Multivariable linear regression	**Factors** (6 months after first assessment)		**B**	**SE**		***P***
						Baseline anxiety		-0.01	0.18		NS
						Mother visits per week		-.074	0.74		NS
						Positive mother-nurse interaction		-1.66	1.92		NS
						**Mother-understands explanations**		**-3.77**	**1.61**		** ≤ 0.05**
						Mother-technical questions		-0.25	1.15		NS
						Mother-asks how to baby care		-2.56	1.83		NS
						**Step 1**					
						Anxiety (Baseline)		0.12	0.09		NS
						Previous Mental Illness		3.28	1.72		NS
						Optimism		-0.18	0.11		NS
						Length of stay (days)		0.02	0.02		NS
						Infant health problems		0.74	0.50		NS
						**Step 2**					
						Anxiety (Baseline)		0.13	0.09		NS
						Previous Mental Illness		4.00	6.32		NS
						Optimism		-0.21	0.21		NS
						Length of Stay (days) (LOS)		0.1	0.05		NS
						Infant Health Problems (IHP)		1.35	2.19		NS
						Previous MI x Optimism		-0.08	0.35		NS
						LOS x Optimism		0.0	0.0		NS
						IHP x Optimism		0.10	0.10		NS
**Misund 2013 & 2014**^**a**^[36, 37]	Mothers to infants < 33 weeks	within 2 wks postpartum, 2 wks after hospitalization, 6 months post-term, 18 months post-term	STAI ≥ 40	Mothers *N* = 29, *n* = 5 (17%)	Multivariable linear regression	**Factors** (6 and 18 months post-term ages)		**STAI State B (unstandardized)**	**95%CI**		***P*** ** value**
						**Maternal trait anxiety** (Misund 2013)		**0.23**	**0.10, 0.36**		**0.001**
						Parity (Misund 2014)		**0.42**	**0.63, 2.79**		**0.003**
						**GA** (Misund 2014)		**0.57**	**0.28, 0.86**		**0.000**
**Moreyra 2021**^**a**^[47]	Mothers and fathers to NICU infants	14 days post NICU admission	Neuro-Qol Anxiety Short Form = 60	Mothers *N* = 120 and fathers *N* = 30 fathers, mean ± SD 54.2 ± 35.7	Difference between the groups using t-test and correlation analysis						
						**Factors** (During NICU stay at 14 days post admission)		**T test**			***P***
						**Parents sex mothers vs fathers**		**4.13**			** < 0.0001**
						Ethnicity		NR			NS
						Correlation		R			P
						**Post-traumatic stress symptoms**		**0.79**			** < 0 .001**
						**Depression**		**0.81**			** < 0 .001**
**Mulder 2014 **[68]	Mothers and fathers to NICU infants	During NICU, 9 months	HADS > 11	Parents *N* = 447, mothers = 242, *n* = 35 (18%), fathers 205, *n* = 20 (11%)	Mean difference	**Factors** (During NICU and at 9 months)		**Mean difference**	**95%CI**		***P***
						**Parents sex fathers vs mothers during NICU**		***-0.90***	***-0.95, -0.85***		** < *0.00***
						**Parents sex fathers vs mothers at 9 months**		***-0.70***	***-0.74, -0.66***		** < *0.00***
**Okito 2022 **[75]	Parents to infants born < 34 wks admitted to level IV NICU	During NICU, assessment 1 at 2 wks post birth, assessment 2 at 6 wks post birth	STAI ≥ 40	Mothers or fathers *N* = 45, *n* = 14 (31%) at 2 weeks post birth, *n* = 5 (24%) at 6 wks post birth	Multiple regression	**Factors** (2 wks post birth)		**aOR**	**95%CI**		***P***
						Resilience-CD-RISC score		0.95	0.89, 1.02		0.14
						Perceived social support (family, friends, others)-MSPSS score		0.96	0.90, 1.02		0.26
						Infant clinical severity (number of active diagnoses and medications, level of respiratory support, recent surgery, and need for life-sustaining medications)		1.07	0.89, 1.27		0.48
						**Low disadvantage (CDI < 0) (greater economic advantage)**		**6.50**	**1.26, 33.34**		** < 0.05**
						Adjusted for resilience, social support, Infant Clinical Severity Scores and parental education					
**Park 2022, **[78]** Korea**	Mothers to infants GA < 37 wks	2 to 3 wks after birth	STAI, cut off NR	Mothers *N* = 91, state anxiety mean ± SD = 48.04 ± SD 13.24	Correlation and t-tests	**Factors** (2 to 3 wks after birth)		**STAI state ** ***r***	***P***		
						**Postpartum depression**		**0.84**	**0.001**		
						**Maternal physical health problems**		**0.34**	**0.001**		
						**Guilt feelings scores**		**0.72**	**0.001**		
						**Mothers & infants characteristics**		**STAI mean ± SD**	**T**		***P***
						Mothers age ≤ 35 years		49.92 ± 13.32	1.43		0.16
						Mothers age > 35 years		45.95 ± 12.97			
						Education ≥ University		48.34 ± 13.38	0.68		0.50
						Education high school		45.00 ± 12.03			
						Employed		48.06 ± 11.47	0.01		0.99
						Unemployed		48.03 ± 15.63			
						**Primipara**		**49.85 ± 12.72**	**2.29**		**0.02**
						Multipara		42.70 ± 13.57			
						Multiple births		49.85 ± 13.11	1.51		0.14
						Singleton birth		45.64 ± 13.19			
						Education on postnatal care yes		43.61 ± 15.25	1.60		0.11
						No education on postnatal care		49.14 ± 12.57			
						Natural pregnancy		48.58 ± 13.04	0.34		0.74
						Artificial pregnancy		47.63 ± 13.50			
						Male infants		48.24 ± 13.76	0.14		0.89
						Female infants		47.85 ± 12.86			
						GA					
						**Gestational age < 34 weeks**		**50.45 ± 12.76**	**2.48**		**0.02**
						Gestational age ≥ 34 weeks		43.39 ± 13.10			
						**Ventilator care**		**54.50 ± 11.77**	**3.45**		**0.001**
						No Ventilator care		44.87 ± 12.84			
						**Oxygen treatment**		**50.14 ± 12.98**	**2.32**		**0.02**
						No oxygen treatment		43.32 ± 12.79			
						**Antibiotic treatment**		**50.93 ± 14.01**	**2.72**		**0.008**
						No antibiotic treatment		43.43 ± 10.52			
						**Still in hospital** at the time of assessment** (length of stay)**		**50.33 ± 13.05**	**2.27**		**0.007**
						Discharge at the time of assessment		42.00 ± 11.98			
**Pisoni 2020**^**a**^[49]	Mothers to infants born < 34 wks	During NICU stay & 12 months infant corrected age	STAI, cut off NR	Mothers *N* = 29, during NICU state *n* = 16 (55%), trait *n* = 13 (45%) at 12 months state *n* = 8 (28%), trait *n* = 9 (31%)	Correlation	**Factors** (During NICU**)**		**STAI State ** ***r*** **, ** ***P***	**STAI Trait ** ***r*** **, ** ***P***		
						**Perinatal risk inventory (PERI)**		**0.47**, < 0.05	0.27, NS		
						**Generalised Developmental Quotient (GQ)**		-0.40, NS	-0.24, NS		
						**Dyadic Synchrony Care Index**		-0.03, NS	0.157, NS		
						**Factors** (at 12 months**)**					
						**Perinatal risk inventory (PERI)**		-0.07, NS			0.025, NS
						**Generalised Developmental Quotient (GQ)**		-0.15, NS			-0.172, NS
						**Dyadic Synchrony Care Index**		0.191, NS			0.128, NS
**Rogers 2013 **[69]	Mothers of infants born < 3 0 wks	NICU discharge	STAI > 40	Mothers *N* = 73, *n* = 31 (43%)	Correlation	**Factors** (NICU discharge)		**STAI State ** ***r***			***P***
						Maternal age		NR			NS
						Race		NR			NS
						Education		NR			NS
						Marital status		NR			NS
						Insurance status		NR			NS
						Prior history of depression or anxiety		NR			NS
						Smoking		NR			NS
						Stressful life events		NR			NS
						Stress related parental role alteration		NR			NS
						Social support satisfaction		NR			NS
						Number of days of ventilation		NR			NS
						Length of stay		NR			NS
						Severity of brain injury		NR			NS
						Number of children		t 0.10			0.92
						**STAI trait scores**		**0.4**			**0.001**
**Serge 2014 **[70]	Mothers to NICU infants majority white	During first week of NICU stay	Beck Anxiety Inventory (BAI) ≥ 8	Mothers *N* = 200, *n* = 82 (42.1%)	Correlation	**Factors** (During NICU)		***r***			***P***
						**Postpartum depression**		**0.56**			** < 0.05**
						**IDAS—panic**		**0.78**			** < 0.001**
						**IDAS—trauma**		**0.66**			** < 0.001**
						**Anxiety Arousal**		**0.94**			** < 0.05**
						**Married**		**-0.17**			** < 0.05**
						Maternal age		-0.13			NS
						Caucasian		0.0			NS
						Education		-0.04			NS
						Income		-0.14			NS
						Employed		-0.02			NS
						Time in NICU		-0.01			NS
						**Depression history**		**0.43**			** < 0.05**
						**Infant illness**		**0.18**			** < 0.05**
						Infant birthweight		-0.04			NS
						GA		-0.06			NS
						Prematurity		-0.05			NS
**Shivhare 2022 **[79]	Mothers to NICU term and preterm infants	At one month postpartum	STAI > 40	Mothers *N* = 100, *n* = 23 (23%)	Differences between groups (STAI < 40 vs STAI > 40)	**Factors** (One month after birth)	**N**	**STAI < 40**	**STAI > 40**		***P***
						GA < 37 weeks GA ≥ 37 weeks	76	57 (57%)	19 (19%)		NS
							24	20 (20%)	4 (4%)		
						Baby’s feeding					
						Tube feeding Breast feeding	14	8 (8%)	6 (6%)		NS
							86	69 (69%)	17 (17%)		
						Caesarean delivery Vaginal delivery	37	29 (29%)	8 (8%)		NS
							63	48 (48%)	15 (15%)		
						NNU admission reasons					
						Medical conditions Observation Surgery	72	54 (54%)	18 (18%)		NS
							21	16 (16%)	5 (5%)		
							7	7 (7.0%)	0		
						Male baby Female baby	43	33 (55%)	10 (10%)		NS
							57	44 (44%)	13 (13%)		
						BW 2.5kg BW 1.5–2.4kg BW < 1.5kg	20	17 (17%)	3 (3%)		NS
							63	47 (47%)	16 (16%)		
							17	13 (13%)	4 (4%)		
						Apgar < 8 Apgar = 8 Apgar > 8	17	13 (13%)	4 (4%)		NS
							65	48 (48%)	17 (17%)		
							18	16 (16%)	2 (2%)		
						NICU stay 7–15 days NICU 16–30 days NICU > 30 days	52	38 (38%)	14 (14%)		NS
							37	30 (30%)	7 (7%)		
							11	9 (9%)	2 (2%)		
**Treyvaud 2016 **[71]	Multiples and bereaved families to infants < 30 wks and BW < 1250 g	2 and 7 years corrected age	HADS elevated anxiety 8–21, clinically significant anxiety HADS ≥ 11	Mothers *N* = 162 of (multiple 33, singleton 129) Multiple elevate *n* = 8 (31%) clinical anxiety 3 (12%), singleton *n* = 45 (44%), clinical anxiety 23 (23%)	Logistic regression	**Factors** (At 7 seven years corrected age)	**OR**		**95%CI**		***P***
						Multiple vs. singleton	0.56		0.22, 1.41		0.22
						Multiple vs. singleton (for clinically significant symptoms anxiety)	0.44		0.12, 1.62		0.22
						**Bereavement vs no bereavement (elevated anxiety) HADS 8–21**	**3.63**		**1.05, 12.5**		**0.04**
						Bereavement vs no bereavement (clinically significant anxiety) HADS 11–21	1.2		0.31, 4.72		0.79
**Vizcarrondo-Oppenheimer 2021 **[76]	Mothers to NICU infants	5–8 wks postpartum	HAM-A ≥ 14	Mothers *N* = 92, *n* = 18 (19.6%)	Regression	**Factors** (At 5–8 weeks postpartum)		**B**			***P***
						**Cumulative psychosocial risk factor** (perceived Stress 1 yes, 0 no, socio-economic status 1 semiskilled 0 major/minor profession and skilled worker, mother’s age 1 ≤ 20, 0 > 20)		**0.267**			**0.011**
						**Cumulative neonatal risk factor** (birth weight 1 ≤ 1500 g, 0 > 1500 g, gestational age 1 ≤ 32 weeks, 0 > 32 week, Apgar scores 1 ≤ 5, 0 > 5)		**-0.220**			**0.039**
						Obstetric risks (preeclampsia, high blood pressure and diabetes)		NR			NS
**Zanardo 1998 **[72]	Parents of high-risk premature twins vs singletons	pre-discharge	STAI, cut off = NR	Parents *N* = 55 (30 mothers, 25 fathers) Twins mothers 40 ± 9 state and 38 ± 9 trait, fathers 35 ± 8 state and 34 ± 6 trait	Mean difference (MD)	**Factors** (At NNU discharge)		**STAI State** **Mean difference (95%CI)**	**STAI Trait** **Mean difference (95%CI)**		
						Fathers vs mothers (twins)		*-5.0 (-11.42, 1.42)*	*-5.00 (-10.68, 0.68)*		
						Fathers vs mothers (singleton)		*-6.00 (-12.30, 0.30)*	*-4.00 (-9.56, 1.56)*		
						Twins mothers vs singleton mothers		*0.00 (-6.44, 6.44)*	*2.00 (-4.09, 8.09)*		
						Twins fathers vs singleton fathers		*1.00 (-5.28, 7.28)*	2.00 [-3.13, 7.13]		
		One month later		Twins mothers state 38 ± 9 and 36 ± 8 trait fathers state 33 ± 5 and 33 ± 7 trait	Mean difference (MD)	**Factors** (1 month post discharge**)**		**STAI State** **MD (95%CI)**	**STAI Trait** **MD (95%CI)**		
						Fathers vs mothers (twins)		*-5.00 (-10.36, 0.36)*	*-3.00 (-8.28, 2.28)*		
						**Fathers vs mothers** (singleton)		*-4.00 (-10.02, 2.02)*	*-4.00 (-9.93, 1.93)*		
						Mothers twins vs singleton		*1.00 (-5.81, 7.81)*	*0.00 (-5.73, 5.73)*		
						Fathers twins vs singleton		*0.00 [-4.35, 4.35]*	*0.00 [-5.13, 5.13]*		

*Abbreviations*: *95%CI* 95% confidence interval, *aOR* adjusted odd ratio, *aRR* adjusted Relative Risk, *ART* Assisted Reproduction Techniques, *BW* Birth weight, *CA* corrected age, *CRIB* Clinical Risk Index for Babies, *CS* Caesarean section, *CD-RISC* Connor-Davidson Resilience Scale, *EPDS* Edinburgh Postnatal Depression Scale, *HAM-A* Hamilton anxiety rating scale, *IDAS* Inventory of Depression and Anxiety Symptoms, *Metric 1* using only items that fathers indicate as a source of stress, and overall stress level, *Metric 2* using all the items, giving a rating of 1 (not stressful) to the items that were indicated as not experienced, *MTS* Munich Grief Scale, *NA* not applicable, *PEISM* Pictorial Representation of Illness and Self Measure, Problem-solving Skills Evaluation Form, *PSS* Parental Stress Scale, *PTGI* Post-traumatic Growth Inventory, (subscales are: ‘relating to others’, ‘new possibilities’, ‘personal strength’, ‘spiritual change’, and ‘appreciation of life’), *STAIS* State Anxiety Inventory scores, *Wks* Weeks

^a^Studies included in post-traumatic stress and anxiety: Garfield 2015, [27] Greene 2015 & 2019, [28, 29] Holditch-Davis 2009, [31] Lotterman 2018, [45] Misund 2013 & 2014, [36, 37] Moreyra 2021, [47] Pisoni 2022

^b^Severe maternal morbidity, composite endpoint defined as the occurrence of at least one of the following complications: severe postpartum haemorrhage defined by the use of a blood transfusion, *ICU* intensive care unit admission or death

^c^Severe neonatal morbidity, composite endpoint defined as any of the following outcomes: grades 3–4 *IVH* Intraventricular Haemorrhage or *ROP* Retinopathy of Prematurity or laser treatment, and/or severe bronchopulmonary dysplasia, intra parenchymal haemorrhage, *cPVL* Cystic Periventricular Leukomalacia, stages II and III *NEC* Necrotizing Entero-Colitis, stage 3 or higher

^d^Previous mental health conditions: Bipolar disorder, depression, generalized anxiety disorder, posttraumatic stress disorder

**Table 6:** Mapping of anxiety factors

Not statistically significant								**Factors**						Statistically significant		
Parent demographic factors																
Blanc 2021 [56] *n* = 2270	Damanabad 2019 [63] *n* = 100	Dantas 2012 [62] *n* = 70	Garfield 2015 [27] *n* = 113	Kong 2013 [74] *n* = 280	Park 2022 [79] *n* = 91	Rogers 2013 [69] *n* = 73	Segre 2014 [70] *n* = 200	age								
			Dantas 2012 [62] *n* = 70	Garfield 2015 [27] *n* = 113	Greene 2015 [28] *n* = 69	Park 2022 [79] *n* = 91	Segre 2014 [70] *n* = 200	education	Carvalho 2008 [61] *n* = 36	Damanabad 2019 [63] *n* = 100	Kong 2013 [74] *n* = 280					
			Buchi 2007 [58] *n* = 54	Dickinson 2022 [75] *n* = 114	Feeley 2007 [65] *n* = 122	Kong 2013 [74] *n* = 200	Zanard 1998 [72] *n* = 55	sex	Moreyra 2021 [47] *n* = 120	Mulder 2014 [68] *n* = 447						
					Blanc 2021 [56] *n* = 2270	Dantas 2012 [62] *n* = 70	Rogers 2013 [69] *n* = 73	couple’s relationship	Cajiao-Nieto 2021 [59] *n* = 51	Segre 2014 [70] *n* = 200						
				Damanabad 2019 [63] *n* = 100	Dantas 2012 [62] *n* = 70	Kong 2013 [74] *n* = 280	Segre 2014 [70] *n* = 200	family income	Cajiao-Nieto 2021 [59] *n* = 51	Greene 2015 [28] *n* = 69						
				Blanc 2021 [56] *n* = 2270	Damanabad 2019 [63] *n* = 100	Park 2022 [79] *n* = 91	Segre 2014 [70] *n* = 200	employment	Cajiao-Nieto 2021 [59] *n* = 51							
					Moreyra 2021 [47] *n* = 120	Rogers 2013 [69] *n* = 73	Segre 2014 [70] *n* = 200	ethnicity								
								area deprivation	Kong 2013 [74] *n* = 280	Okito 2022 [76] *n* = 45						
								medical insurance	Kong 2013 [74] *n* = 280							
							Rogers 2013 [69] *n* = 73	smoking								
								cumulative psychosocial risk factor	Vizcarrondo-Oppenheime 2021 [78], *n* = 92							
Pregnancy and birth factors																
					Blanc 2021 [56] *n* = 2270	Dantas 2012 [62] *n* = 70	Rogers 2013 [69] *n* = 73	parity	Carvalho 2008 [61] *n* = 36	Damanabad 2019 [63] *n* = 100	Cajiao-Nieto 2021 [59] *n* = 51	Misund 2014 [37] *n* = 29	Park 2022 [79] *n* = 91	Greene 2015 [28] *n* = 69	Rogers 2013 [69] *n* = 73	
						Cajiao-Nieto 2021 [59] *n* = 51	Park 2022 [79] *n* = 91	Assisted reproductive techniques								
			Blanc 2021 [56] *n* = 2270	Cajiao-Nieto 2021 [59] *n* = 51	Park 2022 [79] *n* = 91	Treyvaud 2016 [71] *n* = 162	Zanard 1998 [72] *n* = 55	multiple pregnancy								
							Dantas 2012 [62] *n* = 70	number of antenatal visits								
							Blanc 2021 [56] *n* = 2270	preeclampsia								
								Pregnancy complications	Dantas 2012 [62] *n* = 70							
							Vizcarrondo-Oppenheime 2021, [78] *n* = 92	cumulative obstetric risk score								
				Cajiao-Nieto 2021 [59] *n* = 51	Damanabad 2019 [63] *n* = 100	Dantas 2012 [62] *n* = 70	Shivhare 2022 [77] *n* = 100	Mode of birth	Blanc 2021 [56] *n* = 2270							
							Blanc 2021 [56] *n* = 2270	Premature birth								
								Antenatal infant health risk/congenital anomalies	Cajiao-Nieto 2021 [59] *n* = 51	Fontoura 2018 [66] *n* = 115						
							Blanc 2021 [56] *n* = 2270	meeting newborn								
							Blanc 2021 [56] *n* = 2270	skin to skin contact								
							Park 2022 [79] *n* = 91	postnatal care education								
							Cajiao-Nieto 2021 [59] *n* = 51	mothers’ length of hospitalisation								
							Blanc 2021 [56] *n* = 2270	severe maternal morbidity								
Infant demographic factors																
				Cajiao-Nieto 2021 [59] *n* = 51	Dantas 2012 [62] *n* = 70	Segre 2014 [70] *n* = 200	Shivhare 2022 [77] *n* = 100	gestational age GA	Damanabad 2019 [63] *n* = 100	Misund 2014 [37] *n* = 29	Park 2022 [79] *n* = 91					
		Cajiao-Nieto 2021 [59] *n* = 51	Damanabad 2019 [63] *n* = 100	Dantas 2012 [62] *n* = 70	Khemakhem 2020 [73] *n* = 10	Segre 2014 [70] *n* = 200	Shivhare 2022 [77] *n* = 100	birth weight BW	Carvalho 2008 [61] *n* = 36	Greene 2019 [29] *n* = 69						
							Segre 2014 [70] *n* = 200	prematurity								
					Dantas 2012 [62] *n* = 70	Khemakhem 2020 [73] *n* = 10	Shivhare 2022 [77] *n* = 100	Apgar scores								
					Damanabad 2019 [63] *n* = 100	Park 2022 [79] *n* = 91	Shivhare 2022 [77] *n* = 100	sex								
								cumulative neonatal risk factor	Vizcarrondo-Oppenheime 2021, [78] *n* = 92							
Infant health and care factors																
						Gennaro 1988 [67] *n* = 41	Okito 2022 [76] *n* = 45	clinicians’ perception of infant’s health	Carvalho 2008 [61] *n* = 36	Garfield 2015 [27] *n* = 113	Lotterman 2018 [45] *n* = 61	Pisoni 2020 [49] *n* = 29	Segre 2014 [70] *n* = 200			
							Lotterman 2018 [45] *n* = 91	Mother infant attachment/contact and bonding	Bonacquisti 2020 [57] *n* = 127							
							Lotterman 2018 [45] *n* = 91	number of NNU visits								
							Shivhare 2022 [77] *n* = 100	infant’s feeding								
							Lotterman 2018 [45] *n* = 91	participation in infant care	Cakmak 2018 [60] *n* = 340							
							Lotterman 2018 [45] *n* = 91	information seeking: technical questions and infant’s care								
							Lotterman 2018 [45] *n* = 91	interaction with healthcare professionals								
								information seeking: receiving and understanding	Lotterman 2018 [45] *n* = 91							
			Cajiao-Nieto 2021 [59] *n* = 51	Lotterman 2018 [45] *n* = 91	Rogers 2013 [69] *n* = 73	Segre 2014 [70] *n* = 200	Shivhare 2022 [77] *n* = 100	length of NNU stay	Carvalho 2008 [61] *n* = 36							
							Rogers 2013 [69] *n* = 73	infant’s brain injury								
								ventilation	Park 2022 [79] *n *= 91							
							Rogers 2013 [69] *n* = 73	number of days on a ventilator								
								oxygen treatment	Park 2022 [79] *n* = 91							
								antibiotic treatment	Park 2022 [79] *n* = 91							
							Blanc 2021 [56] *n* = 2270	severe neonatal morbidity								
							Pisoni 2020 [49] *n* = 29	infant’s developmental								
							Shivhare 2022 [77] *n* = 100	reasons for NNU admission								
							Blanc 2021 [56] *n* = 2270	type of neonatal room								
							Blanc 2021 [56] *n* = 2270	place discharged /transfer red to								
Parental history of mental health and trauma factors																
					Cajiao-Nieto 2021 [59] *n* = 51	Lotterman 2018 [45] *n* = 91	Rogers 2013 [69] *n* = 73	history of mental health								
								history of depression	Das 2021 [64] *n* = 96	Segre 2014 [70] *n* = 200						
							Cajiao-Nieto 2021 [59] *n* = 51	history of mental health family member								
							Rogers 2013 [69] *n* = 73	stressful life events								
							Segre 2014 [70] *n* = 200	panic s/trauma symptoms								
							Segre 2014 [70] *n* = 200	anxious arousal symptom								
Parental postnatal mental health factors																
								postpartum depression	Dantas 2012 [62] *n* = 70	Garfield 2015 [27] *n* = 113	Greene 2015 [28] *n* = 69	Holditch-Davis 2009 [31] *n* = 117	Khemakhem 2020 [73] *n* = 10	Moreyra 2021 [47] *n* = 120	Park 2022 [79] *n* = 91	Segre 2014 [70] *n* = 200
							Lotterman 2018 [45] *n* = 91	early anxiety								
								posttraumatic stress symptoms (PTS)	Garfield 2015 [27] *n* = 113	Moreyra 2021 [47] *n* = 120						
Parent stress, coping and support factors																
								stress: infant’s appearance and behaviour	Cajiao-Nieto 2021 [59] *n* = 51	Holditch-Davis 2009 [31] *n* = 117						
								stress: sights and sounds	Cajiao-Nieto 2021 [59] *n* = 51							
							Rogers 2013 [69] *n* = 73	stress: parental role alteration	Cajiao-Nieto 2021 [59] *n* = 51	Holditch-Davis 2009 [31] *n* = 117						
							Cajiao-Nieto 2021 [59] *n* = 51	stress: staff-behaviour and communication								
								stress: overall	Cajiao-Nieto 2021 [59] *n* = 51	Garfield 2015 [27] *n* = 113						
								forward coping style	Lotterman 2018 [45] *n* = 91							
							Lotterman 2018 [45] *n* = 91	optimism								
							Okito 2022 [76] *n* = 45	resilience								
								guilt feeling	Park 2022 [79] *n* = 91							
						Cajiao-Nieto 2021 *n* = 51	Okito 2022 [76] *n* = 45	social support	Rogers 2013 [69] *n* = 73							
Other factors																
								bereavement	Buchi 2007 [58] *n* = 54	Treyvaud 2016 [71] *n* = 162						
							Buchi 2007 [58] *n* = 54	grief/suffering								
								postpartum physical health	Park 2022 [79] *n* = 91							

*Abbreviations*: *Cumulative psychosocial risk factor* mother’s age, perceived stress and employment status, *Cumulative obstetric risk score* preeclampsia yes/no, high blood yes/no and diabetes yes/no, *Cumulative neonatal risk factor* summing birth weight, *gestational age* Apgar scores, *NNU* Neonatal unit, *Severe neonatal morbidity* defined as any of the following outcomes: grades 3–4 *IVH* IntraVentricular Hemorrhage, *ROP* Retinopathy Of Prematurity or laser treatment and/or severe bronchopulmonary dysplasia, intraparenchymal hemorrhage, *cPVL* cystic PeriVentricular Leukomalacia, *NEC stages II and III* Necrotizing EnteroColitis stage 3 or higher

- 1) Parent demographic factors (Ten factors: age, education, sex, ethnicity, parents’ area deprivation, income, employment status, housing and access to transport, single parent, family social risk).

Parental age was examined as a determinant of anxiety in eight studies [27, 56, 62, 63, 69, 70, 74, 79] and none showed any significant association with developing anxiety at any time point. The association between parental education and anxiety was reported in eight studies [27, 28, 61–63, 70, 74, 79]. In one study [61], parents’ educational level correlated negatively with anxiety scores and in another study [74], parents with low education levels had significantly higher anxiety scores. In contrast, in one study [63], mothers with university degrees had higher state anxiety scores after birth compared to mothers with a diploma or lower level of educational attainment. No evidence of association between elevated anxiety scores and parental factors was found in the remaining studies.

The association between sex of parents and anxiety was reported in seven studies [47, 58, 65, 68, 72, 74, 75]. In two studies [47, 68], fathers reported significantly fewer anxiety symptoms than mothers during NNU admission [47] and also at nine months [68]. In all other studies, no significant associations were found. Couple’s relationship was explored in five studies [56, 59, 62, 69, 70]. Being married was associated with lower anxiety scores during NNU admission in one study [70], yet marital status was not associated with anxiety symptoms in the other three studies [56, 62, 69]. A negative description of a couple’s relationship status was associated with greater anxiety scores in fathers two to three weeks after birth in one study [59].

The relationship between family income and anxiety was investigated in six studies [29, 59, 62, 63, 70, 74]. In one study, having a low income status was associated with lower anxiety scores amongst mothers at the time of birth [29]. In contrast, in another study, low family income was associated with elevated paternal anxiety symptom scores after birth [59]. In four studies [62, 63, 70, 74] family income was not significantly correlated with anxiety scores. Employment was explored in five studies [56, 59, 63, 70, 79]. An unemployed father was associated with elevated anxiety scores among fathers at birth in one study [59]. However, in the four other studies, no association was found between parents’ employment status and developing anxiety at birth [63], two to three weeks after birth [79], during NNU admission [70] or at NNU discharge [56]. Three studies [47, 69, 70], considered ethnicity as a factor relevant to anxiety during NNU stay and no significant association was found. Area deprivation was evaluated in relation to anxiety in two studies [74, 76]: parents residing in more economically advantaged areas were found to be 6.5 times more likely to report anxiety two weeks after birth during the NNU stay [76]. Anxiety scores during the first week after birth were lower among mothers living in an urban residential area compared to those living in a rural area [74], and the same study found higher anxiety scores among women without medical insurance compared to those who were insured [74]. One study explored smoking status and found that smoking was not significantly correlated with anxiety [69]. One study [78] looked at a cumulative psychosocial risk factor score, comprising younger maternal age, perceived stress and low socio-economic status),- and found this was significantly associated with greater maternal anxiety scores.

- 2) Pregnancy and birth factors (15 factors: parity, in-vitro fertilisation, multiple pregnancy, number of antenatal visits, preeclampsia, pregnancy complications, cumulative obstetric risks, mode of birth, preterm birth, infant health risk/congenital anomalies, timing of when parents met their newborn, skin to skin, postnatal care education, mother’s length of stay, maternal severe morbidity).

The association between parity and anxiety was explored in nine studies [28, 37, 56, 59, 61–63, 69, 79]. Being primiparous, a first time mother, was an independent risk factor associated with anxiety in four studies [28, 37, 59, 79]. Primiparous mothers had significantly higher anxiety scores than multiparous mothers at two to three weeks after birth [79]. Similarly, primiparous mothers were seven times more likely to report anxiety symptoms prior to NNU discharge [28]. Even when the assessment was at six to eighteen months post term, primiparity was still a significant risk factor for state anxiety [37]. However, being primiparous was not associated with state anxiety symptoms at NNU discharge in two studies [56, 69]. In two further studies, multiparous compared to nulliparous mothers exhibited higher state anxiety scores [61, 63]. Furthermore, mothers who had given birth three or more times had higher state and trait anxiety mean scores in one study [63]. No correlation was reported between number of children and anxiety during NNU stay or at discharge [62, 69]. Among fathers [59], a significant association was found between being a first time father and elevated state anxiety scores after birth. Assisted reproductive techniques [59, 79], multiple pregnancy [56, 59, 71, 72, 79], number of antenatal visits [62], and preeclampsia were not significant risk factors for state anxiety at NNU discharge [56]. However, pregnancy complications were significantly correlated with elevated state and trait anxiety scores during NNU stay [62]. A cumulative obstetric risk score comprising preeclampsia, high blood pressure and diabetes was not associated with higher anxiety scores in one study [78]. Mode of birth was considered in five studies [56, 59, 62, 63, 77]. Having a caesarean section after 26 weeks was associated with more state anxiety symptoms compared to spontaneous vaginal birth after 26 weeks, evidence from a large study that adjusted for neonatal birthweight, severe neonatal morbidity, maternal age, employment and parity [56]. No association was found in the remaining four studies [59, 62, 63, 77]. Preterm birth, either induced or spontaneous, was not associated with developing state anxiety at NNU discharge in one good quality study [56]. Two studies looked at the influence of receiving information about health risk or congenital anomaly in the foetus during antenatal scans on anxiety. Among fathers [59], infant health risks detected antenatally were a significant risk factor for anxiety at two–three weeks post birth. However, among mothers, trait anxiety was lower when baby’s diagnosis of congenital anomalies was made antenatally than postnatally [66]. Timing of when parents met their newborn [56], skin to skin contact, [56], receiving postnatal care education [79], mothers’ length of hospitalisation [59] and a composite factor of severe maternal morbidity [56] were not significantly associated with anxiety.

- 3) Infant demographic factors (Six factors: gestational age, birthweight, prematurity, Apgar score, sex, cumulative neonatal risk factor)

Gestation age was considered in seven studies [37, 59, 62, 63, 70, 77, 79]. Mothers to infants born at 33 weeks of gestational age or less experienced higher state anxiety at birth compared to mothers > 34 weeks [63] and at two to three weeks after birth [79]. This was consistent even at a later assessment at six and 18-month post-term age [37]. Among fathers, GA ≤ 28 week vs > 28 was not a significant factor associated with higher state anxiety scores after birth or 2–3 weeks later [59], nor was GA < 37 and ≥ 37 weeks among mothers [77]. Birth weight was reported in eight studies [29, 59, 61–63, 70, 73, 77]. Lower BW was significantly correlated with higher anxiety scores in one study [61]. Moreover, each 100 g increase in birthweight was associated with a two point decrease in maternal anxiety in another study [29]. Birthweight ≤ 1500g was not a significant factor among fathers [59]. During NNU, BW was not significantly /associated with anxiety in four studies [62, 70, 73, 77]. No statistically significant difference was found between BW and state anxiety mean scores [63]. Prematurity (an aggregate of infant BW and age) was not correlated with anxiety scores during NNU [70]. Apgar scores [62, 73, 77] and infant’s sex [63, 77, 79] were not significantly associated with anxiety. A cumulative neonatal risk factor, based on BW, GA and Apgar scores, was associated with a significant increase in anxiety scores in one study [78].

- 4) Infant health and care factors (19 factors: clinicians’ perception of infant’s health, mother-infant attachment and bonding, number of NNU visits, feeding, mothers’ participation in baby care, maternal question asking, interaction with health care professionals, mothers understanding of explanations, length of hospitalisation, brain injury, ventilation, number of days on a ventilator, oxygen treatment, antibiotic treatment, severe neonatal morbidity, development, NNU admission reasons,, NNU room, place discharged/transferred to).

Seven studies [27, 45, 49, 61, 67, 70, 76] examined the association between clinicians’ perception of infant’s health and parental anxiety. Clinicians’ perception of infant health was measured using varied scales, the clinical risk index for babies (CRIB) score in [61] and it was a significant risk factor for elevated maternal state anxiety. Similarly, health professional rating of the severity of the infant’s illness assessed via Neurobiologic Risk Score (NBRS) in [27] was significantly correlated with maternal state anxiety at three months after birth. The influence of infant health status on parental anxiety was apparent during NNU stay in three studies [45, 49, 70]. Presence of an infant health problem was a predictor only at first assessment during NNU [45]. Whereas infant perinatal risk status using Perinatal Risk Inventory (PERI) correlated significantly with state anxiety during NNU assessment in [49], and infant illness severity was significantly correlated with anxiety during NNU stay [70]. Infant health determined using the neonatal risk categorisations by [80] was not associated with anxiety one week after birth [67]. Severity of the infant’s condition was not associated with elevated anxiety during NICU stay at two weeks after birth [76]. Maternal-infant attachment/contact and bonding (physical and verbal) while in NNU was negatively correlated with anxiety symptoms in [57], but no significant association was found in Lotterman et.al., nor was the number of NNU visits per week, at assessment six months later [45]. Infant feeding, tube or breast was not significantly associated with more anxiety symptoms in [77]. Mothers’ participation in infant care while in NNU was reported on in two studies [45, 60]. The participation of mothers in many aspects of baby care resulted in reducing state and trait anxiety scores only in [60]. In [45] mothers seeking information and asking technical questions e.g. about the equipment and questions related to baby care, and having a positive relationship with healthcare professionals reduced anxiety scores at NNU but the effect was not significant at six months assessment. Whereas, mothers’ receiving explanations from NNU healthcare providers about treatment procedures and infant’s care and being able to understand were perceived as a calming anxiety factor.

Infant length of stay was reported in six studies [45, 59, 61, 69, 70, 77]. A longer hospital stay was correlated with state and trait anxiety scores in one study [61]. No correlation was found in the remaining studies. Severity of infant brain injury was not correlated with anxiety scores [69]. One study found that mothers’ to infants who required ventilation had significantly higher anxiety scores [79]. However the number of days on a ventilator was not correlated significantly with anxiety in another [69]. Both oxygen treatment and antibiotic treatment were associated with higher anxiety scores in [79], but severe neonatal morbidity was not associated with higher state or trait anxiety scores at NNU discharge in another study [56]. Infant development score using a Generalised Developmental Quotient (GQ) [81] at one year was not correlated with state or trait anxiety in one study [49].

The reasons for NNU admission, whether it was surgical, medical or for observation, was not associated with more anxiety symptoms at one month post-birth [77]. Type of neonatal room, whether single or multiple, was not associated with more trait anxiety symptoms [56]. Place discharged to, home or transfer to another hospital, was not a significant risk factor for increased state anxiety symptoms [56], whereas being in hospital rather than discharged home was significantly associated with higher anxiety scores [79].

- 5) Parental history of mental health problems and trauma factors (Six factors: history of mental condition, history of depression, history of mental health condition any family member, stressful life events, panic and trauma, anxious arousal symptoms).

Three studies [45, 59, 69] reported on the association between parental history of mental health conditions, eg. depression, anxiety or bipolar disorders and developing anxiety, none reported any significant association. Two studies [64, 70] looked at the impact of a previous history of depression and both reported a significant correlation with anxiety scores during NNU. A history of mental health problems of any family member and anxiety scores was reported on in [59] and no significant association with anxiety was found. Stressful life events were not correlated with anxiety scores at NNU discharge in [69], however, panic and trauma symptoms were significantly correlated with anxiety [70]. Similarly anxious arousal, a composite variable for panic, was significantly correlated with anxiety [70].

- 6) Parental postnatal mental health factors (Three factors: postpartum depression, posttraumatic stress symptoms (PTS), persistent anxiety)

Postpartum depression was reported in eight studies [27, 28, 31, 47, 62, 70, 73, 79] all of which found a significant association with anxiety. Postpartum depressive symptoms after birth significantly increased the odds of developing anxiety prior to discharge [28]. Also, depressive symptoms correlated significantly with state anxiety scores during NNU [31, 62], prior to NNU discharge, at two to three weeks after birth [79], and at three months after birth [27]. Anxiety symptoms and postpartum depression were significantly correlated during NNU stay [70, 73]. Two studies looked at PTS and found a significant correlation between postpartum depression and state anxiety/anxiety [27, 47]. Early anxiety symptoms during NNU were not a predictor for anxiety six months later [45].

- 7) Parent stress, coping and support factors (Ten factors: Infant’s appearance of stress, stressful sights and sounds, parental role alterations, staff behaviour and communication, PSS scores, coping style, optimism, parents’ resilience, guilt feeling, social support).

Parental stress was measured by Parental Stressor Scale: NICU (PSS: NICU) [54] in four studies [27, 31, 59, 69], one further study [59] reported on PSS four subscales. Infant’s appearance and behaviour stress subscale was reported on in two studies [31, 59] and it was found to be a significant stressor for state anxiety in both**:** In [59] after birth and at 2–3 weeks afterwards, and in [31] during NNU assessment. Stressful sights and sounds in NNU were associated with state anxiety only at first assessment after birth in [59]. Parental role alterations stress (e.g. not being able to feed the infant) in the NNU was a significant factor of more state anxiety symptoms after birth and two to three weeks afterwards in [59] and during NNU [31]. In contrast, no correlation was found in [69]. Stress related to staff-behaviour and communication was not correlated with fathers’ anxiety scores [59]. Total PSS score measuring overall parental stress was directly correlated with state anxiety at three months after birth in [27]. Furthermore, elevated PSS scores was associated with higher state anxiety scores in [59].

The relationship between maternal coping strategies and anxiety level was examined in one study [45]. Forward coping style was associated with lower anxiety scores during NNU [45]. There was no significant association between maternal optimism about the infant’s recovery and anxiety scores at six months [45]. Parents’ resilience was not a risk factor for anxiety during NICU stay at two weeks post birth [76]. Mothers’ feeling of guilt scores, based on scores for fault, responsibility, punishment, and feelings of helplessness, was significantly correlated with state anxiety scores at two to three weeks after birth [79].

Fathers’ perception of social support and satisfaction with social support were significantly associated with reduced state anxiety scores soon after birth, but not at a later assessment two to three weeks afterwards [59]. Similarly, maternal satisfaction with social support was not correlated with anxiety scores at NNU discharge [69]. How parents perceived social support was not an independent anxiety risk factor during the first two weeks of NNU stay [76].

- 8) Other factors (Three factors: bereavement, grief and suffering, maternal physical health)

Two studies [58, 71] included parents who experienced bereavement. All parents were bereaved in [58] and in [71] bereaved parents were compared to non-bereaved parents. There was a significant correlation between bereavement and anxiety scores among mothers but not fathers [58]. Bereaved families showed more anxiety symptoms than non-bereaved families even at seven years corrected age [71], yet no difference was found regarding clinically diagnosed anxiety between the two groups.

The grief/suffering scores at two to six years after the loss of the baby were not significantly correlated with anxiety [58]. Maternal physical health such as fatigue and shoulder pain were significantly correlated with state anxiety at two to three weeks after birth in [79].

## Discussion

This review is the first to systematically synthesise factors associated with PTS and anxiety symptoms among parents of infants admitted to NNU. There was significant methodological variability across the 49 included studies, involving 8,447 parents. This was due to differences in study design, inclusion criteria, timing of assessment, measuring tools and cut-off values used. There was also vast variations in defining and reporting on similar factors across the included studies. Therefore, the findings were synthesised narratively.

Although the majority of the identified factors were based on one or two small studies, several factors emerged from multiple studies that could allow healthcare professionals to determine which of these parents require more attention, early screening, referral and intervention before developing PTS and anxiety. Healthcare professionals should target those parents with previous diagnoses of mental health problems before pregnancy and parents who develop any mental health conditions during antenatal and postnatal periods. As seen in the general perinatal population, anxiety, depression and PTS are all highly comorbid conditions among parents of NNU infants [82–85].

Factors specific to this population associated with PTS or anxiety included preterm birth (≤ 33 weeks), having an extremely low birthweight (< 1000 g) infant and stressors in the NNU environment, in particular the infants’ appearance and behaviour. Unexpectedly, a number of factors specific to this group of parents showed no association with PTS or anxiety, such as reasons for NNU admission, severe neonatal morbidity, ventilation duration and number of NNU visits, although these results should be interpreted with caution because each of the findings were based on a single study.

A factor consistently found to be associated with PTS was the parents’ own perception of the severity of the infant’s illness. Also when staff did not convey information clearly, it caused emotional stress to the parents and left them feeling powerless and excluded [86]. In addition to good communication, active parent involvement in baby’s care while in NNU is a protective factor found in this review to reduce anxiety, as parents felt more comfortable and prepared to care for their baby after discharge [87] thus enhancing the long term positive impacts for the whole family [88]. Other studies have found fewer medical interventions to the infant and better infant development was seen after parents making physical contact with their infant while in NNU [89]. Also participation of parents in baby’s care during NICU, as part of a family centred intervention, was associated with a positive impact on infant’s clinical outcomes and a shorter NNU stay as reported in [90]. Overall, our findings highlight that protective factors around the care provided include communicating well with parents, asking about their perception of how ill their infant is and involving them in providing care to their infants.

Another protective factor for both PTS and anxiety was positive coping mechanisms used by parents after unexpected NNU admission. Encouraging parents to utilise positive coping or adapt their coping styles, such as by taking ‘time out’ or ‘debriefing’ when things go wrong, could be effective in improving their state of mental health and well-being [86, 87]. Parents’ degree of greater perceived social support and having a functioning social support setup also emerged as a protective factor for PTS therefore having wider targeted family psychological support and peer to peer support networks could have a positive impact on parents’ mental health [86]. Early engagement of peer-to-peer support during NNU stay and beyond discharge has also been found to be effective in improving stress, anxiety and depression symptoms [91, 92].

### Implications for practice and research

As with the general perinatal population a history of mental health problems and having co-morbid mental health conditions are factors associated with both anxiety and PTS in parents of babies admitted to NNU. Therefore, early screening of all parents for mental health problems is the best way to provide much needed information on which parents may need psychological support. As with the general perinatal population, increased awareness amongst healthcare professionals of the influence of history of mental health and co-morbid mental health problems is important in understanding the mental health needs of parents. Parent’s perception of infant illness is a distinctive factor for this group. Therefore clear communication, enquiring into the parents’ perception of their infant’s illness, and early participation of parents in the care of their babies may also ease symptoms of anxiety and PTS.

Bereaved parents are an important subgroup of NNU parents on which there were little data, therefore exploring the needs of these parents while in NNU and their long term comprehensive psychosocial support needs after the loss of the infant is crucial. Another major gap in the literature is the mental health of the fathers and non-traditional family models such as a single parent and same sex families of infants admitted to NNU. Future research should ensure fathers and parents of non-traditional families are included to better understand partner risk factors for PTS and anxiety, and how maternal and partner risk factors interact. Additional research targeting younger parents and those from different ethnic backgrounds is also needed, as are studies in low and middle income settings.

Improving the methodological rigor and standardising approaches to measurement of common mental health problems would add significantly to the current literature. For example, consensus between researchers on the tools and cut-off points for this population is needed. Expanding the scope of routine data collection to include parental risk factors, and linking to existing routine maternity/ primary care data sets would provide population level data to explore parent mental health risk factors more robustly. As many psychosocial risk factors are not routinely collected, a large, more population-based cohort study that additionally includes parents whose babies did not require NNU admission would help better predict the most at risk groups.

### Strengths and limitations

The review is a rigorous, comprehensive synthesis of an important research area. We adopted broad, inclusive eligibility criteria and followed a transparent research approach in line with the PRISMA 2020 recommendation. Robust risk of bias assessment and reporting on multivariable analysis, when available, helped reduce the risk of bias in reporting and interpretation of findings. A large number of studies were included in the review, however, despite comprehensive searching and contacting authors, the full texts from 11 studies could not be retrieved. It was not possible to separate out ASD and PTSD in the review as studies collected data over different time periods and often did not differentiate the one month cut-off for ASD. Meta-analysis of data was not feasible as many of the risk factors were examined in one study only, and where more than one study described a risk factor there was considerable methodological heterogeneity in study design, analysis, confounder factors and reporting, combined with clinical heterogeneity, different measures/cut-off points and variation in assessment time. While the data were too heterogeneous to meta-analyse, visually mapping the evidence provides an informative summary of the magnitude of data available for each risk factor.

## Conclusion

There is insufficient evidence to support a targeted approach to identifying parents at risk of developing anxiety and PTS when their baby is admitted to NNU. As with the general perinatal population, previous mental health and current co-morbid depression are risk factors for anxiety and PTS. Taking time to communicate well with parents and understand their perceptions of infants’ health may protect parents from experiencing anxiety and PTS symptoms. More research is needed to understand the impact of the NNU environment on parents’ mental health and also the association of low birth weight and a shorter gestational age with anxiety and PTS symptoms. There is some evidence, albeit limited, to suggest that engaging parents early in baby’s care and providing adequate social support may benefit the parents’ mental health. In the absence of evidence to support a targeted approach, routine screening for PTS and anxiety should be offered to all parents, even though, the optimal screening tool and the best administration time are not yet well established for this population.

### Supplementary Information

**Supplementary Material 1.**

**Supplementary Material 2.**

**Supplementary Material 3.**

## Data Availability

All data generated during this study are included in this published article and its supplementary information files.
