# Supplementary material for: Factors associated with posttraumatic stress and anxiety among the parents of babies admitted to neonatal care: a systematic review
Source: BMC Pregnancy Childbirth. 2024 May 9;24:352. doi: 10.1186/s12884-024-06383-5 (PMC11084127; doi:10.1186/s12884-024-06383-5)
Supplement: Supplementary file 1 — Supplementary Material 1. [file 12884_2024_6383_MOESM1_ESM.docx]

**Appendix 1: Medline search strategy**

1 Stress Disorders, Post-Traumatic/

2 Anxiety/ or exp Anxiety Disorders/

3 Stress, Psychological/

4 sleep disorders, intrinsic/ or "sleep initiation and maintenance disorders"/

5 Fatigue/

6 (posttraumatic or post-traumatic or ptsd or trauma*).mp.

7 (anxiet* or anxious).mp.

8 (phobia? or phobic).mp.

9 (obsessive compulsive disorder* or ocd).mp.

10 (((psycholog* or mental or acute) adj2 stress*) or stressful).mp. or stress*.ti.

11 (insomnia or sleepless* or ((problem? or disturbed or disrupt* or difficult*) adj2 sleep*)).mp.

12 (fatigue* or tired*).mp.

13 ((panic adj2 (attack? or disorder?)) or palpitation?).mp.

14 (fear? or worry or worries or concern? or crisis or crises).mp.

15 1 or 2 or 3 or 4 or 5 or 6 or 7 or 8 or 9 or 10 or 11 or 12 or 13 or 14

16 exp Parents/

17 Caregivers/

18 (parent or parents or father* or mother* or mums or moms or dads or maternal or paternal or carer? or caregiver? or care giver? or caretaker? or care taker? or guardian?).mp.

19 16 or 17 or 18

20 Intensive Care Units, Neonatal/

21 Infant, Newborn/ and (Intensive Care Units/ or Critical Care/)

22 exp infant, low birth weight/ or exp infant, premature/

23 ((infan* or neonat* or newborn? or baby or babies or preterm) adj5 (intensive care or intensive therap* or critical care or unit?)).ti,ab.

24 ((high dependency or special care) adj5 (infan* or neonat* or newborn? or baby or babies)).mp.

25 (nicu or scbu).ti,ab.

26 ((infan* or neonat* or newborn? or baby or babies) and (prematur* or preterm or low birth weight or "small for gestational age" or vlbw or lbw or sga)).ti.

27 20 or 21 or 22 or 23 or 24 or 25 or 26

28 Epidemiologic studies/

29 exp case control studies/

30 exp cohort studies/

31 Case control.tw.

32 (cohort adj (study or studies)).tw.

33 Cohort analy$.tw.

34 (Follow up adj (study or studies)).tw.

35 (longitudinal or retrospective or prospective).tw.

36 cross sectional.tw.

37 Cross-sectional studies/

38 28 or 29 or 30 or 31 or 32 or 33 or 34 or 35 or 36 or 37

39 15 and 19 and 27 and 38
