# Supplementary material for: Factors associated with posttraumatic stress and anxiety among the parents of babies admitted to neonatal care: a systematic review
Source: BMC Pregnancy Childbirth. 2024 May 9;24:352. doi: 10.1186/s12884-024-06383-5 (PMC11084127; doi:10.1186/s12884-024-06383-5)
Supplement: Supplementary file 2 — Supplementary Material 2. [file 12884_2024_6383_MOESM2_ESM.docx]

| **Appendix 2: Risk of bias of post-traumatic (PTS) included studies** | | | | | | | |
| --- | --- | --- | --- | --- | --- | --- | --- |
| **Study ID** | **Selection**  **bias** | **Sample size**  **bias** | **Factors assessment**  **bias** | **Outcome measurement bias** | **Analysis bias** | **Reporting bias** | **Attrition bias** |
|  | Were participants representative of the population that they were aiming to study in the included studies? | Does the study include enough participants to draw valid conclusions? | Were valid assessment tools used to measure the associated factors? | Diagnosis/  Assessment of PTSD using clinical assessment or validated measure | Were potential confounders adjusted for in the analysis? | Were all data relevant to the factors listed in the study fully reported? | What was the rate of the participants’ dropout during the study period? |
|  | **Low**  All participants included without applying any exclusion criteria, or applying a well justified exclusion criteria and the potential participants have an equal chance of sampling? | **Low**  Power calculation performed OR sample size were justified or any information on how the size sample was selected? | **Low**  All subjective factors assessed using a validated measure and objective factors were measured in the same way for all participants | **Low**  PTS clinically diagnosed OR self-reported using a validated tool | **Low**  Including a strategy for variable selection, unadjusted univariable and multivariable adjusted analysis reported | **Low**  Evidence that the study reports all planned analyses compared to analysis methods | **Low**  Participation rate at least 50% in cross-sectional studies or <20% lost from the initial sample in cohort studies OR the study reporting no differences between completers /enrolled and non-completers/non-enrolled |
|  | **Unclear**  No information | **Unclear**  No information | **Unclear**  No information | **Unclear**  No information | **Unclear**  No information | **Unclear**  No information | **Unclear**  No information |
|  | **High**  The study has applied a number of exclusion criteria | **High**  Concerns that study sample size is inadequate in relation to the number of factors | **High**  Self-reported subjective measures | **High**  Using not valid tools | **High**  No proper analysis strategy and only unadjusted analysis was reported | **High**  Concern that study has not reported the results of all analyses | **High**  High rate of participants dropout or low rate of the total sample included in analysis |
| **Anchan 2021** | **High**  Some parents were excluded; not English speakers, unable to maintain a follow-up assessment within the military healthcare system and using a convenience sampling strategy | **Low**  125 participants | **Low**  Factors assessed using valid measures including the infant’s illness severity | **Low**  Self-reported with a cut-off point | **Low**  Multivariate logistic regression | **Low**  All reported | **High**  27% lost |
| **Brunson 2021** | **High**  Some participants were excluded, acute/chronic psychological illness, drug/alcohol abuse, underage, and/ or with difficulties understanding or speaking French.  No actual information on the sampling method, but participants were included from 3 centres at certain period/convenience sampling | **High**  50 participants | **Low**  Factors assessed using valid measure and medical records | **Low**  Self-reported with a cut-off point | **High**  Univariate parametric tests | **Low**  All reported | **High**  50% lost |
| **Chang 2016** | **High**  Participants who did not speak or understand Chinese excluded and infants with congenital chromosomal abnormalities or congenital defects, cardiac surgery, infants who died excluded. A convenience sampling strategy was used | **Low**  102 participants | **Low**  Factors assessed using valid tools | **Low**  Self-reported with a cut-off point | **Low**  Multivariable logistic regression | **Low**  All reported | **High**  Cross sectional study, response rate 24%, identified 425 mothers  and 102 enrolled |
| **Clark 2021** | **High**  Age <18 years, infants died within the past 3 months, not speaking English were excluded. No information on the sampling strategy | **High**  67 participants | **Low**  Valid measures used for assessing associated factors | **Low**  Self-reported with a cut-off point | **Low**  Multivariable hierarchical linear regression | **Low**  All data reported | **High**  434 eligible parents and 67 (18%) completed surveys |
| **Eutrope 2014^a^** | **High**  Psychiatric illness, drug or alcohol abuse, aged <18 years, language barriers were excluded. A convenience sampling strategy was used | **Low**  100 participants | **Low**  Valid measures used for assessing relevant factors | **Low**  Self-reported with a cut-off point | **High**  Correlation | **Low**  All data reported | **Low**  100 mothers at visit 1 and 93 mothers at visit 2 |
| **Garfield 2015^a^** | **High**  Some participants were excluded. A study was part of a larger  randomised clinical trial | **Low**  113 participants | **Low**  Valid measure used to assess relevant factors | **Low**  Self-reported with a cut-off point | **High**  Correlation | **Low**  All reported | **Low**  All approached included in the analysis |
| **Greene 2015 & 2019^a^** | **High**  Younger mother, babies unlikely to survive were excluded.  A convenience sample from a larger study | **High**  69 participants | **Low**  Valid measure used to assess relevant factors | **Low**  Self-reported with a cut-off point | **Low**  Multivariable logistic regression | **Low**  All data reported | **Low**  100 eligible mothers and 69 (69%) included |
| **Hawthorne 2016** | **High**  Participants not speaking English/Spanish, multiples, being a foster were all excluded. No information on the sampling strategy | **Low**  165 participants | **Low**  Valid scale used for religious and spiritual activities | **Low**  Self-reported measure, no cut-off point | **Low**  Multivariable linear regression | **Low**  Multivariable regression | **Low**  All participants included in the analysis |
| **Holditch-Davis 2009^a^** | **High**  Congenital anomalies, HIV+, drug users were all excluded  A convenience sampling strategy was used | **Low**  177 participants | **Low**  Valid measure used to assess the relevant factors | **Low**  Self-reported with a cut-off point | **High**  Correlation | **Low**  All reported | **High**  177 enrolled 105 (60%) assessed |
| **Jubinville 2012** | **High**  Some were exclude e.g. Foetal anomaly and severe maternal illness. A convenience sampling strategy was used | **High**  40 participants | **Low**  Valid measure used to assess relevant factors | **Low**  Self-reported with a cut-off and clinical diagnosis | **High**  Correlation | **Low**  All reported | **Low**  First assessment 40 and second one 34 mothers only 15% lost |
| **Kim 2015** | **High**  Participants who did not speak Korean were excluded.  A convenience sample strategy was used | **Low**  113 participants | **Low**  Valid measure used to assess relevant factors | **Low**  Self-reported with a cut-off point | **Low**  Multivariable logistic regression | **Low**  All reported | **Low**  Out of 130 mothers, missing data at subsequent assessments were  <20% |
| **Lefkowitz 2010** | **High**  Inability to read English, parent age <18, babies who were less likely to survive were excluded.  A convenience sampling strategy was used | **Low**  102 participants | **Low**  Valid measure used to assess relevant factors | **Low**  Self-reported with a cut-off point | **Low**  Correlation and Multivariable linear regression | **Low**  All reported | **Low**  No significant differences  between enrolled and not enrolled families |
| **Lotterman 2018^a^** | **High**  Did not include mothers who were unable to visit  NICU or fathers. A convenience sample strategy was used | **High**  91 participants | **Low**  Valid measure used to assess relevant factors | **Low**  Self-reported with a cut-off | **Low**  Multivariable linear regression | **Low**  All reported | **Low**  Response rate 84% at time 2 |
| **Malin 2020 & 2022** | **High**  Participants who did speak English and parents to infants who were less likely to survive excluded.  A convenience sample strategy was used | **Low**  245 participants included and the sample size calculation was reported | **Low**  Valid measure used to assess relevant factors | **Low**  Self-reported with a cut-off point | **Low**  Univariate and multivariate logistic regression | **Low**  All reported | **Low**  Of the 194 parent-infant dyads eligible for follow-up, 167 (86%) completed three-month follow up |
| **Misund 2013 & 2014^a^** | **High**  Mothers of severely ill babies and non-Norwegian speakers were excluded. A convenience sampling strategy was used | **High**  29 participants | **Low**  Valid measures used to assess relevant factors | **Low**  Self-reported with a cut-off point | **Low**  Multivariable linear regression | **Low**  All reported | **Low**  29 of 34 families (85.3%) that met the inclusion  criteria were included |
| **Moreyra 2021^a^** | **High**  None were excluded, but  no information on the sampling strategy was provided | **Low**  150 participants | **Low**  Valid measures used to assess relevant factors | **Low**  Self-reported with a cut-off point | **High**  Comparison between parents and correlation analysis | **High**  Ethnicity data not reported | **Low**  158 participants eligible and 150 included |
| **Naeem 2019** | **High**  Some exclusions applied,  Convenience sampling | **Low**  160 participants and a sample size power calculation was performed | **Low**  Valid measures used to assess relevant factors | **Low**  Self-reported with a cut-off point | **High**  Non pragmatic test | **Low**  Only P values reported because it is non-parametric tests | **Low**  All included in the analysis |
| **Pace 2020** | **High**  Not speaking English excluded.  Sampling from larger Study | **Low**  105 participants | **Low**  Valid measures used to assess relevant factors | **Low**  Self-reported with a cut-off point | **Low**  Multivariable hierarchical logistic regression | **Low**  All reported | **Low**  No differences between included and not included parents |
| **Pisoni 2020^a^** | **High**  Some excluded, no random selection | **High**  29 participants | **Low**  Valid measures used to assess relevant factors | **Low**  Self-reported with a cut-off point | **High**  Correlation | **Low**  All reported | **Low**  All 29 included at both time points |
| **Rodriguez 2020** | **High**  Mothers with psychiatric  disorders before and/or during gestation,  babies with chronic conditions  & congenital excluded.  No information on sampling | **Low**  146 participants | **Low**  Valid measures used to assess relevant factors | **Low**  Self-reported with a cut-off point | **High**  Mann-Whitney/ Kruskal-Wallis test data was calculated | **High**  Not all data fully reported | **Low**  172 mothers, 146 included (85%) |
| **Salomè 2022** | **High**  No information on who were excluded and a convenience sampling strategy was used | **High**  20 couples | **Low**  Valid measure used to assess relevant factors | **Low**  Self-reported with a cut-off point | **Low**  Correlation then Multivariable regression | **Low**  All reported | **High**  32 invited and 20 included 62% |
| **Sharp 2020** | **High**  Some excluded not speaking English and younger mothers and a  convenience sampling strategy | **Low**  100 participants | **Low**  Valid measure used to assess relevant factors | **Low**  Self-reported with a cut-off point | **High**  Linear regression no adjustment | **Low**  All reported | **Low**  No differences in demographics, obstetrics, or outcomes between mothers included and the 14 mothers who were excluded |
| **Shaw 2009** | **High**  Not English speakers excluded and participants were part of another study | **High**  25 participants | **Low**  Valid measure used to assess relevant factors | **Low**  Self-reported with a cut-off point | **High**  Correlation | **Low**  All reported | **High**  Of the 40 participants who completed the baseline measures, 18 (45%) completed the follow-up measures |
| **Vinall 2018** | **High**  Parents to babies with congenital anomalies and required surgeries excluded. Part of an RCT | **High**  36 participants | **Low**  Valid measure used to assess relevant factors | **Low**  Self-reported with a cut-off point | **Low**  Multivariable linear regression | **Low**  All reported | **Low**  No difference between enrolled and excluded |
| **Williams 2021** | **High**  Not speaking English excluded, no information on the sampling strategy | **Low**  119 participants, a sample size calculation was performed | **Low**  Valid measure used to assess relevant factors | **Low**  Self-reported with a cut-off point | **High**  Correlations | **Low**  All reported | **Low**  Of the eligible mothers, 96% enrolled |

**Abbreviations: ^a^** Studies included in both posttraumatic stress and anxiety: Garfield 2015, Greene 2015 & 2019, Holditch-Davis 2009, Lotterman 2018, Moreyra 2021, Misund 2013 &2014, Pisoni 2022
