# Supplementary material for: Factors associated with posttraumatic stress and anxiety among the parents of babies admitted to neonatal care: a systematic review
Source: BMC Pregnancy Childbirth. 2024 May 9;24:352. doi: 10.1186/s12884-024-06383-5 (PMC11084127; doi:10.1186/s12884-024-06383-5)
Supplement: Supplementary file 3 — Supplementary Material 3. [file 12884_2024_6383_MOESM3_ESM.docx]

| **Appendix 3: Risk of bias of anxiety included studies** | | | | | | | |
| --- | --- | --- | --- | --- | --- | --- | --- |
| **Study ID** | **Selection bias** | **Sample size bias** | **Factors assessment**  **bias** | **Outcome measurement bias** | **Analysis bias** | **Reporting bias** | **Attrition bias** |
|  | Were participants representative of the population that they were aiming to study in the included studies? | Does the study include enough participants to draw valid conclusions? | Were valid assessment tools used to measure the presence of the associated factors? | Diagnosis/  Assessment of anxiety using clinical assessment or self reported validated measure | Were potential confounders adjusted for in the analysis? | Were all relevant factors data/analyses reported on? | What was the rate of participants’ dropout during the study period? |
|  | **Low**  All participants included without applying any exclusion criteria or well-justified exclusion criteria, potential participants have an equal chance of sampling? | **Low**  Power calculation performed, sample size was justified, or any information on how the sample size was selected? | **Low**  All subjective factors assessed using validated measures and objective factors were measured in the same way for all participants | **Low**  Anxiety clinically diagnosed or self-reported using a validated tool | **Low**  When the analysis method includes a clear strategy for variable selection, unadjusted univariable and multivariable adjusted analysis | **Low**  Evidence that the study reports all planned analyses compared to the analysis method | **Low**  Participation response rate at least 50% in cross-sectional studies or <20% lost from the initial sample in cohort studies OR the study reporting no differences between completers/enrolled and non-completers/non-enrolled |
|  | **Unclear**  No information | **Unclear**  No information | **Unclear**  No information | **Unclear**  No information | **Unclear**  No information | **Unclear**  No information | **Unclear**  No information |
|  | **High**  The study has applied a number of exclusion criteria | **High**  Concerns that study sample size is inadequate in relation to the number of factors | **High**  Self-reported subjective measures used | **High**  Using not valid tools | **High**  No proper analysis strategy and only unadjusted analysis was reported | **High**  Concern that the study has not reported the results of all analyses | **High**  High rate of participants dropout or low rate of the total sample included in analysis |
| **Blanc 2021** | **Low**  Population-based study and a random selection of participants | **Low**  2270 participants | **Low**  Valid measures used to assess all the factors | **Low**  Self-reported with a cut-off point | **Low**  Multivariable adjusted regression | **Low**  All reported | **Low**  All included in the analysis |
| **Bonacquisti 2020** | **High**  Fathers excluded, no information on sampling strategy | **Low**  127 participants | **Low**  Valid measures used | **Low**  Self-reported with a cut-off point | **High**  Correlation | **Low**  All reported | **High**  Included 127 and only 72 (56%) at the second assessment |
| **Buchi 2007** | **High**  Not speaking German excluded, sample was drawn from all parents to infants born during the study period | **High**  54 participants | **Low**  Valid measures used | **Low**  Self-reported with a cut-off point | **High**  Correlation | **Low**  All reported | **Low**  72 eligible parents (36 couples) 54 filled in and returned the questionnaire (response rate 75%) |
| **Cajiao-Nieto 2021** | **High**  Not speaking Spanish excluded and a convenience sampling strategy was used | **High**  51 participants | **Low**  Valid measures used for all factors | **Low**  Self-reported no cut-off point | **Low**  Bivariate adjusted for time of assessment | **Low**  All reported | **Low**  All included |
| **Cakmak 2018** | **High**  Parents to infants having contagious infection, mothers with physical disability/psychiatric condition excluded and a convenience sampling technique was used | **Low**  340 participants | **Low**  Valid measures used | **Low**  Self-reported with a cut-off point | **High**  Correlation | **Low**  All reported | **Low**  100% response rate |
| **Carvalho 2008** | **High**  Psychiatric history, human immunodeficiency virus (HIV), maternal  hospitalisation in intensive care excluded. A convenience sample strategy used | **High**  36 participants | **Low**  Validated measures used | **Low**  Standard tool | **High**  Correlation | **Low**  All reported | **High**  A sample loss of (n = 16) 44% |
| **Damanabad 2019** | **High**  Some excluded: severe obstetric complications and transferred to another hospital  For babies congenital abnormality, baby died in the first 24 hours, a convenient sampling method within a 5 month period was used | **Low**  100 participants, a sample size power calculation was performed | **Low**  Valid measures used | **Low**  Use of standardised instrument | **High**  Analysis of variance (ANOVA) | **High**  Data for not significant factors were not reported | **Low**  All included in the analysis |
| **Dantas 2012** | **High**  Some excluded: mothers to newborns who died, or with congenital anomaly, drug user, HIV+ and mental health illness and a convince sample strategy used | **High**  70 participants | **Low**  Valid measures used | **Low**  Self-reported with a cut-off point | **High**  Correlation | **High**  Some data not reported | **Unclear**  No information on the response rate |
| **Das 2021** | **High**  Some excluded, sampling method was not clearly specified but it seems a convenience sampling strategy was used | **High**  96 participants | **Low**  Factors obtained from medical records | **Low**  Self-reported and a cut-off point | **Low**  Non parametric tests and adjusted analysis | **Low**  All reported | **Low**  118 participants enrolled, 96 (81%) consented |
| **Dickinson 2022** | **High**  Some excluded: Not speaking English, aged <18 years, infant unlikely to survive/ died within the  1st week/admitted to NICU within 72 hours after birth, sampling strategy unclear | **Low**  114 participants | **Low**  Valid measures used | **Low**  Self-reported and a cut-off point | **High**  Difference between groups | **Low**  All reported | **High**  114 at first assessment, 79 at second assessment (31%) lost |
| **Feeley 2007** | **Low**  The study aim was to look at BW<1500 g, and the inclusion <1500 g and included English and French speaking. A random sampling strategy was used | **Low**  66 couples | **Low**  Valid measures used | **Low**  Self-reported and a cut-off point | **High**  Analysis of variance (ANOVA) | **Low**  All reported | **High**  88 at 3 months 62 at 9 months (26%) lost |
| **Fontoura 2018** | **High**  HIV+, deaf participants were excluded and a convenience sampling used | **High**  144 participants | **Low**  Valid measures used | **Low**  Self-reported and a cut-off point | **HIGH**  T test | **Low**  All reported | **Low**  115 approached 114 (99%) included |
| **Garfield 2015^a^** | **High**  Some excluded: Mothers <18 yrs old, ongoing critical illness (HIV, seizure), major depression, psychosis, bipolar disease, mothers to infants receiving mechanical ventilation.  A convenience sample of 113, part of a larger  randomized clinical trial | **Low**  113 participants | **Low**  Valid measure used | **Low**  Self-reported and a cut-off point | **High**  Correlation | **Low**  All reported | **Low**  All approached included |
| **Gennaro 1989** | **High**  Unclear sample strategy/excluding anomalies | **High**  41 participants | **Low**  Valid measures used | **Low**  Self-reported no cut-off point | **Low**  Multivariable analysis | **Low**  All reported | **High**  37% drop-off |
| **Greene 2015 & 2019** | **High**  Younger mother, babies unlikely to survive excluded.  “Of a subset of 100 eligible mothers from the larger study” | **High**  69 participants | **Low**  Valid measures used | **Low**  Self-reported with a cut-off point | **Low**  Multivariable logistic regression | **Low**  All data reported | **Low**  100 eligible mothers and 69 (69%) included |
| **Holditch-Davis 2009^a^** | **High**  Congenital anomalies, HIV+, drug users excluded.  A convenience sampling strategy used | **Low**  177 participants | **Low**  Valid measure used | **Low**  Self-reported with a cut-off point | **High**  Correlation | **Low**  All reported | **High**  177 enrolled 105 (60%) assessed |
| **Khemakhem 2020** | **High**  Unclear exclusion criteria and a convenience sampling was used | **High**  10 participants | **Low**  Valid measures used | **Low**  Self-reported with a cut-off point | **High**  Correlation | **Low**  All reported | **Unclear**  No information on how many invited |
| **Kong 2013** | **High**  Some excluded: parents with mental health problems excluded and a convenience sampling was used | **Low**  600 participants | **Low**  Valid measures used | **Low**  Self-reported with a cut-off point | **Low**  Multivariate regression analysis was  performed for significant factors  in the univariate analysis | **Low**  All reported | **Unclear**  There is no information on response rate |
| **Lotterman 2018^a^** | **High**  Did not include mothers who were unable to visit  the NICU and a convenience sampling strategy was used | **High**  91 participants | **Low**  Valid measure used to assess all factors including ability to cope and optimism | **Low**  Self-reported with a cut-off point | **Low**  Multivariable linear regression | **Low**  All reported | **Low**  Response rate 84% at time 2 |
| **Misund 2013 & 2014^a^** | **High**  Mothers of severely ill babies that the medical staff estimated to have poor chance of survival, and non-Norwegian speakers were not included and a convenience sampling was used | **High**  29 participants | **Low**  Valid measures used | **Low**  Self-reported with a cut-off point | **Low**  Associations then stepwise regression | **Low**  All reported | **Low**  29 of 34 families (85.3%) were included |
| **Moreyra 2021^a^** | **High**  None excluded,  But no information on the sampling strategy | **Low**  150 participants | **Low**  Gender and ethnicity factors | **Low**  Self-reported no cut-off point | **High**  Comparison analysis | **High**  Ethnicity data not reported | **Low**  158 eligible and 150 (95%) included |
| **Mulder 2014** | **Low**  No exclusion and a random sampling was used | **Low**  242 participants | **Low**  A gender factor | **Low**  Self-reported with a cut-off point | **High**  Comparison analysis | **Low**  All reported | **Low**  The retention rate at 2 years was 93% and between 9 months and 2 years 100% |
| **Okito 2022** | **High**  Not speaking English were excluded and a convenience sampling strategy used | **High**  45 participants | **Low**  Resilience using valid scale | **Low**  Self-reported with a cut-off point | **Low**  Multiple regression analyses | **Low**  All reported | **High**  First assessment 45 and 21 (47%) lost at the second assessment |
| **Park 2022** | **High**  Congenital anomaly and severely ill infants excluded, no random selection of participants | **High**  91 participants | **Low**  Valid scales for depression and physical health were used | **Low**  Self-reported no cut-off point | **High**  Correlation | **Low**  All reported | **Low**  93 agreed and 91 participated (98%) |
| **Pisoni 2020^a^** | **High**  Congenital anomalies maternal psychiatric illness and/or drug abuse all excluded, no random selection of the participants | **High**  29 participants | **Low**  Valid measures used | **Low**  Self-reported no cut-off point | **High**  Correlation | **Low**  All reported but data all reported | **Low**  All included in the analysis |
| **Rogers 2013** | **High**  Congenital anomalies and severely ill infants excluded, no information on sampling | **High**  73 participants | **Low**  Valid measures used | **Low**  Self-reported with a cut-off point | **High**  Correlation | **High**  Not all data reported fully | **Low**  83 eligible 73 competed (88%) |
| **Serge 2014** | **High**  Participants with missing data excluded and a convenience sampling strategy used | **Low**  200 participants | **Low**  Valid measures used | **Low**  Self-reported with a cut-off point | **High**  Correlatio**n** | **Low**  All reported | **High**  571 eligible 200 enrolled (response rate 30%) |
| **Shivhare 2022** | **High**  Mothers with serious  physical conditions excluded, a convenient sample was used | **Low**  100 participants | **Low**  Objective factors birthweight, gestational age, birth weight and Apgar scores | **Low**  Self-reported with a cut-off point | **High**  Differences between groups | **Low**  All reported | **Low**  All included in the analysis |
| **Treyvaud 2016** | **High**  Not completing the questioners and the sample was subset set of a cohort study | **Low**  162 participants | **Low**  Valid measures used | **Low**  Self-reported with a cut-off point | **Low**  Multivariable regression | **Low**  All reported | **Low**  130 (80%) responses |
| **Vizcarrondo-Oppenheimer, 2021** | **High**  Unclear exclusion criteria and participants were identified from hospital records | **High**  92 participants | **Low**  Valid measures used | **Low**  Self-reported with a cut-off point | **Low**  Logistic regression | **High**  Correlation data not reported, obstetric factors data were not reported | **Unclear**  No information on how many invited and how many responded |
| **Zanardo 1998** | **High**  Unclear exclusion criteria and a convenience sample strategy was used | **High**  55 participants | **Low**  Valid measures used | **Low**  Self-reported no cut-off point | **High**  Mean difference (MD) analysis | **Low**  All reported | **Low**  No drop-out |

**Abbreviations: ^a^** Studies included in both anxiety and post-traumatic stress**:** Garfield 2015, Greene 2015 & 2019, Holditch-Davis 2009, Lotterman 2018, Misund 2013 & 2014, Moreyra 2021, Pisoni 2022.
